# Little Patients, Big Tasks - A Pediatric Emergency Medicine Escape Room

**DOI:** 10.21980/J89W70

**Published:** 2023-10-31

**Authors:** Jessica Pelletier, Ernesto Romo, Bryan Feinstein, Charles Smith, Gina Pellerito, Alexander Croft

**Affiliations:** * Washington University, Department of Emergency Medicine, St. Louis, MO; ^ St. Louis Children’s Hospital, One Children’s Place, St. Louis, MO

## Abstract

**Audience:**

The target audience for this small group session is post-graduate year (PGY) 1–4 emergency medicine (EM) residents, pediatric EM (PEM) fellows, and medical students.

**Introduction:**

Pediatric emergency department visits have been declining since the start of the COVID-19 pandemic, leading to decreased exposure to pediatric emergency care for EM residents and other learners in the ED.^1^ This is a major problem, given that the Accreditation Council for Graduate Medical Education (ACGME) mandates that a minimum of 20% of patient encounters or five months of training time for EM residents must occur with pediatric patients, with at least 50% of that time spent in the ED setting.^2^,^3^ A minimum of 12 months must be spent in the pediatric ED for PEM fellows,^2^ and an average of 7.1 weeks of medical school are spent in pediatric clerkships.^4^ This decrease in pediatrics exposure in the post-pandemic environment can be addressed through simulation and gamification. We selected the gamification method of an escape room to create an engaging environment in which learners could interface with key pediatric emergency medicine clinical concepts via group learning.

**Educational Objectives:**

By the end of this small group exercise, learners will be able to:

1. Demonstrate appropriate dosing of pediatric code and resuscitation medications
2. Recognize normal pediatric vital signs by age
3. Demonstrate appropriate use of formulas to calculate pediatric equipment sizes and insertion depths
4. Recognize classic pediatric murmurs
5. Appropriately diagnose congenital cardiac conditions
6. Recognize abnormal pediatric electrocardiograms (ECGs)
7. Identify life-threatening pediatric conditions
8. Demonstrate intraosseous line (IO) insertion on a pediatric model
9. Demonstrate appropriate use of the Neonatal Resuscitation Protocol (NRP^®^) algorithms

**Educational Methods:**

An escape room - a form of gamification - was utilized to engage the learners in active learning. Gamification is an increasingly popular educational technique being utilized in graduate medical education and refers to the conversion of serious, non-trivial material into a fun activity fashioned like a game in order to enhance engagement in learning.^5^ This educational method seeks to enhance knowledge, attitudes, and skills via components of games - such as puzzles and prizes - outside of the context of a traditional game.^6^ Though high-quality research data on the effectiveness of gamification methods in graduate medical education is limited, studies have shown that gamification enhances learning, attitudes, and behaviors.^5^,^7^ One randomized, clinical-controlled trial investigating the use of gamification to enhance patient outcomes found that patients of primary care physicians randomized to the gamification group reached blood pressure targets faster than in the control group.^8^ Escape rooms as a modality for education have been suggested to improve active learning and enhance learner engagement in the learning process.^9^ In an escape room, learners are “locked” in an artificial environment (whether digitally or in person) and must utilize their group or individual knowledge to solve puzzles and escape from their “entrapment.”^9^,^10^ Escape rooms utilized as part of EM residency didactic training have demonstrated learner enthusiasm,^11^,^12^ desire to repeat the activity again,^13^ preference for escape rooms over traditional learning methods,^14^,^15^ improved confidence in communication and leadership skills,^11^,^15^ and improvement scores from pre- to post-testing.^16^

We developed an escape room in which learners were divided into teams and informed that they would need to “escape” from our resident lounge by successfully completing all nine stations. The first team to complete all nine stations would win a prize. Only after the last team completed the ninth station and debriefing was complete could all teams be “freed” from the escape room.

**Research Methods:**

Learners provided anonymous online survey feedback regarding the quality of the educational content and the efficacy of the delivery method.

**Results:**

A post-participation survey was disseminated to 55 residents, 32 of whom attended the PEM Escape Room, with a response rate of 9% (3/32 residents). One hundred percent of respondents felt that the activity content was applicable to their needs as an emergency physician. The session was rated as excellent by 33.3% of respondents, and 66.7% of respondents rated the session as above average. A second survey was disseminated seven months after the event to the 24 remaining residents who attended the event, with a response rate of 46% (11/24 residents); eight attendees had graduated at the time of this survey dissemination. Results of the second survey indicated that 100% (24/24 residents) felt that the activity content was applicable to their needs as an emergency physician, 73% (17/24 residents) rated the session as excellent, and 27% (7/24 residents) rated the session as above average.

**Discussion:**

Though we received limited survey responses (3/32 on the first survey and 11/24 on the second survey), respondents felt that the educational content met their learning needs and was of high quality. We had six faculty members present to facilitate the escape room while there were four groups of residents (eight per group). The ideal faculty to resident ratio would be one faculty member per group with three to six players, based on prior literature showing that teams of more than six players take longer to complete escape room tasks.^17^,^18^ We also recognized the importance of sending out the feedback survey link early because we believe the delay in our survey being emailed to the residents contributed to the low response rate (three trainees).

One participant provided the following feedback: “I think the ‘escape room’ struck an excellent balance with regard to trying to address knowledge that was relevant but also obscure or difficult enough that group/collaborative effort was required. I enjoyed the process and low stakes atmosphere.” This quote nicely summarizes our take-aways: That the PEM escape room incorporates key tenets of adult learning theory. Also known as andragogy, adult learning theory posits that adult learners are self-directed, have prior life experiences that shape their learning process, learn for practical reasons (ie, choose to learn in order to fulfill the demands of their social role), and are problem-oriented in their learning.^19^ Though andragogy does not technically apply only to adults (as many children are self-directed learners),^20^ having an understanding of the practical and experiential nature via which adults approach learning allows the adult educator to appropriately cater educational activities to meet the adult learner’s needs.

This escape room aligned with the core tenets of adult learning theory in several ways. Specifically, residents were given autonomy of participation in the escape room and thus had to take initiative to promote their own learning.^21^ Topics featured in the escape room stations were selected based on their clinical challenges and high-yield for board examinations and patient care, making their relevance immediately obvious to learners; this is a key feature of catering to adult learners.^22^ The escape room provided a comfortable and collegial environment in which residents felt comfortable learning, fostering an ideal setting for mature learners.^21^ Direct and immediate feedback are key components of adult learning theory, and faculty members were physically present to provide feedback at each escape room station.^22^ Finally, working in teams required the learners to engage in active learning rather than acting as passive recipients of cognitive information.^21^ Thus, the PEM escape room serves as an ideal framework to meet the needs of the adult learner.

**Topics:**

Pediatrics, emergency medicine, pediatric emergency medicine.

## USER GUIDE

[Table t1-jetem-8-4-sg1]

**Table t1-jetem-8-4-sg1:** 

List of Resources:	
Abstract	1
User Guide	4
Small Groups Learning Materials	8
Appendix A: Small Group Application Exercise	8
Appendix B: Small Group Application Exercise Answers	13
Appendix C: Wrap Up	18

**Learner Audience:**

Interns, Junior Residents, Senior Residents

**Time Required for Implementation**:

Instructor preparation: 60 minutes

Escape Room: 100 minutes (approximately 10 minutes per station with 10 minutes of extra time built in)

Debrief: 20 minutes for debriefing

**Recommended Number of Learners per Instructor**:

7 learners:1 facilitator. An overall session coordinator is recommended to help keep track of time and ensure that teams are following the rules of the game.

**Topics:**

Pediatrics, emergency medicine, pediatric emergency medicine.

**Objectives:**

By the end of this small group exercise, learners will be able to:

1. Demonstrate appropriate dosing of pediatric code and resuscitation medications
2. Recognize normal pediatric vital signs by age
3. Demonstrate appropriate use of formulas to calculate pediatric equipment sizes and insertion depths
4. Recognize classic pediatric murmurs
5. Appropriately diagnose congenital cardiac conditions
6. Recognize abnormal pediatric ECGs
7. Identify life-threatening pediatric conditions
8. Demonstrate IO insertion on a pediatric model
9. Demonstrate appropriate use of the NRP^®^

### Linked objectives and methods

In adult learning theory, learners cannot be forced to learn^22^ and are thought to need to understand the applicability of the material to their life and work; adult learners are seen as responsible for their own learning; adult learners build on past experiences; and the learners comes to the table ready to learn because they want to change their own circumstances. The educator’s role is to ensure engagement in the material so the learner can absorb what is important.^22^ Taylor and Hamdy describe the roles of the educator and the learner throughout the stages of learning from the perspective of adult learning theory. Instructors should help students recognize their own intrinsic desire to learn, create a scaffold from which trainees can learn, help learners integrate new content using their existing knowledge as a framework, help students reflect on their thoughts and actions, give feedback, and open the door for new opportunities in which the recipients of the education can apply their new knowledge.^22^

This conceptual framework was used to build an interactive learning opportunity in the form of an escape room. As a voluntary exercise, this provided an opportunity to build on the intrinsic motivation of learners. Active learning methods were utilized, including solving puzzles, working in groups, and performing procedures in order to “escape” from various stations (and ultimately from the resident lounge). Facilitators explained to participants that the content of the escape room was selected based upon its relevance to clinical practice. Facilitators helped build on residents’ prior knowledge to allow them to come to the answers at the stations. Debriefing at the end of the escape room also allowed for learner reflection.

Educational Objective (EO) 1: Demonstrating appropriate dosing of pediatric code and resuscitation medications was met in Station 1 - Pediatric Resuscitation - in which residents had to successfully resuscitate a 6-year-old male arriving in pulseless electrical activity after drowning. Epinephrine was indicated on arrival of the patient to the ED. The patient transitioned to ventricular fibrillation requiring defibrillation, and learners were expected to appropriately select the voltage used for defibrillation as well as the doses of amiodarone or lidocaine. After achieving return of spontaneous circulation (ROSC), the patient went into supraventricular tachycardia, requiring administration of adenosine or synchronized cardioversion. Learners were permitted to use the PediSTAT^®^ application to look up the doses for defibrillation/cardioversion and medications in Station 1.

EO 2: Recognizing normal pediatric vital signs by age was accomplished in Station 2 - Normal Vital Signs. Residents were expected to match specific pediatric age ranges (neonate, infant, toddler, preschool, school age, and adolescent) with corresponding sets of normal vital signs for that age range. Residents were not permitted to use any references or cognitive aids for this station.

EO 3: Demonstrating appropriate use of formulas to calculate pediatric equipment sizes and insertion depths was accomplished in Station 3 - Pediatric Equipment Sizes. In this station, a one-year-old male is brought to the ED in cardiac arrest and requires placement of an endotracheal tube, naso-or orogastric tube, foley catheter, and central line. Learners were permitted to use the PediSTAT^®^ application to aid in determining the equipment sizes and had to select which grid pattern contained the appropriate equipment sizes for this patient. After selecting the appropriate equipment grid, learners had to calculate the depth of insertion for each device using standard formulas. Participants were required to solve both parts of this station on their own before facilitators could confirm whether they got the answers correct.

EO 4: Recognizing classic pediatric murmurs was addressed in Station 4 - Pediatric Murmurs. Learners were given vignettes of pediatric patients with congenital cardiac defects and were expected to match the clinical scenarios and murmur descriptions with the associated murmurs.

EO 5: Appropriately diagnosing congenital cardiac conditions was accomplished in Station 4 (discussed above) and in Station 5 - Congenital Cardiac Conditions. Station 5 required learners to match x-rays of congenital cardiac defects with their associated diagnoses. These diagnoses included transposition of the great vessels, tetralogy of Fallot, Ebstein anomaly, truncus arteriosus, coarctation of the aorta, and dextrocardia.

EO 6: Recognizing abnormal pediatric electrocardiograms (ECGs) was met via Station 6 - Peds Patient with Syncope - in which learners were expected to match Brugada syndrome, arrhythmogenic right ventricular dysplasia (ARVD), and Wolff- Parkinson-White (WPW) syndrome with their corresponding ECGs. Normal pediatric ECGs were also included in this station to ensure that learners could distinguish normal versus abnormal ECG characteristics.

EO 7: Identifying life-threatening pediatric conditions was accomplished via Stations 1 and 4–6, all of which have been discussed previously. Station 1 required learners to recognize the need for resuscitation, and Stations 4–6 involved the identification of potentially life-threatening cardiac conditions. Station 7 also met EO 7, requiring participants to identify life-threatening conditions such as tracheomalacia, medulloblastoma, traumatic brain injury, sepsis, and Kawasaki disease.

EO 8: Demonstrating IO insertion on a pediatric model was accomplished via Station 8 - We Can’t Get Access, Doc! In this station, learners were provided with a vignette involving a pediatric patient in status asthmaticus who subsequently develops hypoxia and PEA arrest. The patient does not have intravenous (IV) access prior to this turn of events, and learners are expected to recognize the need for IO line insertion. This provided an opportunity for residents to discuss indications for IO placement, appropriate insertion sites, and to practice IO insertion on a pediatric model. Adult-size needles were also available for practice in the event of treating pediatric patients with elevated body mass index (BMI).

EO 9: Demonstrating appropriate use of the NRP^®^ algorithms was accomplished in Station 9 - NRP^®^. In this station, learners completed a crossword puzzle by solving clinical questions using NRP^®^. Participants were permitted to look up the NRP^®^ algorithms in order to solve the questions that were posed.

### Recommended pre-reading for facilitator

The following are supplemental materials that can be considered for pre-reading by the facilitator or to have available as a reference:

- Shaw KN, Bachur RG, eds. *Fleisher & Ludwig’s Textbook of Pediatric Emergency Medicine*. 8th edition. Wolters Kluwer; 2021.
- Weiner MD, ed. *NRP*^*®*^ *Textbook of Neonatal Resuscitation*. 8th ed. American Academy of Pediatrics; 2021.

### Learner responsible content (LRC)

Learners are strongly encouraged to review the following:

- Normal pediatric vital signs by age:
  ○ Novak C, Gill P. Pediatric Vital Signs Reference Chart. PedsCases. Published March 2020. Accessed February 18, 2023. At: https://www.pedscases.com/sites/default/files/Vitals%20Chart_PedsCases%20Notes.pdf
- NRP^®^ algorithm:
  ○ American Academy of Pediatrics. Neonatal Resuscitation Program^®^ 8th Edition Algorithm. Accessed August 20, 2023. At: https://nrplearningplatform.com/instructor-toolkit/assets/Instructor_ToolKit/RESOURCES/DocumentsAndForms/resources/NRP%208th-Ed%20ITK%20Algorithm%20w%20logos.pdf

### Required Materials

The following is the list of materials you will need:

- IO needles of varying sizes, IO insertion device (such as EZ-IO), and associated task trainer (chicken legs may be utilized if a synthetic task trainer is unavailable)
- PEM Escape Room Slide Deck^*^
  ○ See associated file.
  ○ ^*^Written permission for use of this template was obtained from Dr. Boysen-Osborn. The template was originally published in: Boysen Osborn M, Paradise S, Suchard J. The toxiscape hunt: an escape room-scavenger hunt for toxicology education. At: DOI:https://doi.org/10.21980/J8NW58
- PEM Escape Room Answer Key for Instructors
  ○ See associated file.
- Prize for the winning team
- Projector (for Station 9)
- Scissors
- Tape

### Results and tips for successful implementation

We tested the PEM escape room on 32 EM residents during regularly-scheduled didactics in January 2023. The escape room was paired with a pediatric simulation session. Each station required printing out paper and using printer ink (please see supplemental materials), but this was provided by our institution and did not impose any direct costs. The winning team received a bag of sweets, which was donated by a member of our faculty.

A post-participation feedback survey was emailed to residents after the PEM Escape Room session, which resulted in a response rate of only 9% (3/32). Though the response rate was limited, 100% of respondents felt that the activity content was applicable to their needs as an emergency physician. The session was rated as excellent by 33.3% of the respondents, and 66.7% of respondents rated the session as above average.

A second survey was disseminated in August 2023 (seven months after the initial curriculum implementation) to the 24 EM residents (PGY-2/3/4) who attended the session in January in an effort to obtain more survey responses. On this repeat survey, 100% (24/24 residents) felt that the activity content was applicable to their needs as an emergency physician, 73% (17/24) strongly agreed, 27% (7/24) somewhat agreed, 55% (13/24 residents) rated the session as excellent, and 45% (11/24 residents) rated the session as above average. Overall, the combined results of the first and second surveys indicate that residents found the PEM Escape Room applicable to their daily work with pediatric patients and provided positive global evaluations of the session.

Based on our experience, this session is best implemented during regularly-scheduled EM didactics in order to maximize attendance and can be paired with simulation for higher-level application of learning gleaned from the escape room. Our session attendance was good, with 58% (32/55) EM residents present. Attendance could be maximized by inviting PEM fellows and medical students to attend the session in the future.

The biggest limitation of our curriculum evaluation survey was the low number of initial respondents, which required us to repeat the survey in a delayed fashion. Incentives for survey response may be necessary to ensure a more robust number of responses during future iterations of this curriculum. Requiring survey completion in order to obtain credit for attendance is one additional way that the survey response rate could be enhanced. Likert-scale survey data is also limited in its ability to assess learner outcomes, given that it only assesses participant attitudes. We intend to conduct multiple-choice question (MCQ) testing to assess knowledge gained, as well as skill-based evaluation such as standardized simulation, during future iterations of this curriculum.

### Suggestions for Further Reading

The following materials for further exploration, watching, and listening are recommended for residents:

1. Kempema J. PediSTAT^®^ app. At: https://www.pedistat.com/
2. Emergency Medicine Reviews and Perspectives. EM:RAP. Pediatric EM Fundamentals, 2020. Accessed February 16, 2023. At: https://www.emrap.org/event/pediatric-em-fundamentals
3. Fox S. Pediatric ECG. emDocs.net. Published online July 26, 2019. Accessed February 16, 2023. At: http://www.emdocs.net/pediatric-ecg/
4. American Academy of Pediatrics. NRP^®^ Skills Videos. Published online April 12, 2022. Accessed February 16, 2023. At: https://www.aap.org/en/learning/neonatalresuscitation-program/nrp-skills-videos/

## Supplementary Information







## Figures and Tables

**Figure f1-jetem-8-4-sg1:**
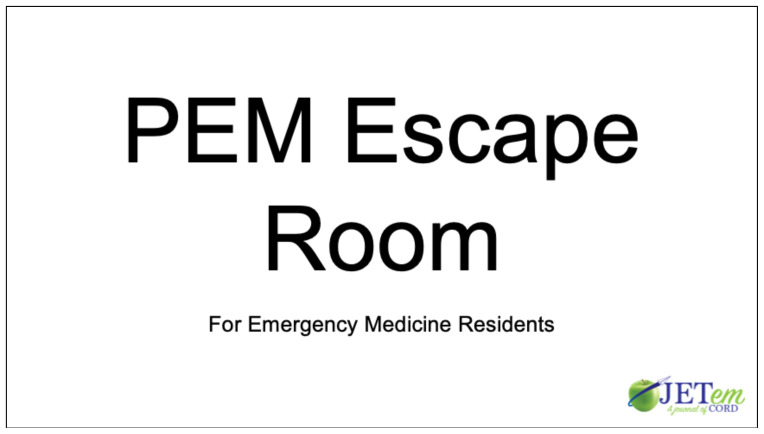
Please see associated PowerPoint file

**Figure f2-jetem-8-4-sg1:**
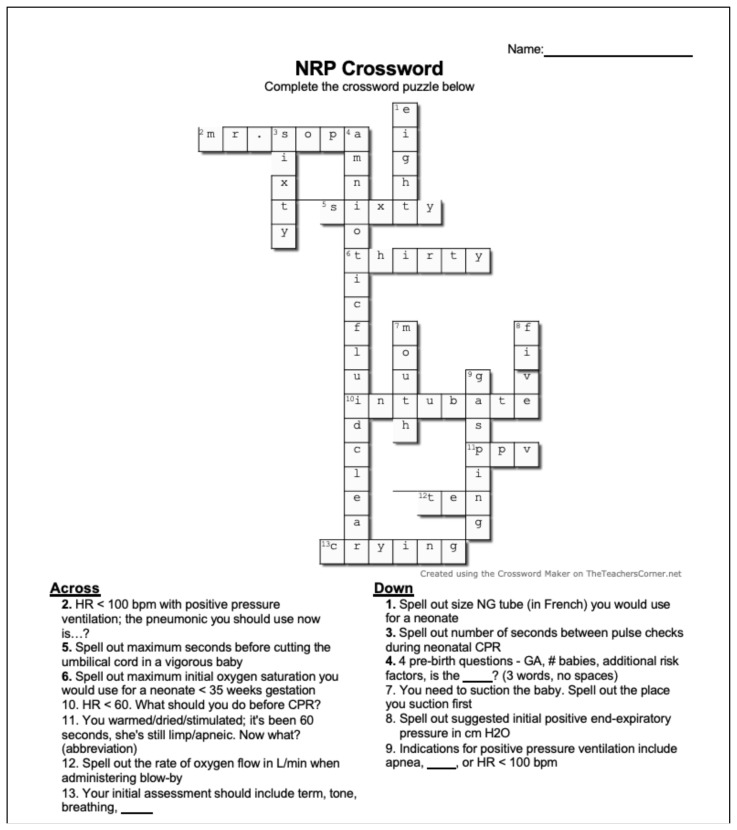
Please see associated PowerPoint file

**Figure f3-jetem-8-4-sg1:**
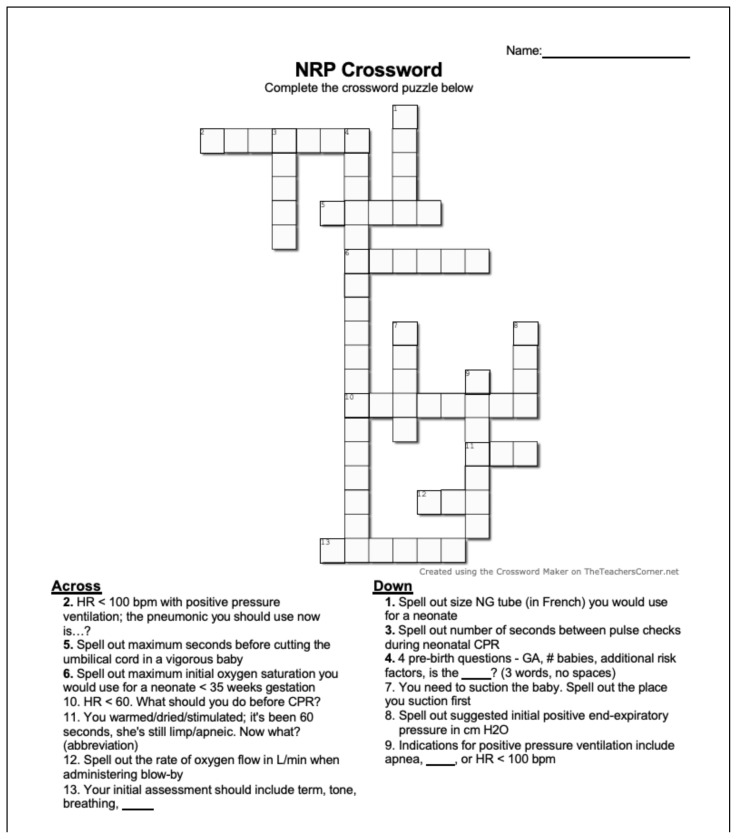
Please see associated PowerPoint file

**Figure f4-jetem-8-4-sg1:**
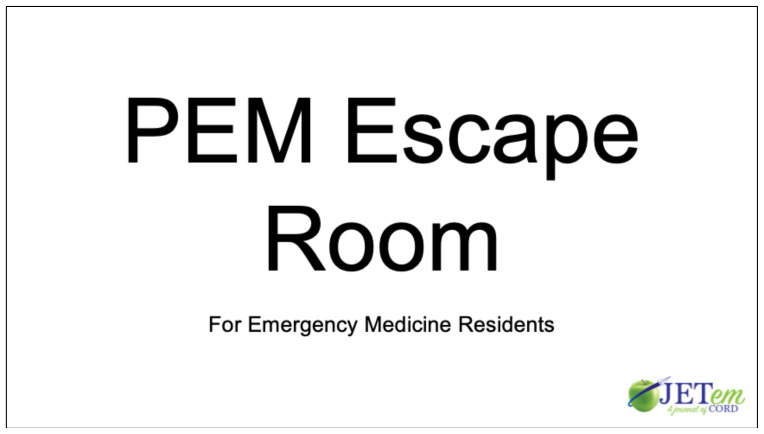
Please see associated PowerPoint file

**Figure f5-jetem-8-4-sg1:**
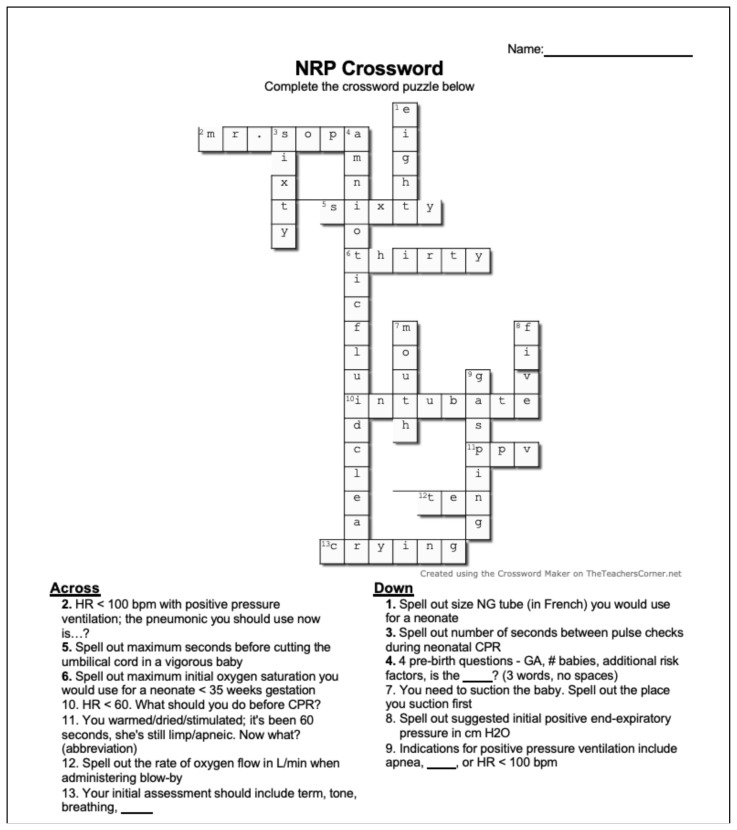
Please see associated PowerPoint file

**Figure f6-jetem-8-4-sg1:**
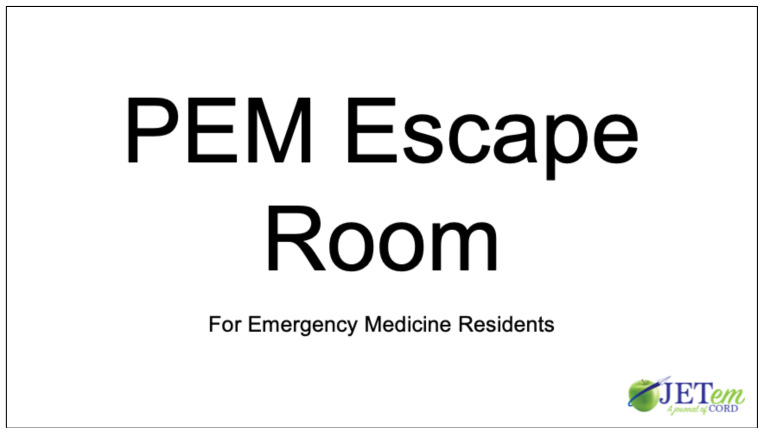
Please see associated PowerPoint file

## SMALL GROUPS LEARNING MATERIALS

### Appendix A Small Group Application Exercise (sGAE)

The following is the list of materials you will need:

- IO needles of varying sizes, IO insertion device (such as EZ-IO), and associated task trainer (chicken legs may be utilized if a synthetic task trainer is unavailable)
- PEM Escape Room Slide Deck[Fig f1-jetem-8-4-sg1]
  ○ ^*^Written permission for use of this template was obtained from Dr. Boysen-Osborn. The template was originally published in: Boysen-Osborn M, Paradise S, Suchard J. The toxiscape hunt: an escape room-scavenger hunt for toxicology education. At: DOI:https://doi.org/10.21980/J8NW58
- PEM Escape Room Answer Key for Instructors[Fig f2-jetem-8-4-sg1]
- Prize for the winning team
- Projector (for Station 9)
- Scissors
- Tape

#### Station Setup

Residents should designate a team “scribe” to write down the answers to the questions at each station. The scribe will be responsible for keeping track of which stations the team has completed.

One faculty member should be available to check answers for each group at any given time. Try to avoid having two groups at a single station at once. A team receives a printed ticket for each station they complete (make sure to print enough copies of Slide 84 so that you have at least nine tickets per group - this ensures that you will have enough if some are lost). The residents may complete Stations 1–8 in ANY ORDER, but Station 9 must come last. Thus, a team must have eight printed tickets before they can move to Station 9.

If participants are completing a multi-question station, facilitators may tell them which questions they got right and wrong. The team will have to correct the wrong answers until ALL answers are correct before they can move on to the next station. Residents may use their phones to look up answers EXCEPT for the vital sign station, or if the station indicates otherwise. A team may NOT move on from a station if they have not gotten all of the answers at the station correct. They must remain at that station and continue trying until all answers are corrected. Hints may only be given by a facilitator if a team continues to struggle, and this is holding up another team from entering the station. As a reminder, teams must complete Stations 1–8 before moving on to Station 9; thus, it is rare that hints will need to be given, since waiting teams should be able to identify one or more other stations they have not completed while they wait for a team who is struggling.

1. **Station 1** - *Pediatric Resuscitation - Megacode*. Slides 3–9 should be printed and taped to a wall. This station involves a pediatric megacode in which the patient progresses from pulseless electrical activity (PEA) to ventricular fibrillation, followed by supraventricular tachycardia (SVT) and unstable sinus bradycardia. Residents may use the PediSTAT^®^ application on their phones to look up the doses for medications.
2. **Station 2** - *Normal Vital Signs (OMG!!!).* Slides 10–21 should be printed and taped to a wall. Residents must match each normal vital sign to the correct pediatric age group. Residents may NOT use their phones for this exercise. They do not need to complete the children in order.
3. **Station 3** - *Pediatric Equipment Sizes.* Slides 22–42 should be printed and taped to a wall. Residents may use the PediSTAT^®^ application on their phones for this section.
  - For Part 1 (Slides 23–40), residents must identify which pattern matches the correct equipment sizes for a 1-year-old male patient. Facilitators should confirm the correct answer before residents may move on to Part 2.
  - For Part 2 (Slide 41), residents must select the appropriate insertion depth for each piece of equipment that they chose in Part 1.
  - For Part 3 (Slide 42), residents must troubleshoot hypoxia in an intubated pediatric patient.
4. **Station 4** - *Pediatric Murmurs.* Slides 43–49 should be printed and taped to a wall. Residents will need to match the murmur in each vignette to its appropriate identity on Slide 44. They may look up answers on their phones.
5. **Station 5** - *Congenital Cardiac Conditions.* Slides 50–56 should be printed and taped to a wall. Residents will need to name the congenital cardiac condition depicted in each chest x-ray. They may look up answers on their phones.
6. **Station 6** - *Peds Patient with Syncope.* Slides 57–62 should be printed and taped to a wall. Residents will need to name the disorder (if any) depicted in each ECG. They may look up answers on their phones.
7. **Station 7** - *Pediatric Boards Fodder and Clinical Decision Rules.* Slides 63–73 should be printed and taped to a wall. Residents will need to identify the correct answer for each Case. They may look up answers on their phones. They do not need to complete the Cases in order.
8. **Station 8** - *We Can’t Get Access, Doc!* Slides 74–77 should be printed and taped to a wall. Residents will start by answering questions related to proper IO insertion and pain management. They may look up answers on their phones. IO insertion needles of varying sizes, insertion devices (such as EZ-IO^®^), and a task trainer will be needed for this station. The facilitator should demonstrate proper insertion of the IO needle using the insertion device available. Each resident should be given the chance to insert all sizes of needle before completing this station.
9. **Station 9** - *LAST STATION - NRP*^*®*^. Residents will fill out a crossword puzzle using the NRP^®^ algorithm and guidelines. They may look up answers on their phones. There are several options for how to set up this Station depending upon your space and equipment constraints. Print OR project Slide 81 (we recommend projecting for better viewing). Print Slides 79–80 and tape to the wall (optional). Print one copy of the following document for each resident so that they can fill in the crossword puzzle on their own:[Fig f3-jetem-8-4-sg1]
  The blue highlighted shapes on the crossword puzzle spell out “FRIDGE.” A prize (ideally an edible one) should be hidden in the closest staff refrigerator. Tape a printed copy of Slide 83 to the prize. The first team to complete the crossword puzzle receives the reward.

### Appendix B sGAE Answers

Answers for each station can also be found in the Speaker Notes of the slidedeck and in the Answer Key.[Fig f4-jetem-8-4-sg1][Fig f5-jetem-8-4-sg1]

As a general rule, residents have unlimited attempts at each station. Facilitators may only answer “yes” or “no” in response to resident suggested answers.

1. **Station 1** - *Pediatric Resuscitation - Megacode*
  - Scenario 1 - 0.01 milligrams (mg)/kg, 0.1 milliliters (ml)/kg of epinephrine 1:10,000 (0.1 mg/ml) IV/IO
  - Scenario 2 - 2 Joules/kilogram (J/kg)
  - Scenario 3 -
    - 4 J/kg
    - Amiodarone 5 mg/kg or lidocaine 1 mg/kg intravenous (IV) or IO
    - Maximum of three doses of amiodarone
  - Scenario 4 -
    - 220 minus age in years
    - Supraventricular tachycardia (SVT)
    - Adenosine 0.2 mg/kg IV/IO
    - Synchronized cardioversion at 0.5–1 J/kg
  - Scenario 5 - Atropine 0.02 mg/kg IV/IO
2. **Station 2** - *Normal Vital Signs (OMG!!!)*
  - Child #1 - D - Preschool (3–5 years)
  - Child #2 - C - Toddler (1–2 years)
  - Child #3 - E - School age (6–11 years)
  - Child #4 - A - Neonate (< 28 days)
  - Child #5 - E - School age (6–11 years)
  - Child #6 - E - School age (6–11 years)
  - Child #7 - D - Preschool (3–5 years)
  - Child #8 - F - Adolescent (12–15 years)
  - Child #9 - C - Toddler (1–2 years)
  - Child #10 - D - Preschool (3–5 years)
3. **Station 3** - *Pediatric Equipment Sizes*
  - Part 1 - The Case
    ○ Pattern 9 – 3.5, 8–10, 8–10, 3
  - Part 2 - The Depths
    - 10.5 cm (3.5 mm × 3)
    - Nose > ear > xiphoid process
    - Corner of the mouth > ear > xiphoid process
    - 7.3 cm
  - Part 3 - The Complication
    - DOPE - Displacement, Obstruction, Pneumothorax, Equipment
    - Pneumothorax - appropriate size chest tube is 16–20 French (if using large-bore)
4. **Station 4** - *Pediatric Murmurs*
  - Case 1 - G - Stills murmur (common benign systolic ejection murmur that is louder when the patient is tachycardic)
  - Case 2 -
    - I - Transposition of the great arteries
    - Prostaglandin E1 0.05–0.1 micrograms (mcg)/kg/minute (min)
  - Case 3 -
    - D - Ventricular septal defect (VSD)
    - Yes; untreated VSD can lead to Eisenmenger syndrome
  - Case 4 - H - Tetralogy of Fallot
  - Case 5 - J - Venous hum (common benign murmur that is more prominent with tachycardia)
5. **Station 5** - *Congenital Cardiac Conditions*
  - Case 1 - Transposition of the great vessels - “egg on a string”
  - Case 2 - Tetralogy of Fallot - “boot-shaped heart”
  - Case 3 - Ebstein anomaly - cardiomegaly with prominent right heart
  - Case 4 - Truncus arteriosus - right-sided aortic arch (seen in around 40% of cases). May have pulmonary plethora and cardiomegaly
  - Case 5 - Coarctation of the aorta - rib notching seen on the right
  - Case 6 - Dextrocardia
6. **Station 6** - *Peds Patient with Syncope*
  - ECG 1 - Normal
  - ECG 2 - Brugada syndrome, characterized by anterior ST segment elevation and incomplete right bundle branch block pattern
  - ECG 3 - Arrhythmogenic right ventricular dysplasia (ARVD), characterized by the epsilon wave
  - ECG 4 - Normal
  - ECG 5 - Wolff-Parkinson-White (WPW), characterized by the delta wave and long QT
7. **Station 7** - *Pediatric Boards Fodder and Clinical Decision Rules*
  - Case 1 - Tracheomalacia
  - Case 2 - Medulloblastoma; heel-to-shin (lower extremity ataxia > upper extremity)
  - Case 3 - Frontal
  - Case 4 - Yes, she fell > 5 feet. Other severe mechanisms include:
    ○ Moving vehicle collision (MVC) with ejection, death of another passenger, or rollover
    ○ Pedestrian or bicyclist without a helmet struck by a motorized vehicle
    ○ Head struck by a high-impact object
  - Case 5 - Full sepsis workup with urine cultures, blood cultures, chest x-ray, likely lumbar puncture (LP), and admission
  - Case 6 - Procalcitonin, C-reactive protein (CRP), absolute neutrophil count (ANC)
  - Case 7 - This is incomplete Kawasaki’s. You need CRP >/= 3 mg/deciliter (dL) and/or erythrocyte sedimentation rate (ESR) >/= 30 mmHg with three or more of the following:
    ○ Anemia for age
    ○ Platelets >/= 450,000 after 7 days of fever
    ○ Albumin </= 3 grams (g)/dL
    ○ Elevated alanine aminotransferase (ALT)
    ○ White blood cell count (WBC) >/= 15,000 WBC/microliter (μL)
    ○ Urine WBC >/= 10 WBC/high-power field (HPF)
    OR
    Positive ECHO
  - Case 8 - Air enema
  - Case 9 - Manual detorsion (“opening the book”), consult urology, scrotal ultrasound
  - Case 10 - Ovarian torsion
8. **Station 8** - *We Can’t Get Access, Doc!*
  - Epiphyseal plate
  - 2–5 mL
  - 0.5 mg/kg
9. **Station 9** - *LAST STATION - NRP*^*®*^
  ACROSS
  2. MR. SOPA (no spaces)
  5. Sixty
  6. Thirty
  10. Intubate
  11. Positive pressure ventilation (PPV)
  DOWN
  1. Eight
  3. Sixty
  4. Amniotic fluid clear (no spaces)
  7. Mouth
  8. Five
  9. Gasping

### Appendix C: Wrap Up

Facilitators should spend 20 minutes debriefing the PEM Escape Room. It is easiest for the facilitators to project the PEM Escape Room slide deck and go through the cases one at a time, polling the audience, correcting misunderstandings, and ensuring that all questions are answered.[Fig f6-jetem-8-4-sg1]

Pearls:

- Pearl #1 - Use the PediSTAT^®^ application or another bedside tool such as the Broselow™ tape to ensure accurate dosing of pediatric resuscitation medications, particularly epinephrine. You should have the following memorized:
  ○ Maximum heart rate can be calculated using 220 minus age in years.
  ○ Start at 2 J/kg for defibrillation, and double it to 4 J/kg if this is ineffective.
  ○ 0.5–1 J/kg is the appropriate dose for synchronized cardioversion.
- Pearl #2 - Keep a guide with normal pediatric vital signs in your pocket. You should have the following memorized:
  ○ 70 + (2 × age in years) = minimum systolic blood pressure.
- Pearl #3 - Use the PediSTAT^®^ application or another bedside tool such as the Broselow™ tape to ensure appropriate weight- or length-based equipment sizing. You should have the following memorized:
  ○ Endotracheal tube (ETT) diameter multiplied by three gives you the appropriate ETT insertion depth.
  ○ Nasogastric and orogastric tube depth should be measured from the tip of the nose or corner of the mouth (respectively), then to the ear, and down to the xiphoid process.
  ○ The mnemonic “DOPES” (Displacement, Obstruction, Pneumothorax, Equipment, Stiff Lungs) can help you troubleshoot the root cause of hypoxia in the intubated patient.
- Pearl #4 - Listen carefully for pediatric murmurs, since the presentation of non-cyanotic congenital heart diseases may be delayed.
- Pearl #5 - Evaluate pediatric chest x-rays for pulmonary volume overload, cardiomegaly, abnormal contours/morphology of the heart or mediastinum, and rib notching.
- Pearl #6 - Normal pediatric electrocardiograms (ECGs) may demonstrate rightward axis deviation or T-wave inversions in the anterior leads. In pediatric patients with syncope (especially on exertion), evaluate carefully for abnormal intervals (ie, QTc prolongation, bundle branch blocks), delta waves, epsilon waves, left ventricular hypertrophy, and dagger-like Q-waves.
- Pearl #7 - Miscellaneous takeaways:
  ○ Supine stridor in the neonate or infant is a red flag for tracheomalacia.
  ○ Medulloblastoma will present with lower greater than upper extremity ataxia.
  ○ Occipital, parietal, and temporal hematomas are red flags that raise concern for traumatic brain injury. The PECARN clinical decision rule should be used to determine which pediatric patients with head injury require contrast tomography (CT) imaging.
  ○ The Step-by-Step Approach to Febrile Infants can be used in well-appearing infants less than 90 days of age presenting with fever and no clear source.
  ○ Consider incomplete Kawasaki disease in pediatric patients with prolonged fever and no obvious source.
  ○ The whirlpool sign of the mesentery on ultrasound is classic for intussusception.
  ○ Do not forget to consider testicular or ovarian torsion in the differential for the inconsolable neonate/infant or the pediatric patient with abdominal pain.
- Pearl #8 - Have a low threshold to obtain IO access in the critically ill pediatric patient. Avoid hitting the growth plate during insertion.
- Pearl #9 - Use the “MR. SOPA” mnemonic before moving to cardiopulmonary resuscitation in the neonate, since most cardiac arrest in neonates is due to inadequate respiration.

The following materials for further exploration, watching, and listening are recommended for residents:

1. Kempema J. PediSTAT^®^ app. At: https://www.pedi-stat.com/
2. Emergency Medicine Reviews and Perspectives (EM:RAP). Pediatric EM Fundamentals; 2020. Accessed February 16, 2023. At: https://www.emrap.org/event/pediatric-em-fundamentals
3. Fox S. Pediatric ECG. emDocs.net. Published online July 26, 2019. Accessed February 16, 2023. At: http://www.emdocs.net/pediatric-ecg/
4. American Academy of Pediatrics. NRP^®^ Skills Videos. Published online April 12, 2022. Accessed February 16, 2023. At: https://www.aap.org/en/learning/neonatal-resuscitation-program/nrp-skills-videos/
