# Supplementary material for: Little Patients, Big Tasks - A Pediatric Emergency Medicine Escape Room
Source: J Educ Teach Emerg Med. 2023 Oct 31;8(4):SG1–SG19. doi: 10.21980/J89W70 (PMC10631808; doi:10.21980/J89W70)
Supplement: Supplementary file 1 [file jetem-8-4-sg1-supp1.pptx]

## Slide 1
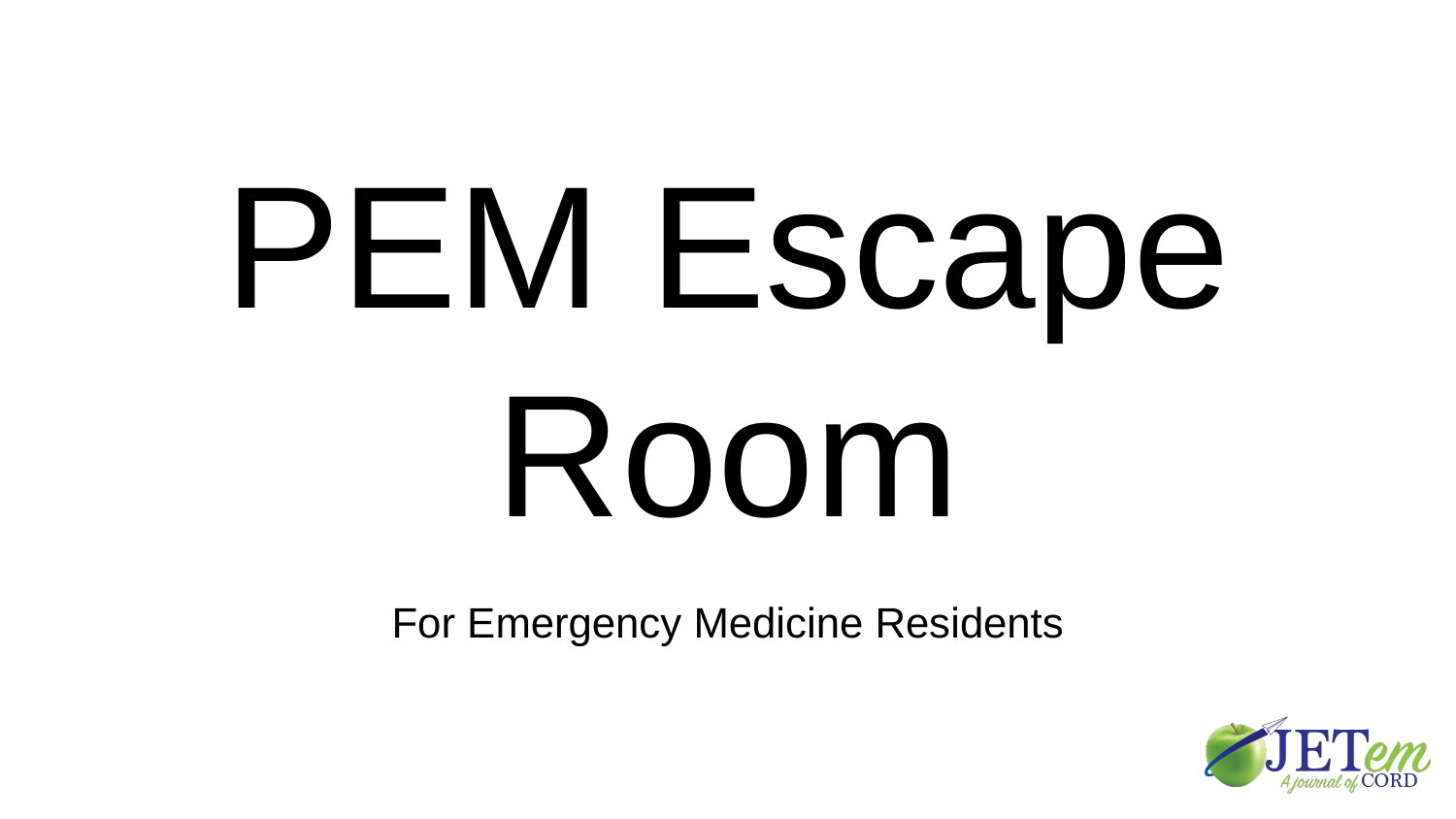

# PEM Escape Room
For Emergency Medicine Residents

## Slide 2
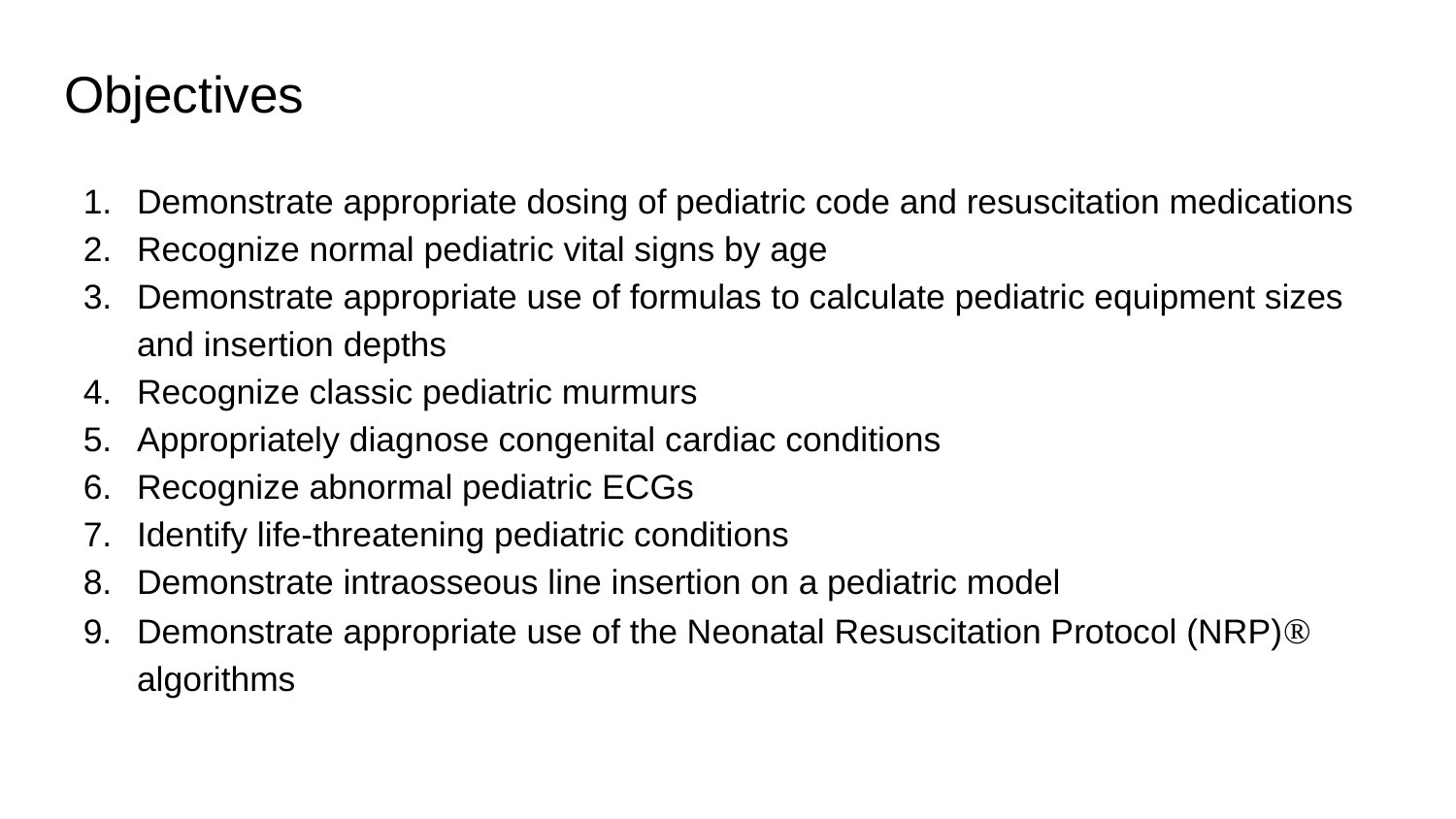

# Objectives
Demonstrate appropriate dosing of pediatric code and resuscitation medications
Recognize normal pediatric vital signs by age
Demonstrate appropriate use of formulas to calculate pediatric equipment sizes and insertion depths
Recognize classic pediatric murmurs
Appropriately diagnose congenital cardiac conditions
Recognize abnormal pediatric ECGs
Identify life-threatening pediatric conditions
Demonstrate intraosseous line insertion on a pediatric model
Demonstrate appropriate use of the Neonatal Resuscitation Protocol (NRP)® algorithms

## Slide 3
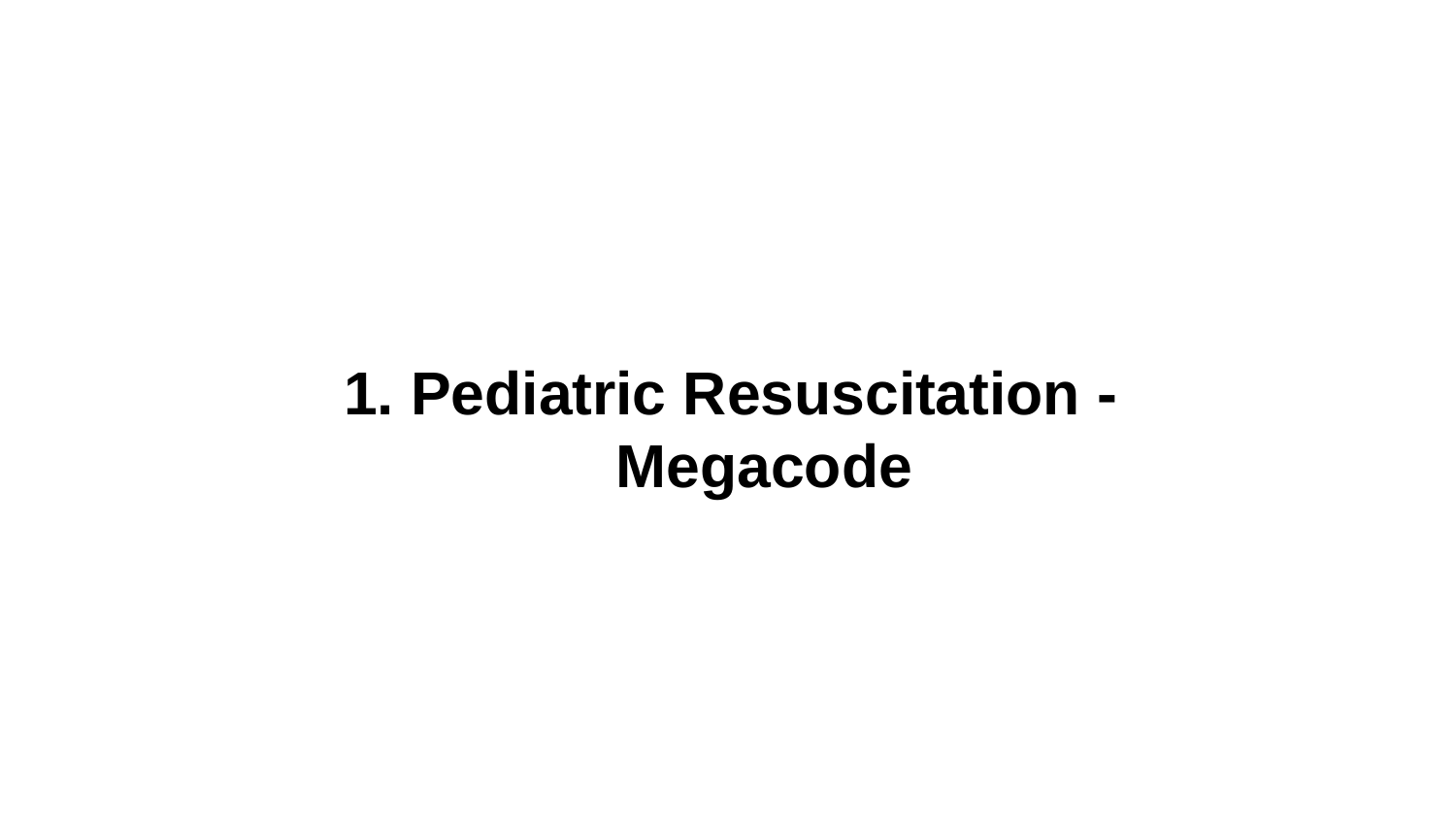

Pediatric Resuscitation - Megacode

## Slide 4
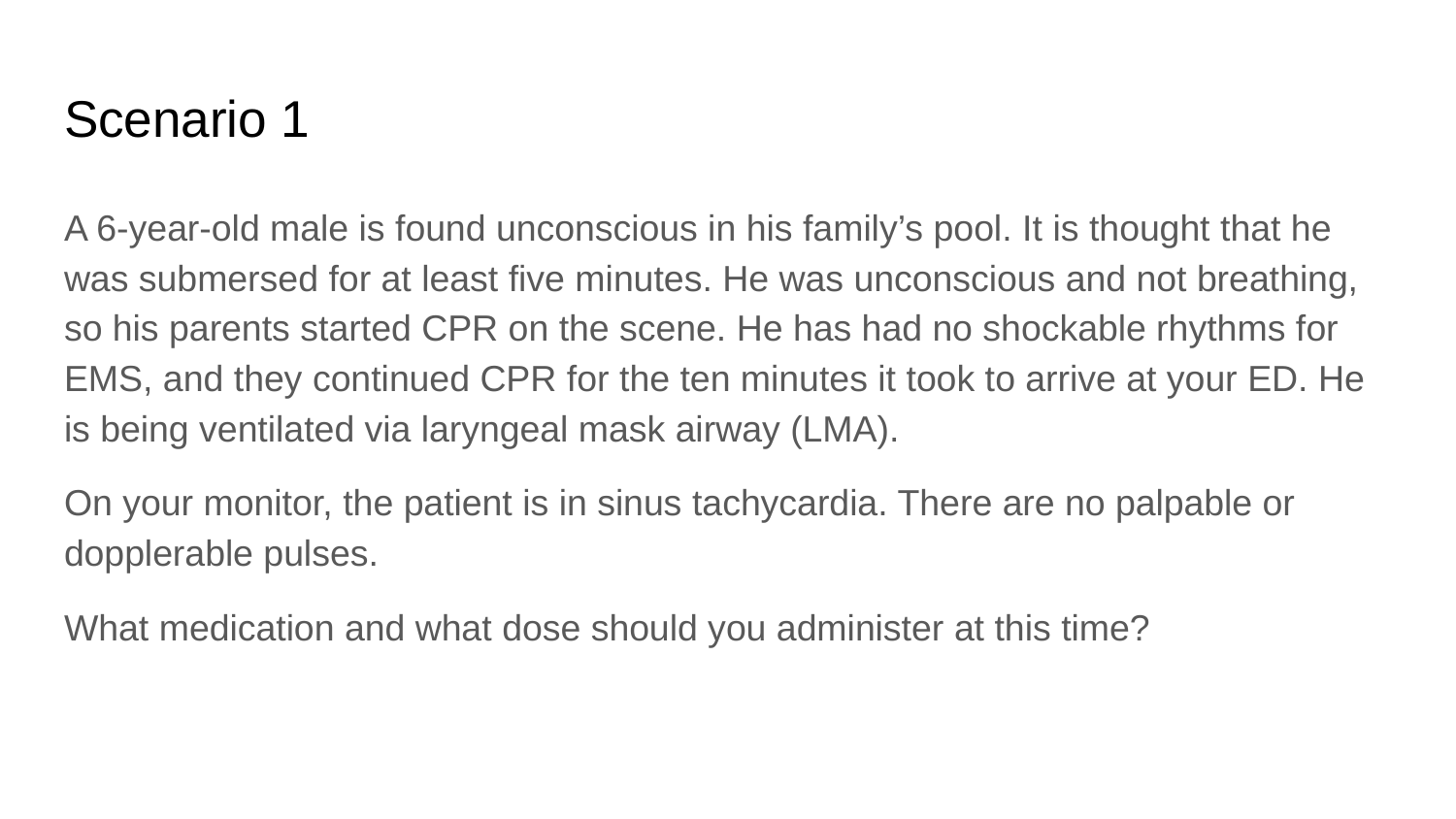

# Scenario 1
A 6-year-old male is found unconscious in his family’s pool. It is thought that he was submersed for at least five minutes. He was unconscious and not breathing, so his parents started CPR on the scene. He has had no shockable rhythms for EMS, and they continued CPR for the ten minutes it took to arrive at your ED. He is being ventilated via laryngeal mask airway (LMA).
On your monitor, the patient is in sinus tachycardia. There are no palpable or dopplerable pulses.
What medication and what dose should you administer at this time?

## Slide 5
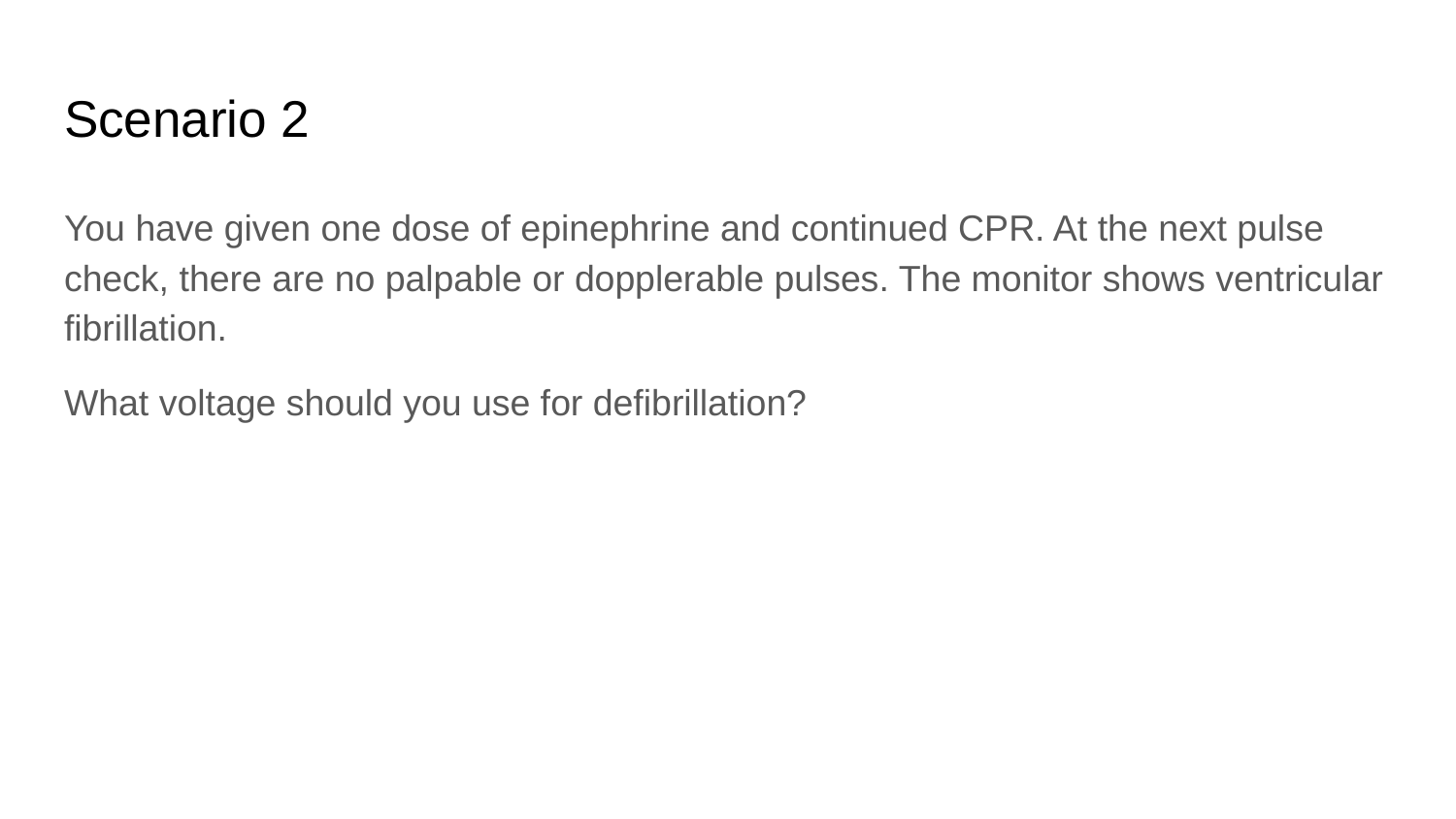

# Scenario 2
You have given one dose of epinephrine and continued CPR. At the next pulse check, there are no palpable or dopplerable pulses. The monitor shows ventricular fibrillation.
What voltage should you use for defibrillation?

## Slide 6
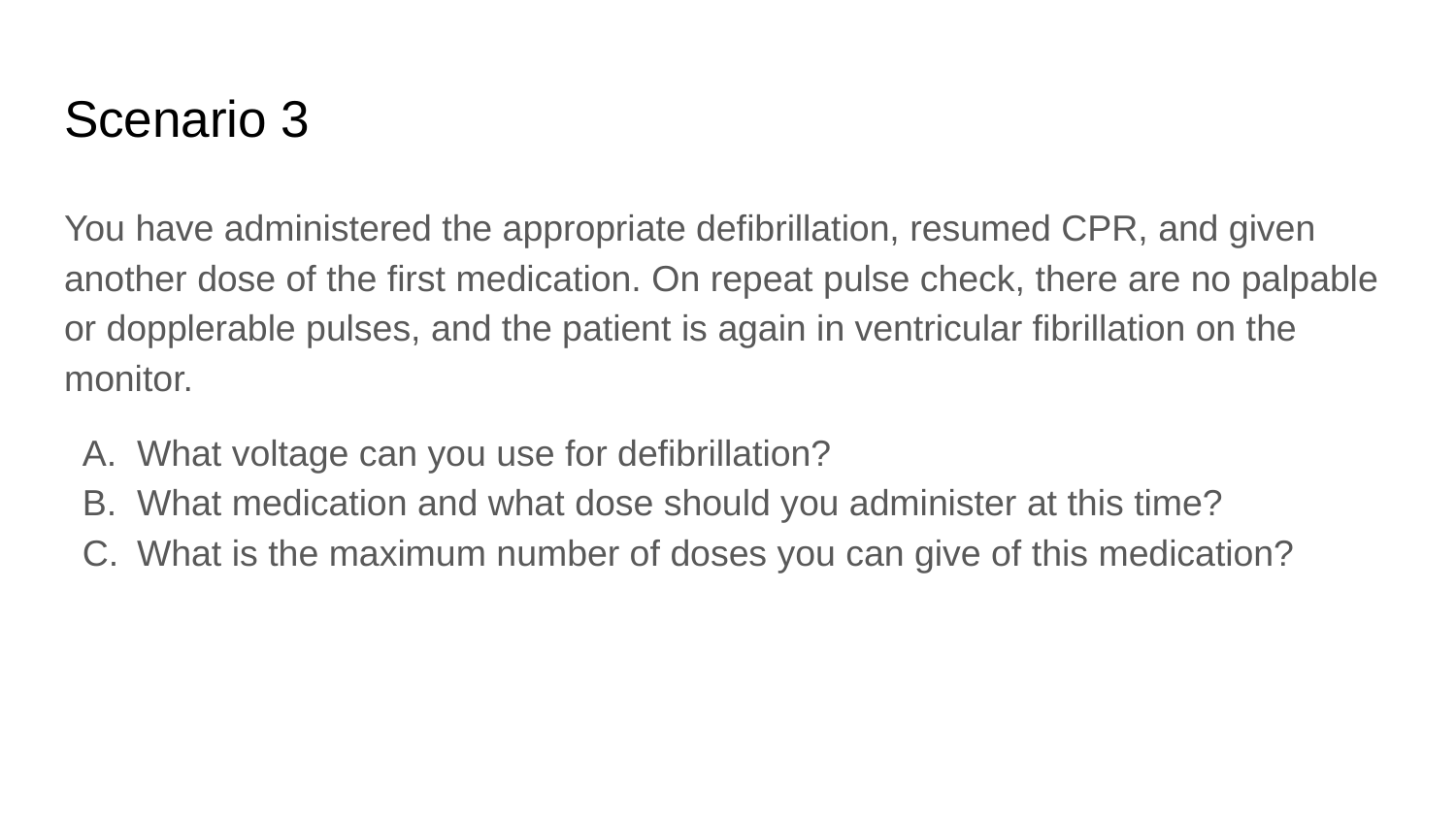

# Scenario 3
You have administered the appropriate defibrillation, resumed CPR, and given another dose of the first medication. On repeat pulse check, there are no palpable or dopplerable pulses, and the patient is again in ventricular fibrillation on the monitor.
What voltage can you use for defibrillation?
What medication and what dose should you administer at this time?
What is the maximum number of doses you can give of this medication?

## Slide 7
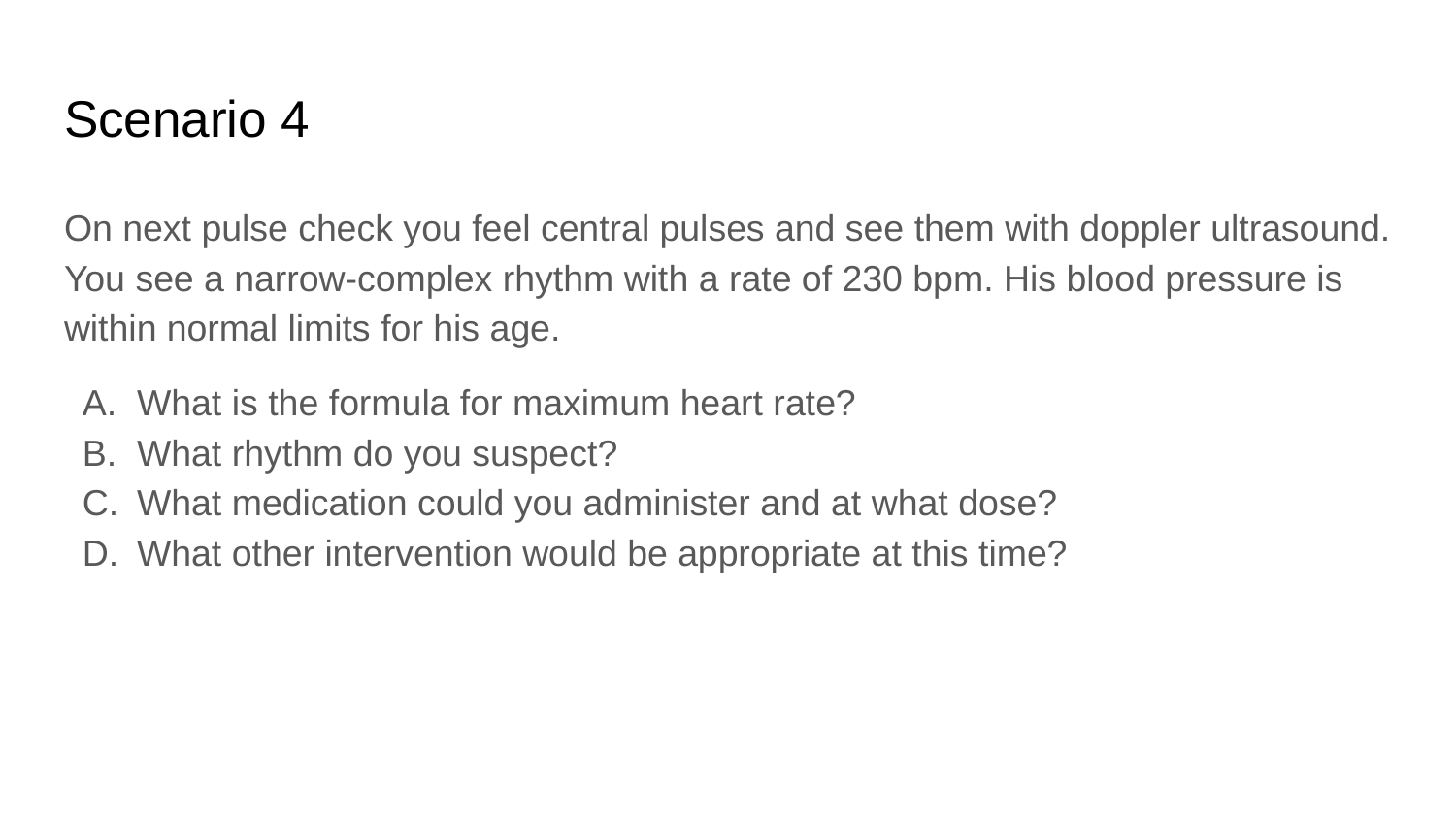

# Scenario 4
On next pulse check you feel central pulses and see them with doppler ultrasound. You see a narrow-complex rhythm with a rate of 230 bpm. His blood pressure is within normal limits for his age.
What is the formula for maximum heart rate?
What rhythm do you suspect?
What medication could you administer and at what dose?
What other intervention would be appropriate at this time?

## Slide 8
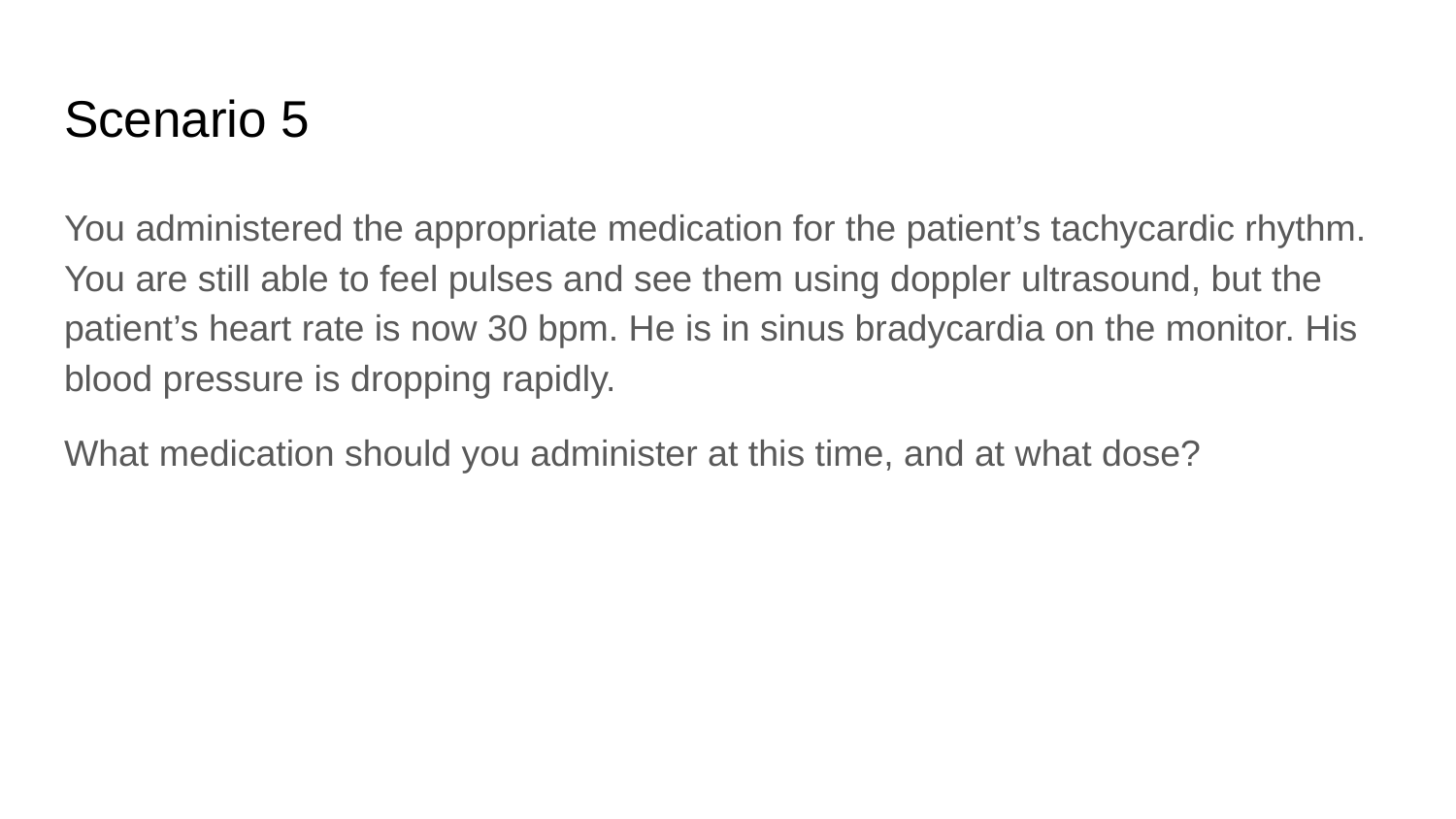

# Scenario 5
You administered the appropriate medication for the patient’s tachycardic rhythm. You are still able to feel pulses and see them using doppler ultrasound, but the patient’s heart rate is now 30 bpm. He is in sinus bradycardia on the monitor. His blood pressure is dropping rapidly.
What medication should you administer at this time, and at what dose?

## Slide 9
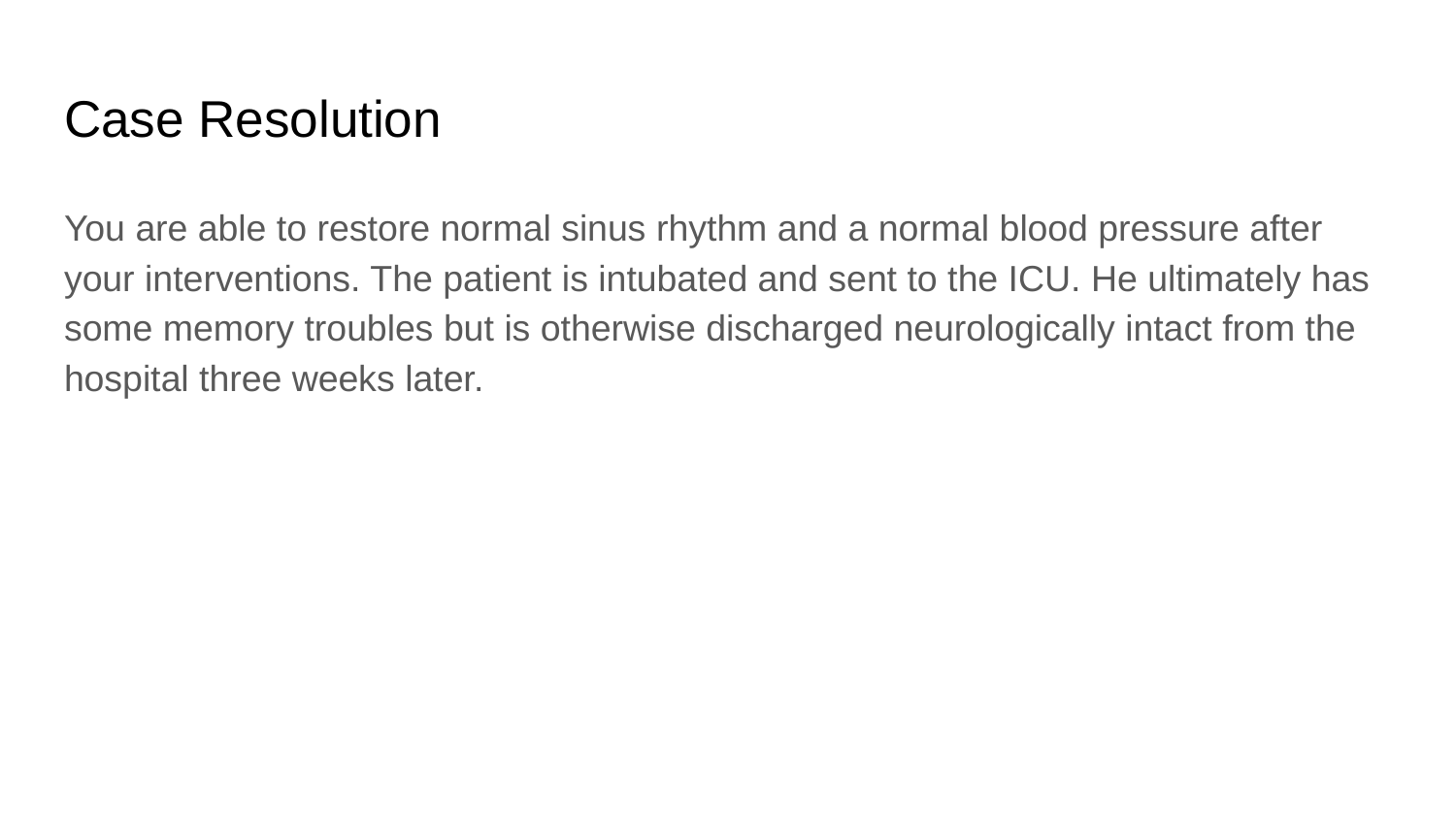

# Case Resolution
You are able to restore normal sinus rhythm and a normal blood pressure after your interventions. The patient is intubated and sent to the ICU. He ultimately has some memory troubles but is otherwise discharged neurologically intact from the hospital three weeks later.

## Slide 10
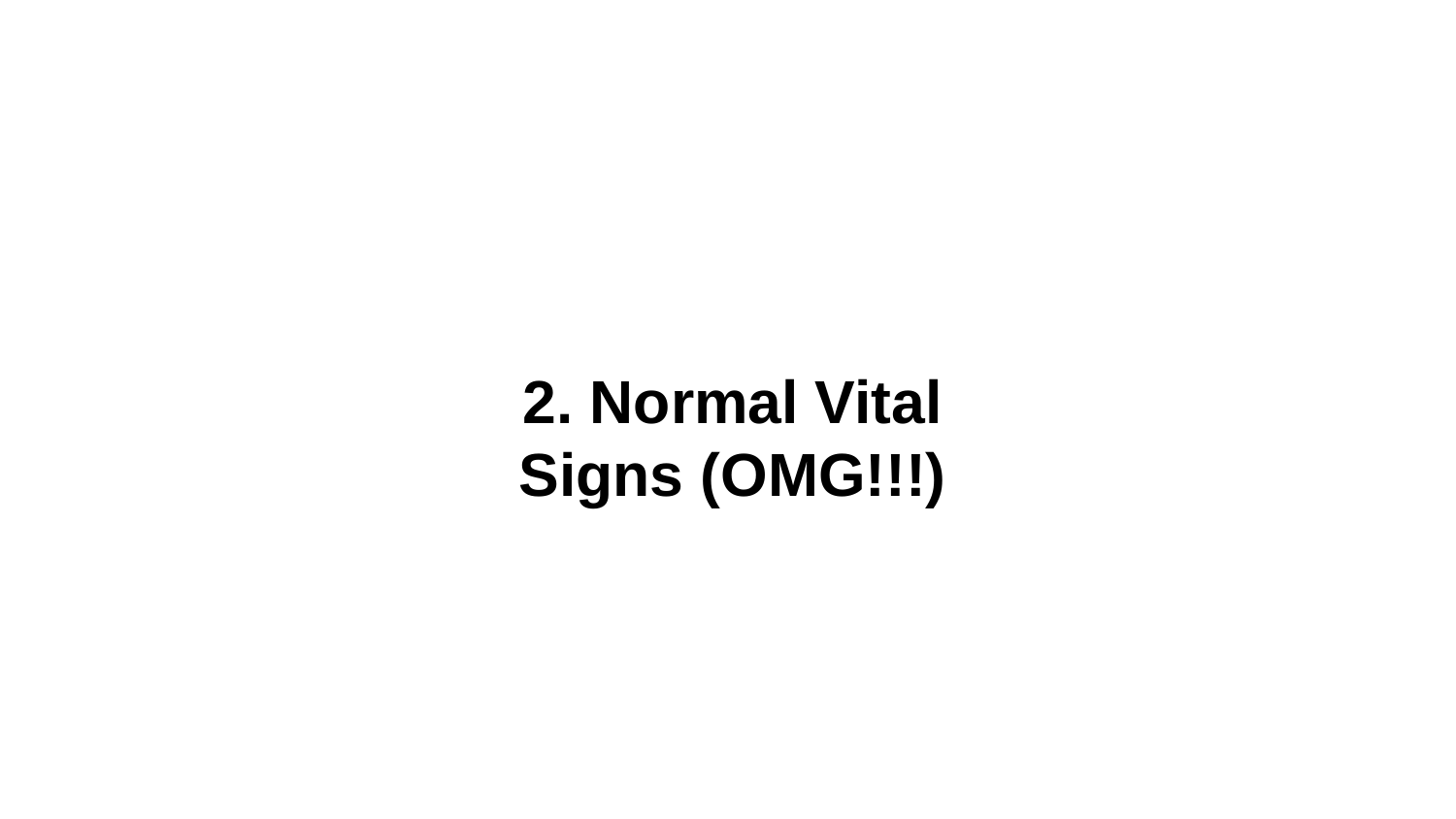

2. Normal Vital Signs (OMG!!!)

## Slide 11
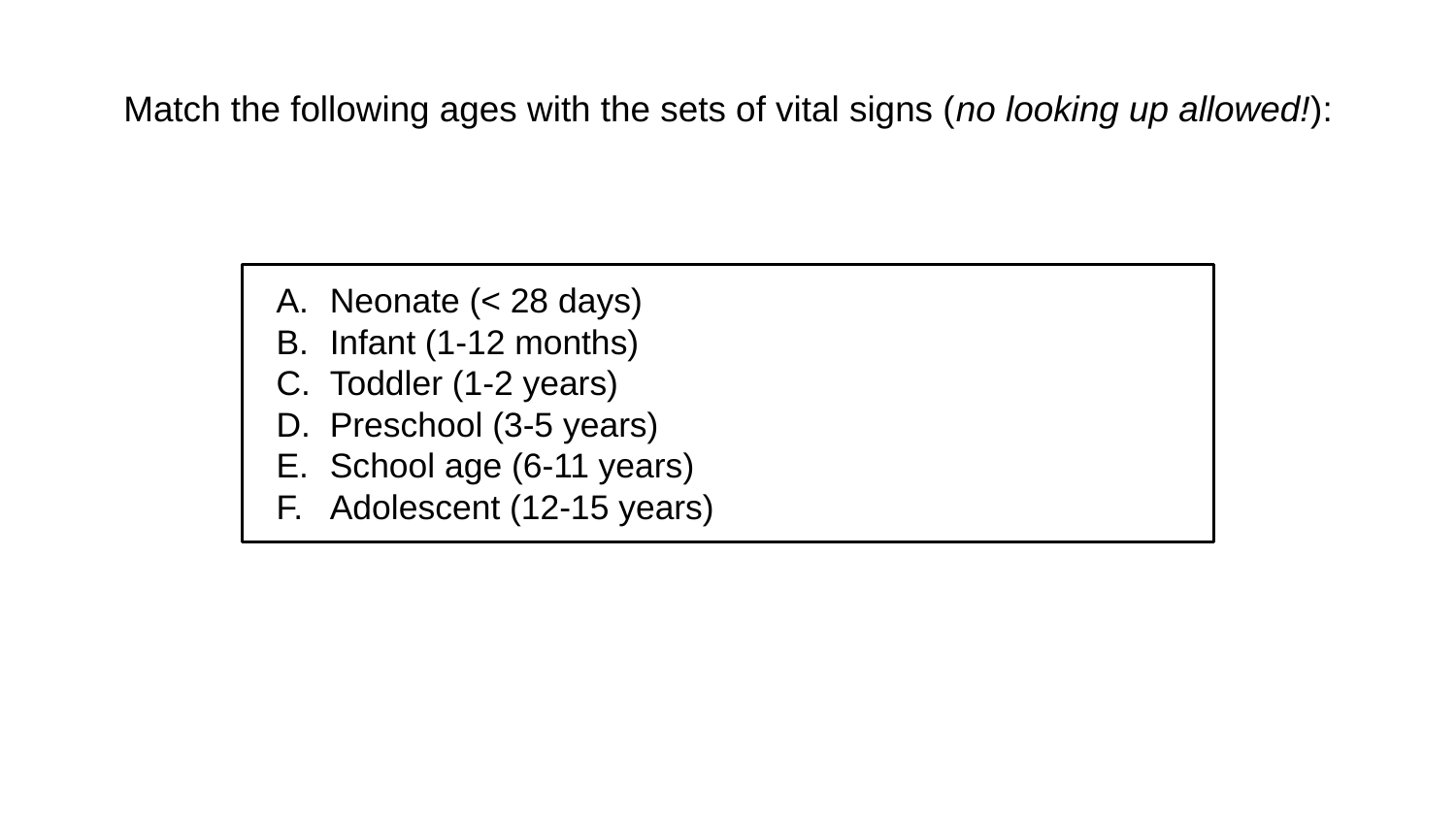

# Match the following ages with the sets of vital signs (no looking up allowed!):
Neonate (< 28 days)
Infant (1-12 months)
Toddler (1-2 years)
Preschool (3-5 years)
School age (6-11 years)
Adolescent (12-15 years)

## Slide 12
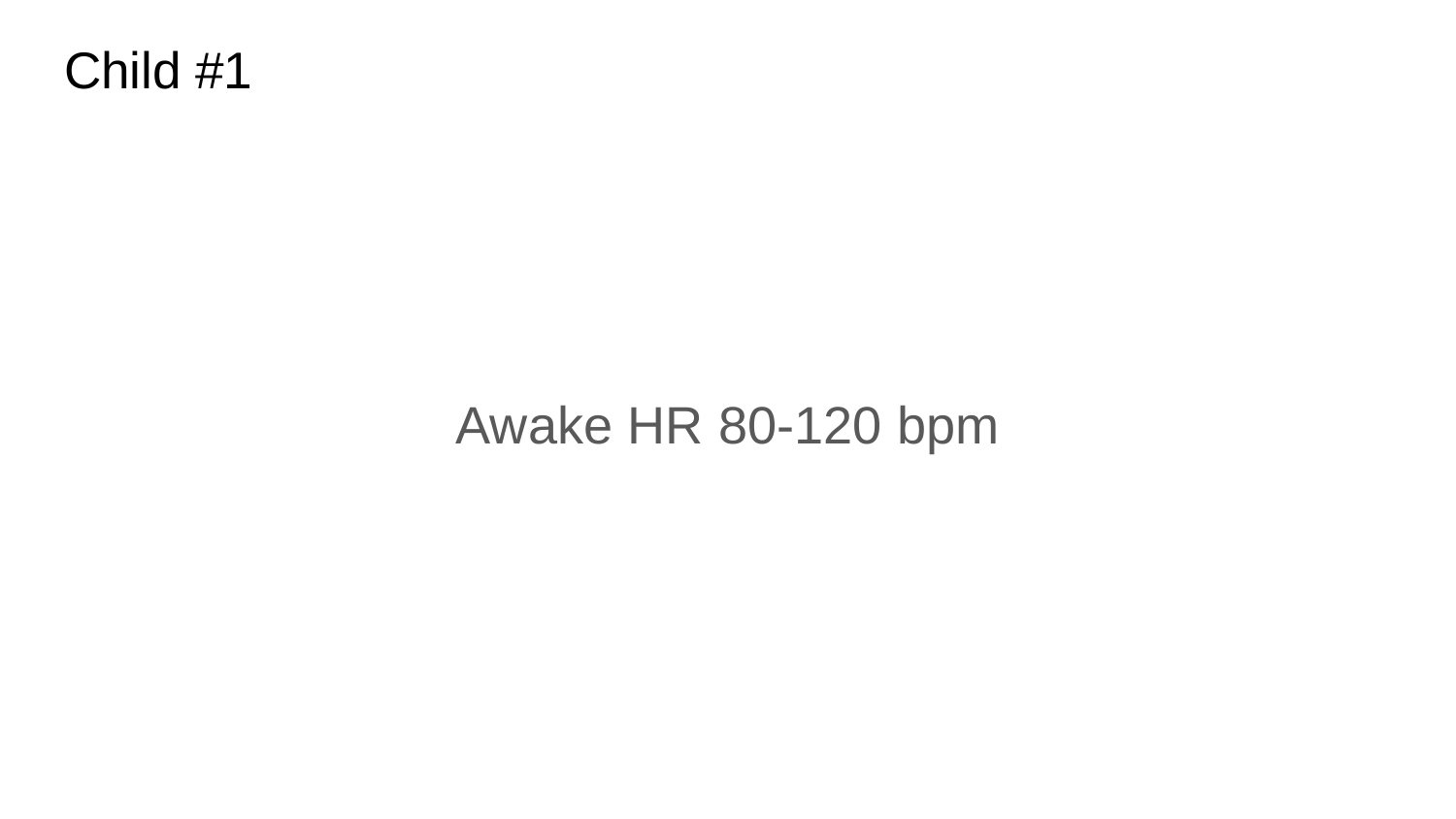

# Child #1
Awake HR 80-120 bpm

## Slide 13
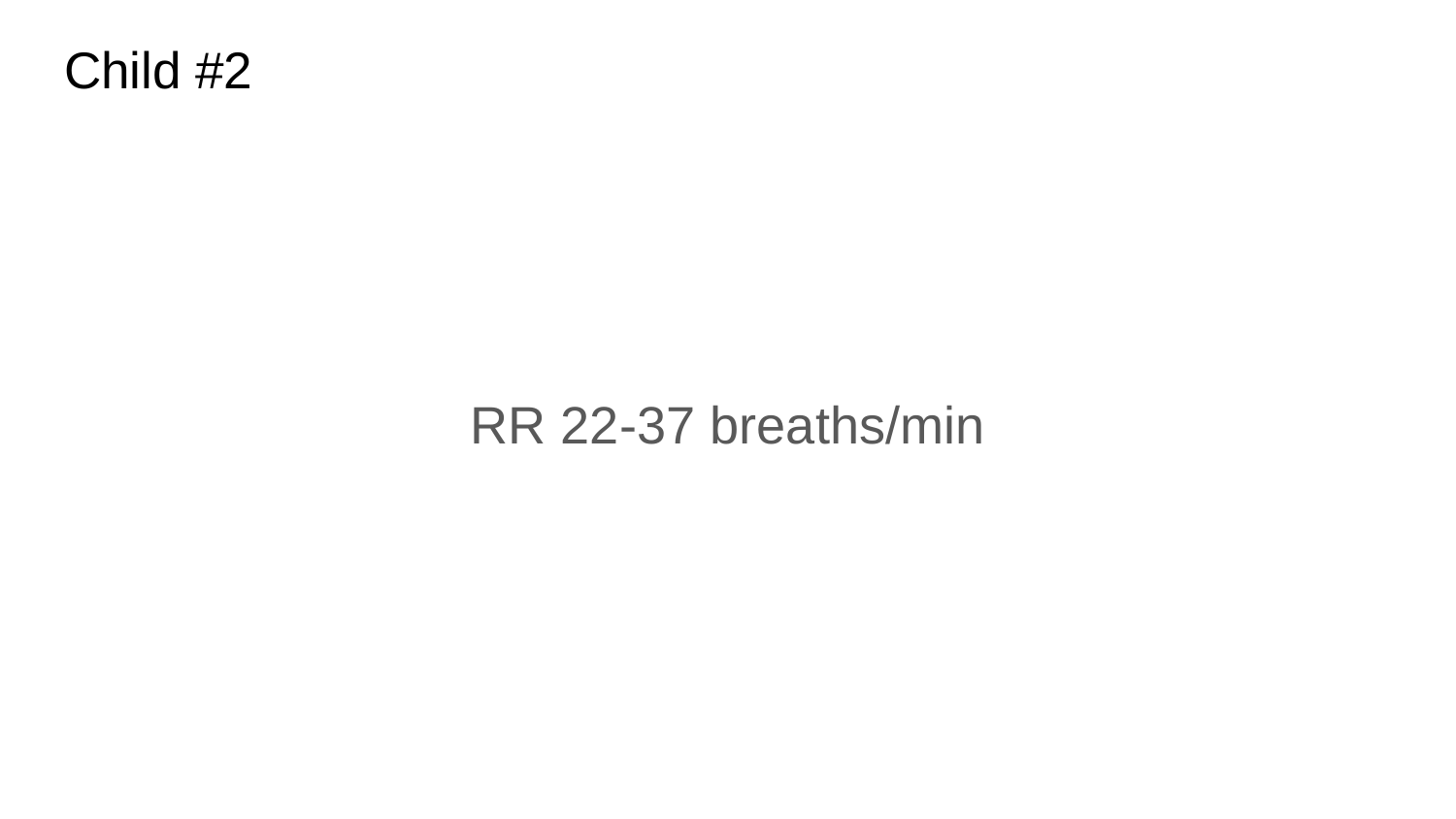

# Child #2
RR 22-37 breaths/min

## Slide 14
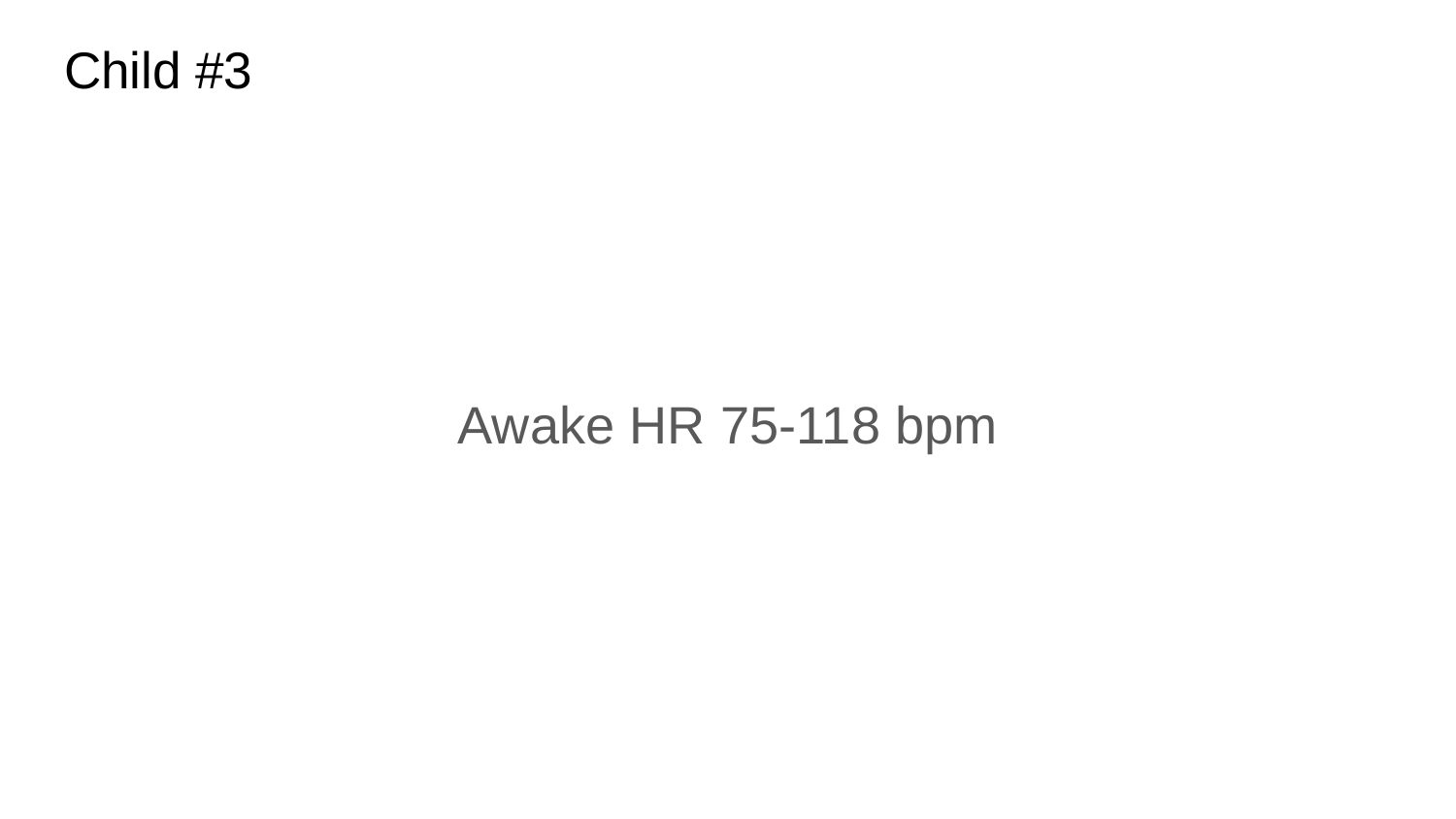

# Child #3
Awake HR 75-118 bpm

## Slide 15
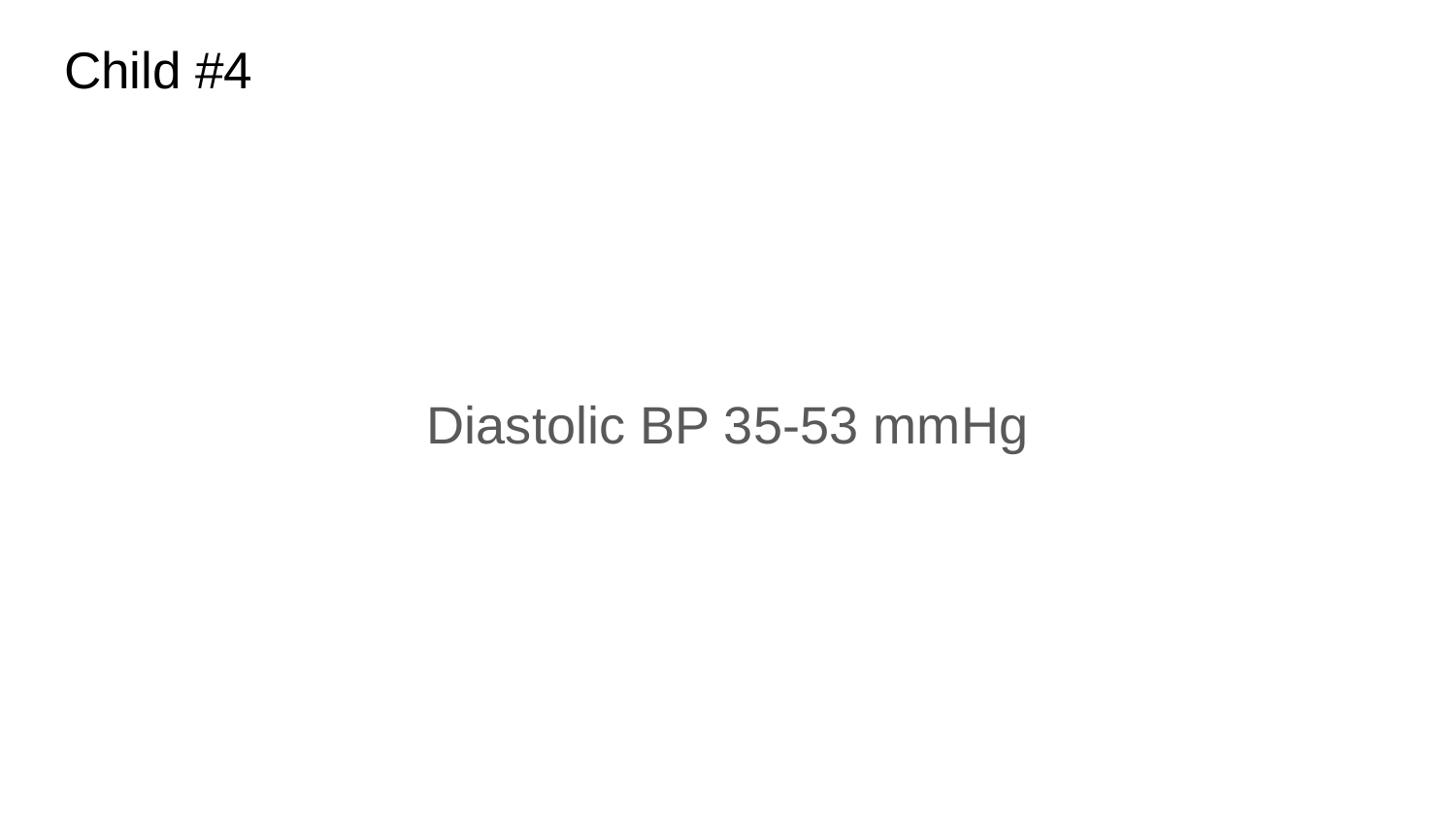

# Child #4
Diastolic BP 35-53 mmHg

## Slide 16
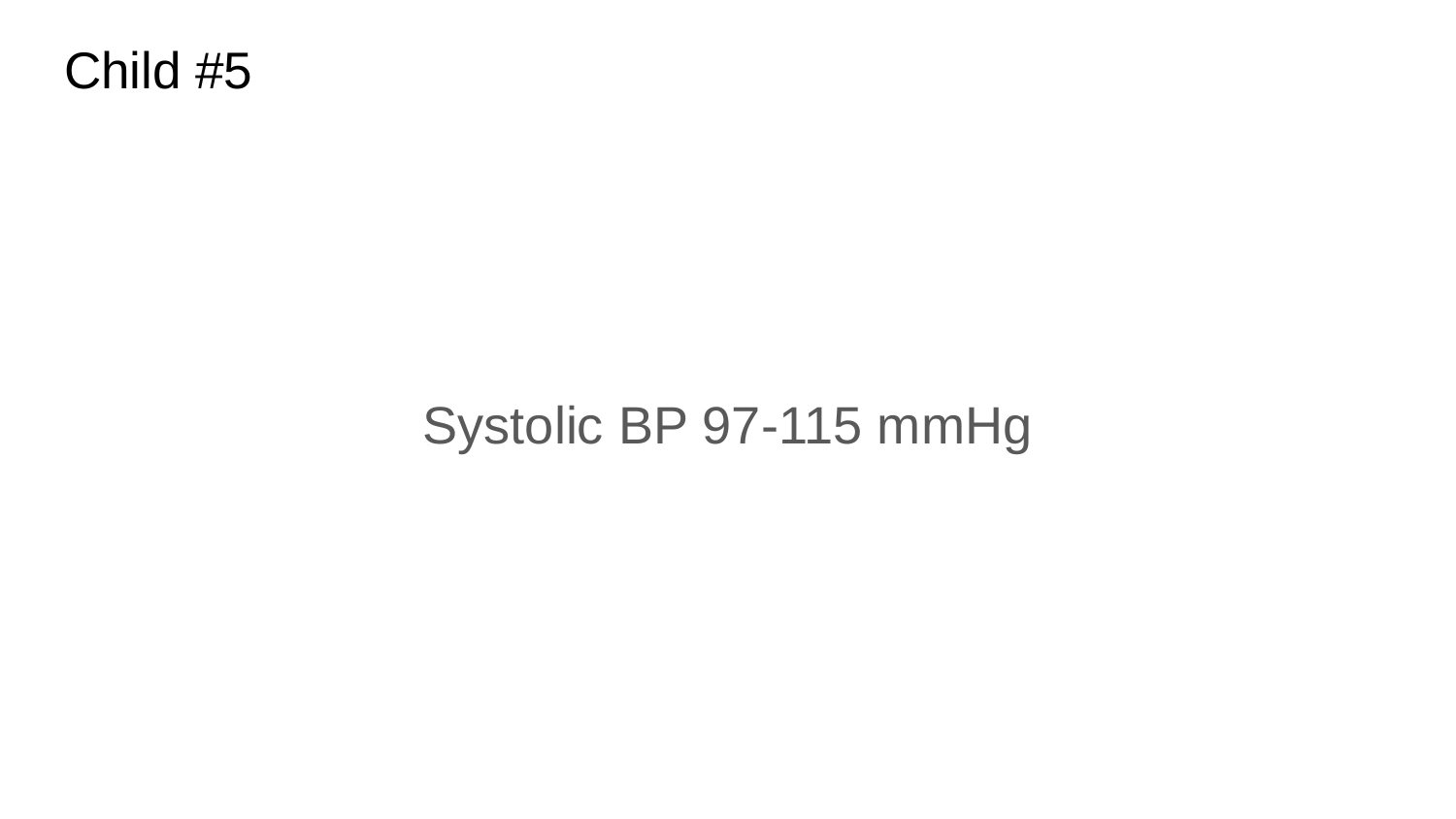

# Child #5
Systolic BP 97-115 mmHg

## Slide 17
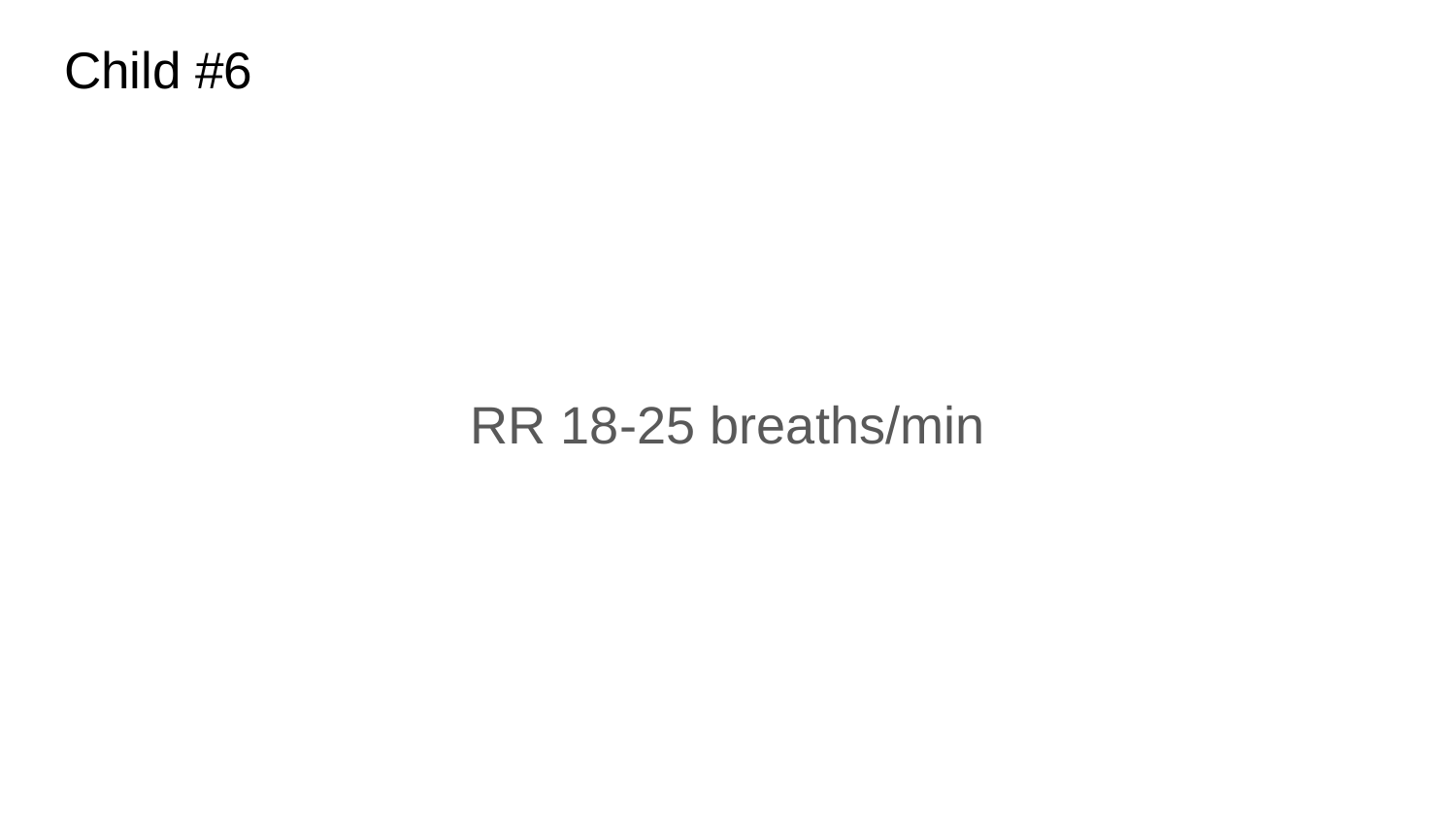

# Child #6
RR 18-25 breaths/min

## Slide 18
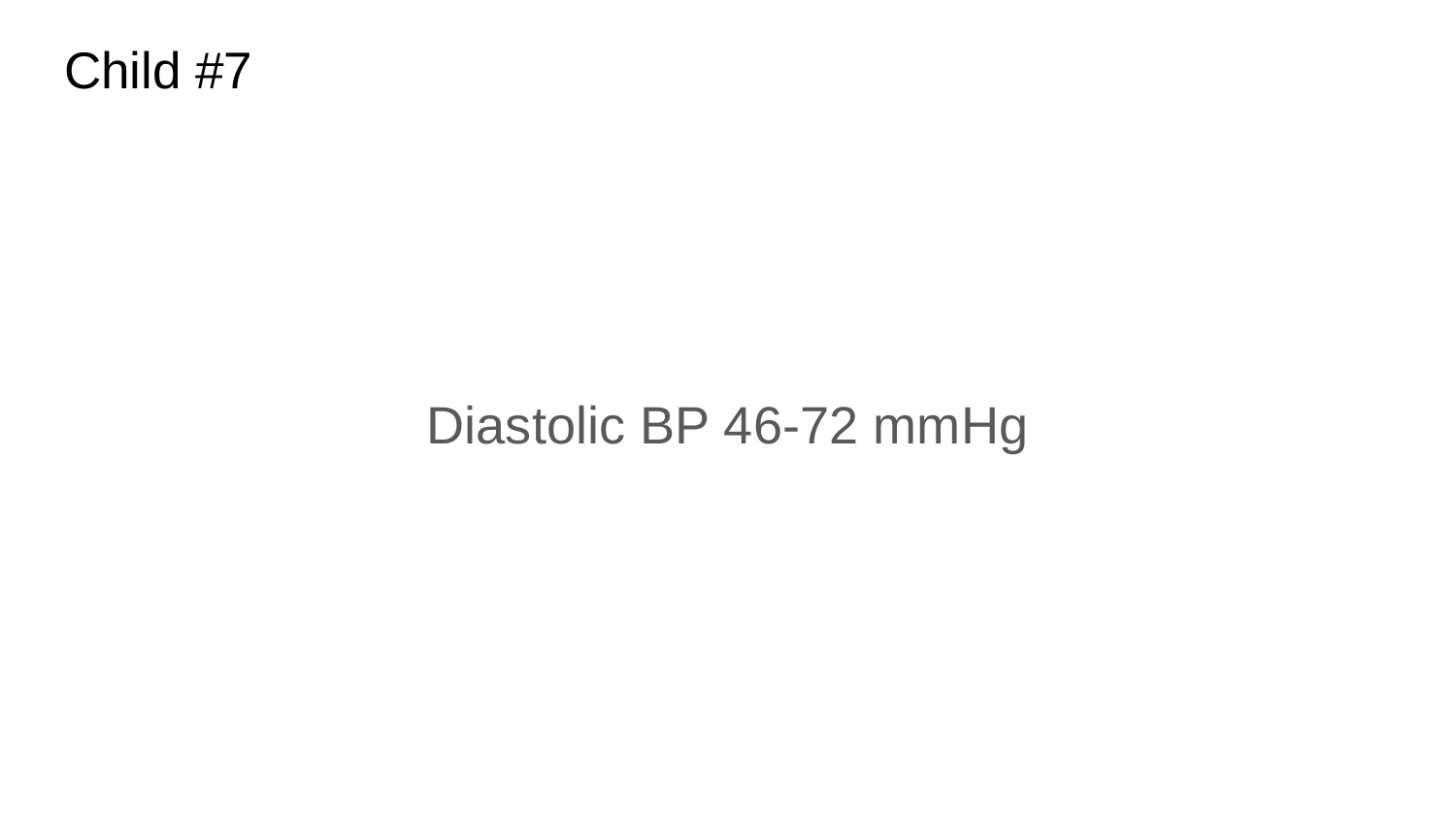

# Child #7
Diastolic BP 46-72 mmHg

## Slide 19
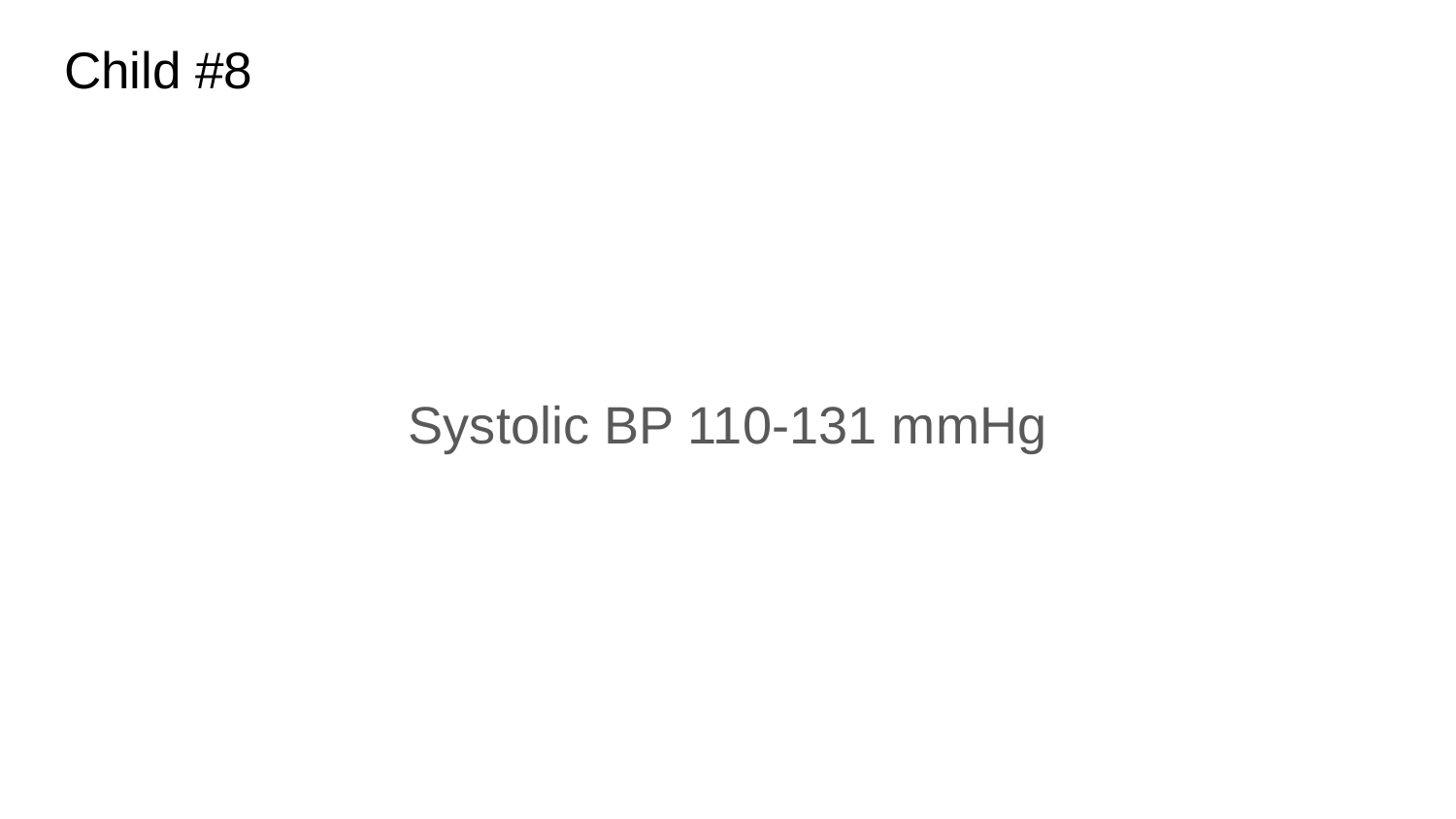

# Child #8
Systolic BP 110-131 mmHg

## Slide 20
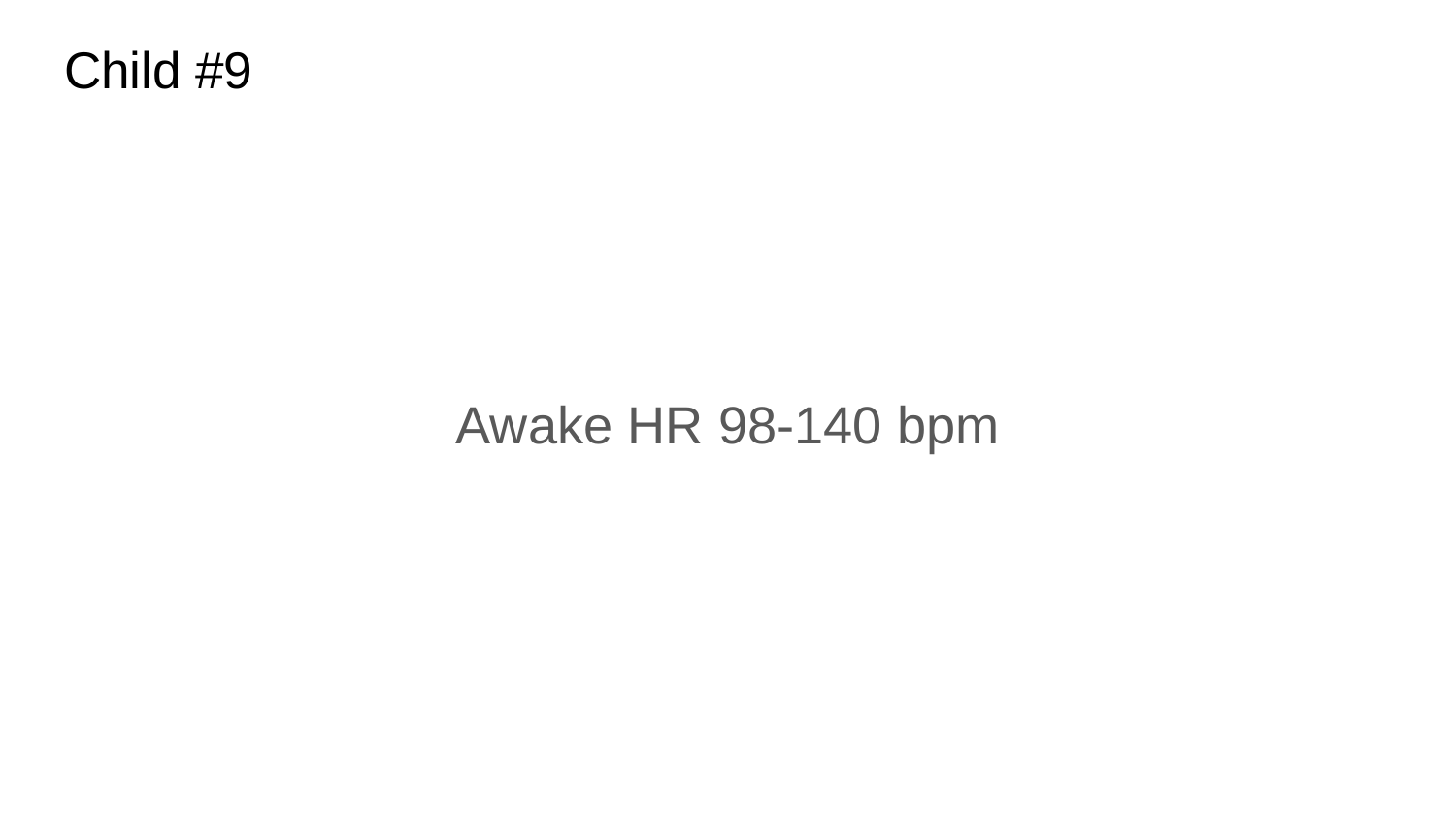

# Child #9
Awake HR 98-140 bpm

## Slide 21
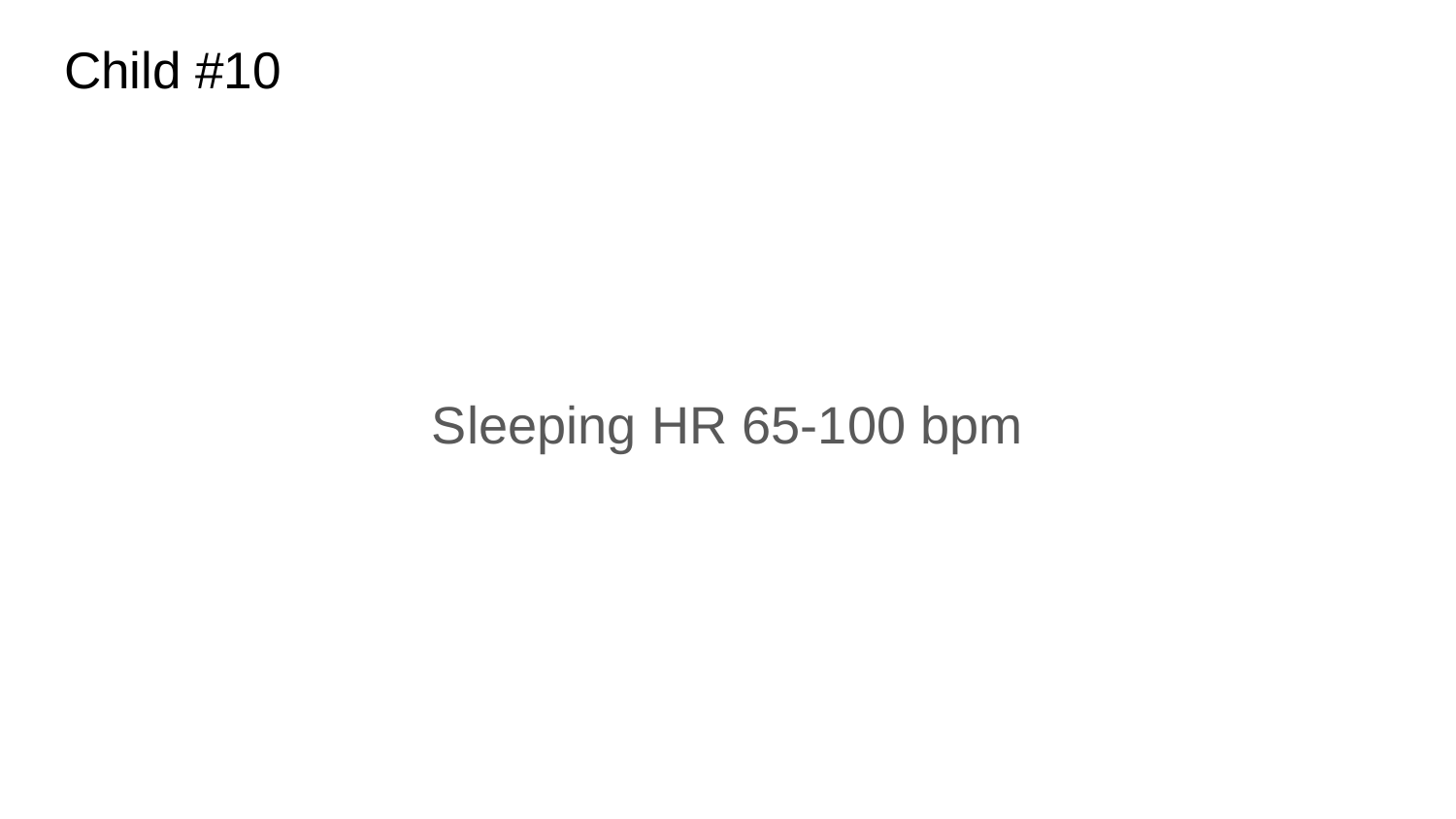

# Child #10
Sleeping HR 65-100 bpm

## Slide 22
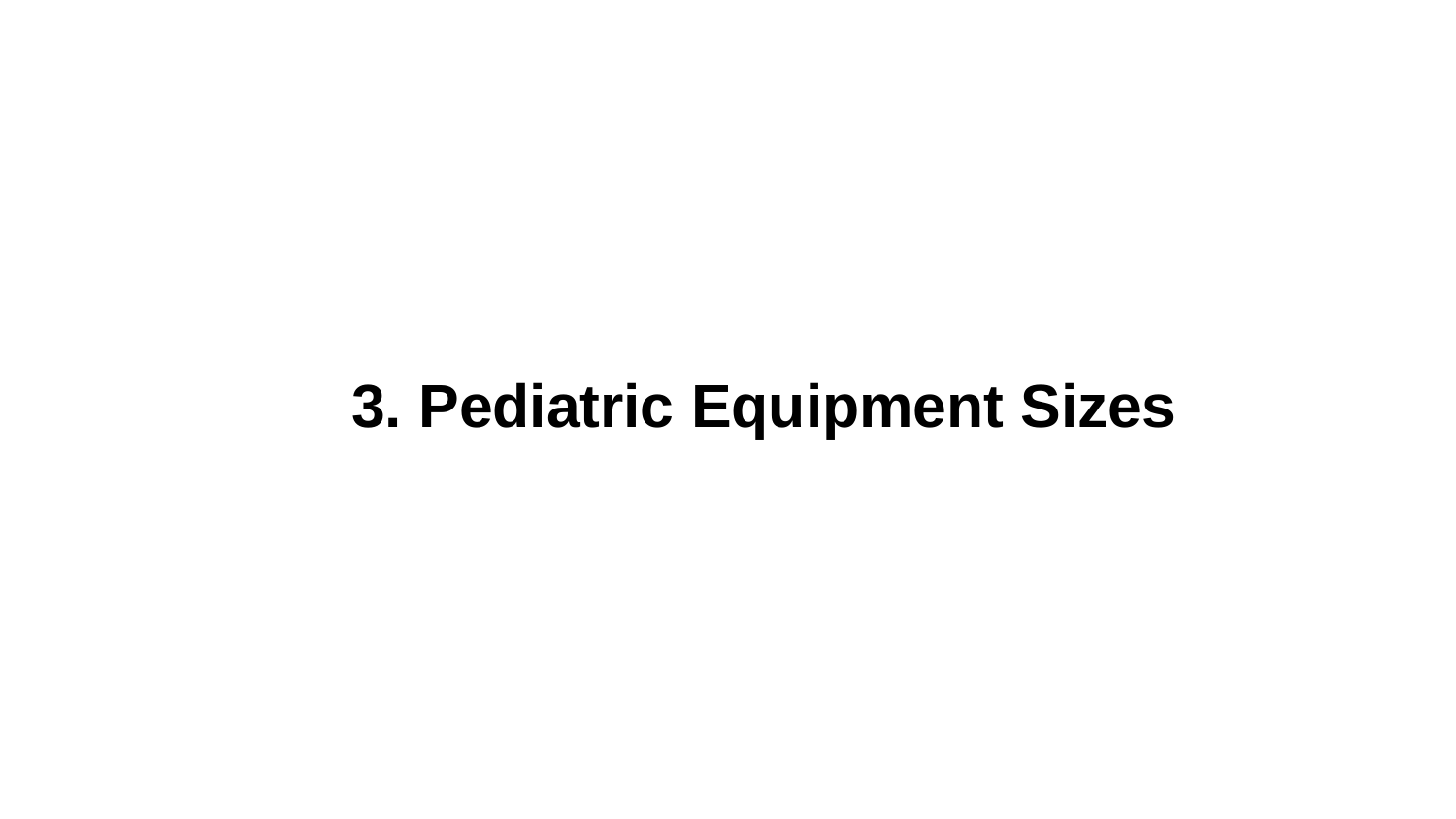

3. Pediatric Equipment Sizes

## Slide 23
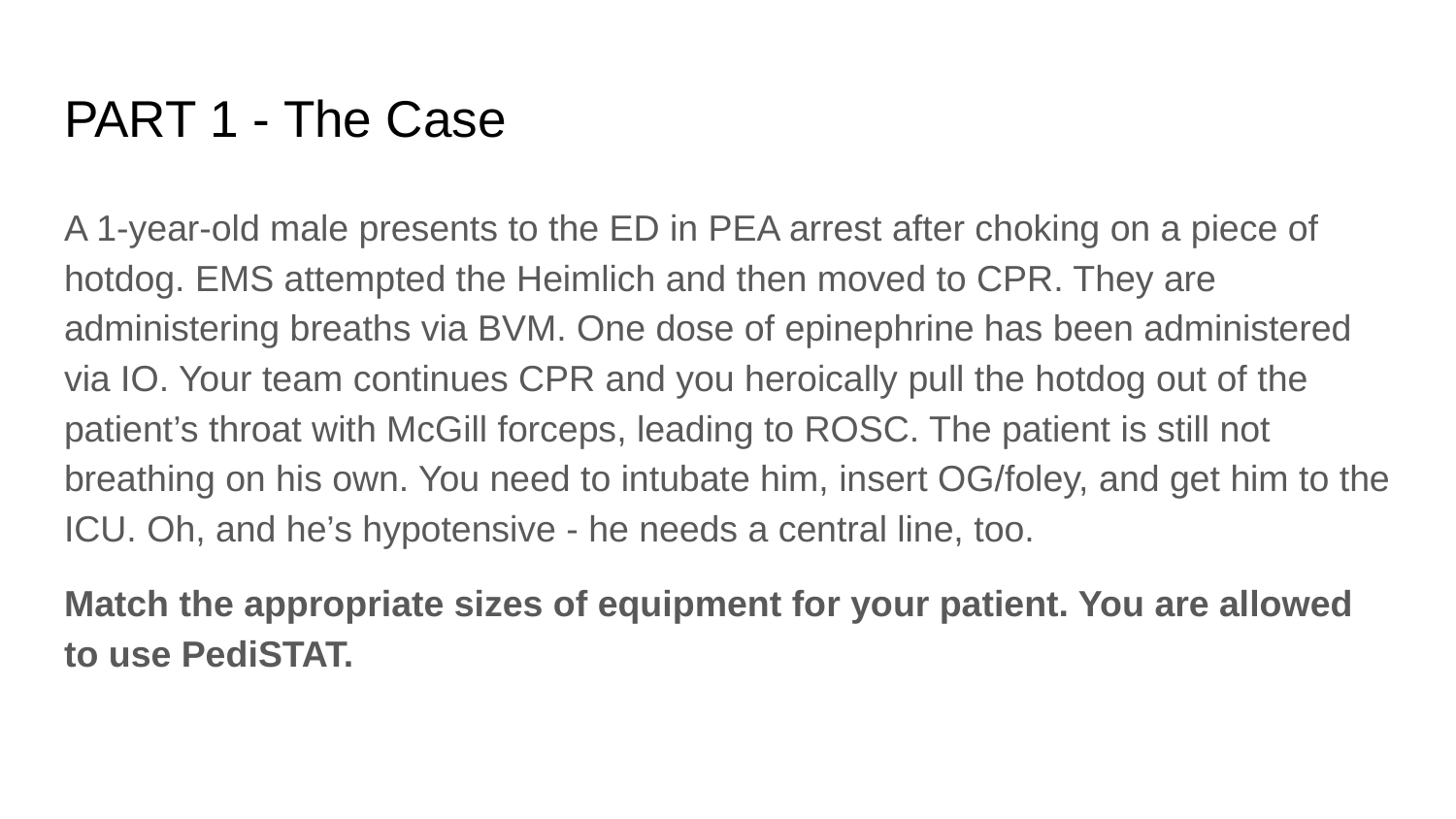

# PART 1 - The Case
A 1-year-old male presents to the ED in PEA arrest after choking on a piece of hotdog. EMS attempted the Heimlich and then moved to CPR. They are administering breaths via BVM. One dose of epinephrine has been administered via IO. Your team continues CPR and you heroically pull the hotdog out of the patient’s throat with McGill forceps, leading to ROSC. The patient is still not breathing on his own. You need to intubate him, insert OG/foley, and get him to the ICU. Oh, and he’s hypotensive - he needs a central line, too.
Match the appropriate sizes of equipment for your patient. You are allowed to use PediSTAT.

## Slide 24
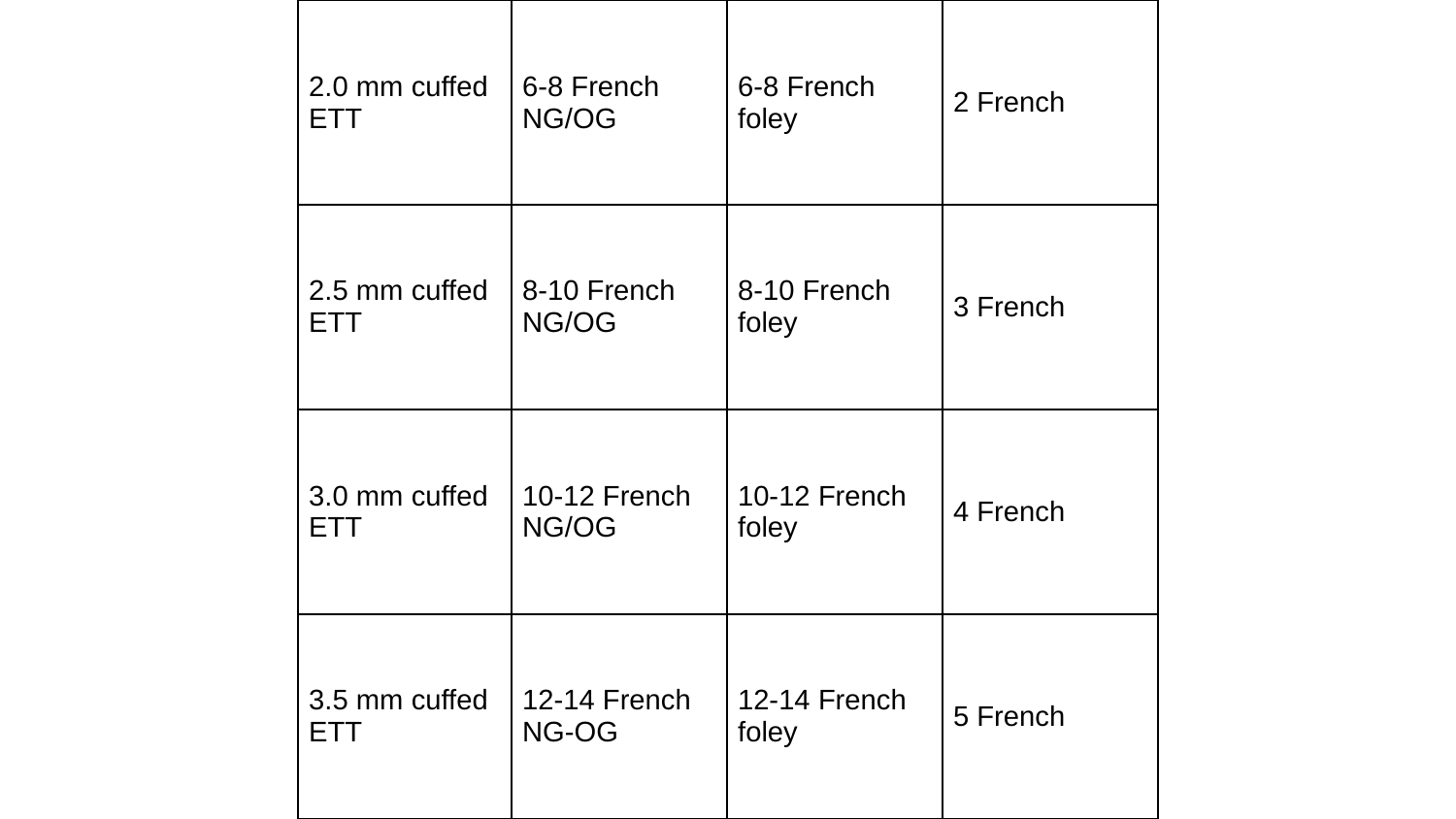

| 2.0 mm cuffed ETT | 6-8 French NG/OG | 6-8 French foley | 2 French |
| --- | --- | --- | --- |
| 2.5 mm cuffed ETT | 8-10 French NG/OG | 8-10 French foley | 3 French |
| 3.0 mm cuffed ETT | 10-12 French NG/OG | 10-12 French foley | 4 French |
| 3.5 mm cuffed ETT | 12-14 French NG-OG | 12-14 French foley | 5 French |

## Slide 25
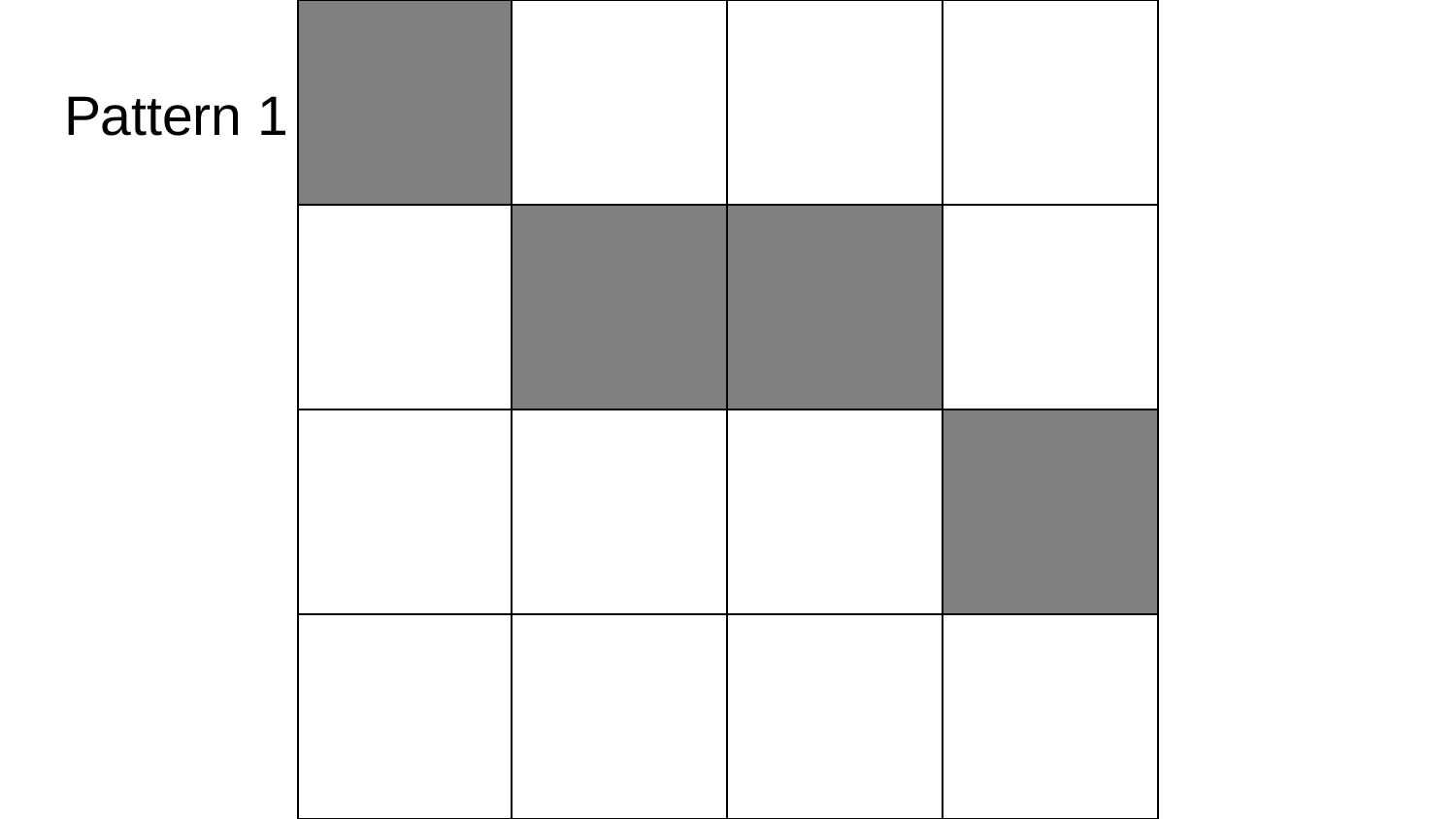

| | | | |
| --- | --- | --- | --- |
| | | | |
| | | | |
| | | | |
# Pattern 1

## Slide 26
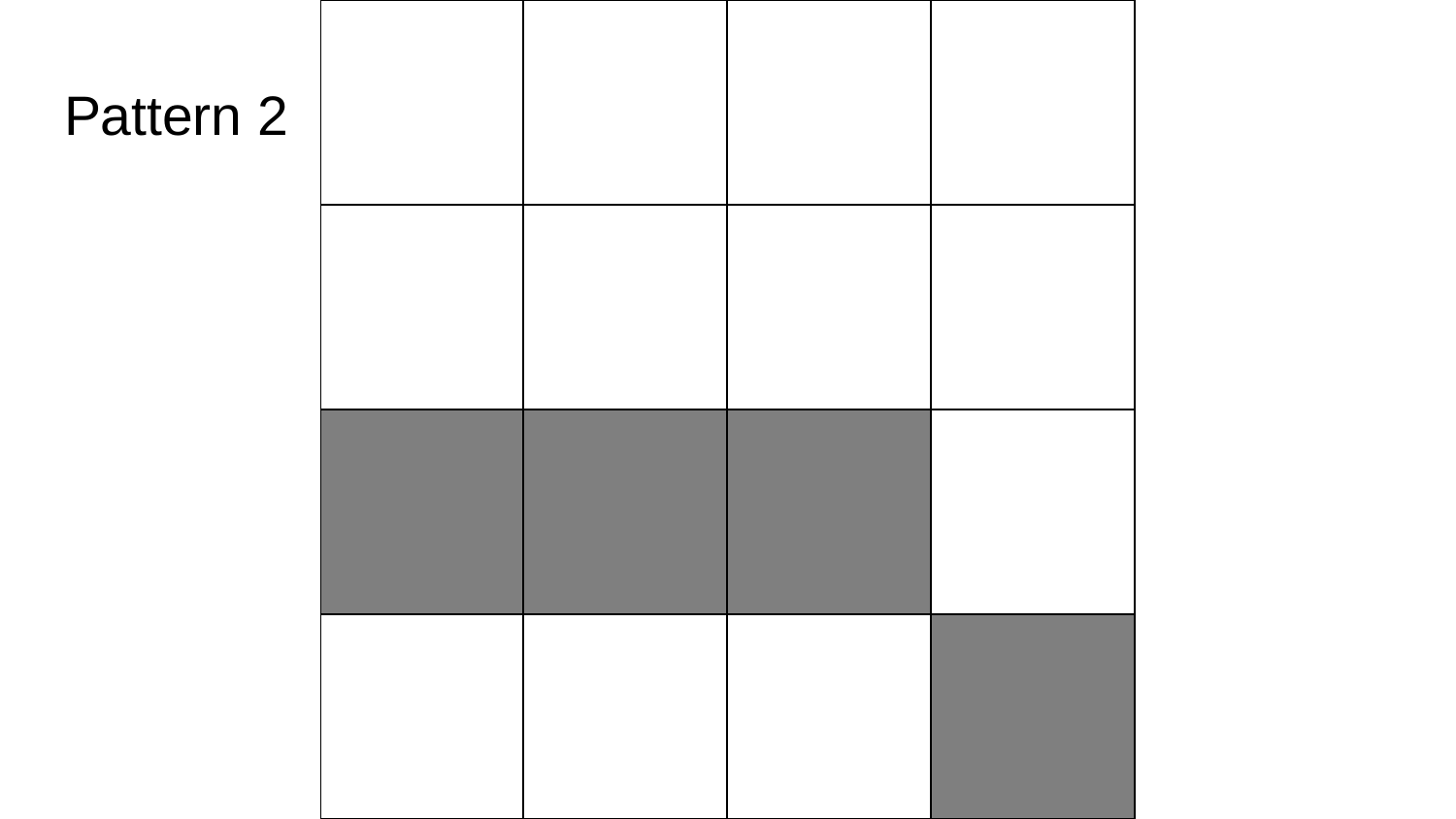

| | | | |
| --- | --- | --- | --- |
| | | | |
| | | | |
| | | | |
# Pattern 2

## Slide 27
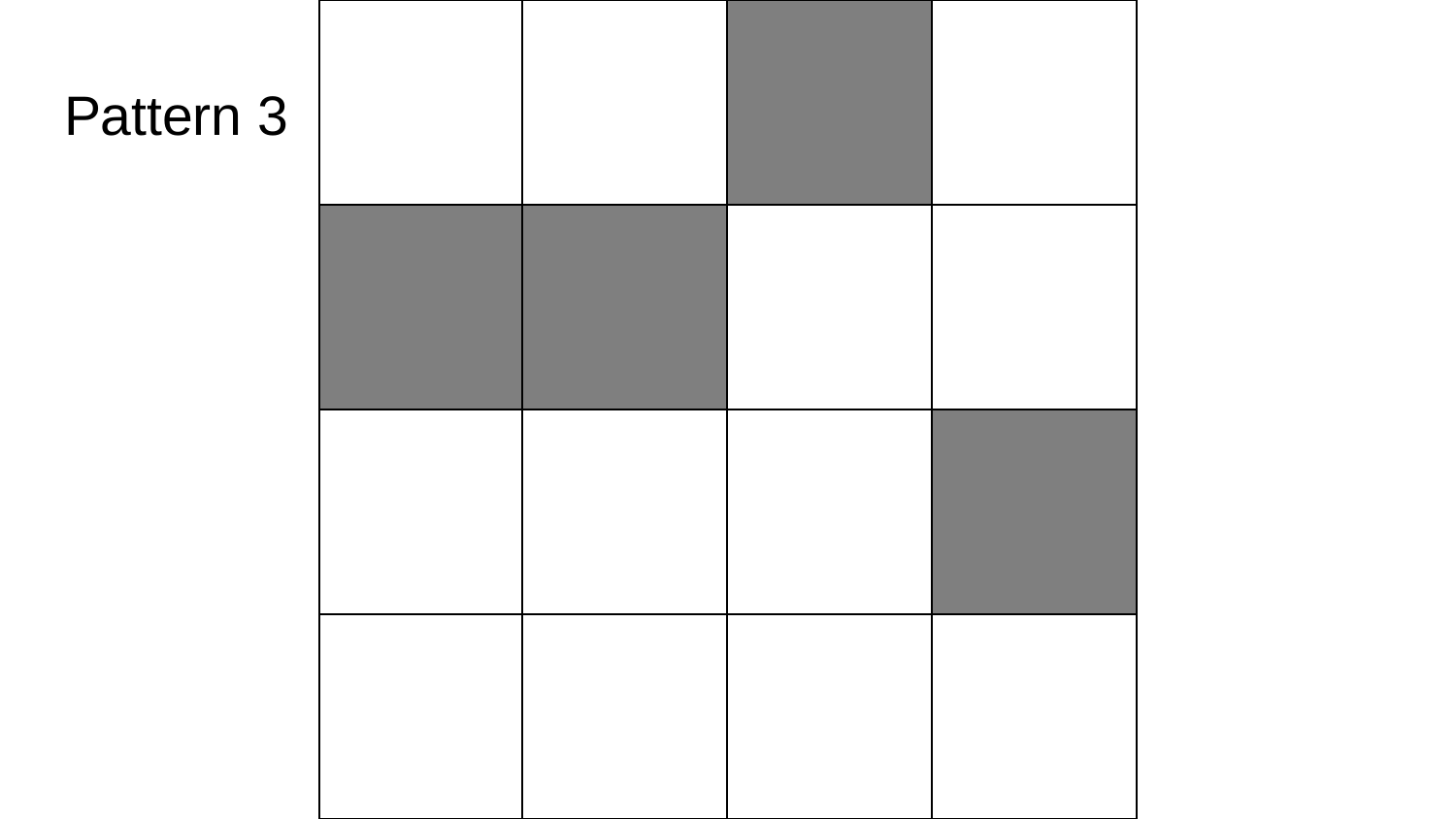

| | | | |
| --- | --- | --- | --- |
| | | | |
| | | | |
| | | | |
# Pattern 3

## Slide 28
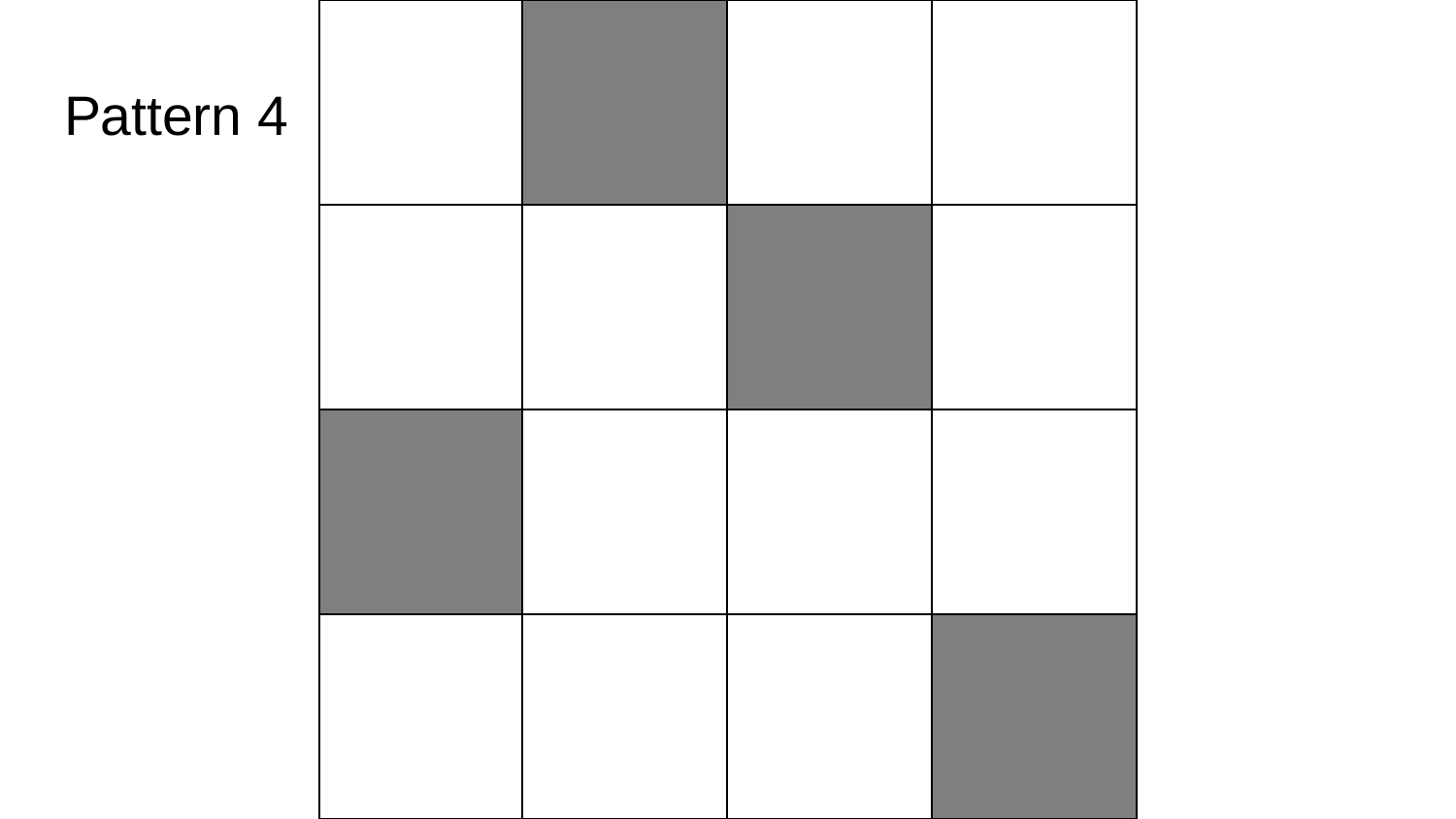

| | | | |
| --- | --- | --- | --- |
| | | | |
| | | | |
| | | | |
# Pattern 4

## Slide 29
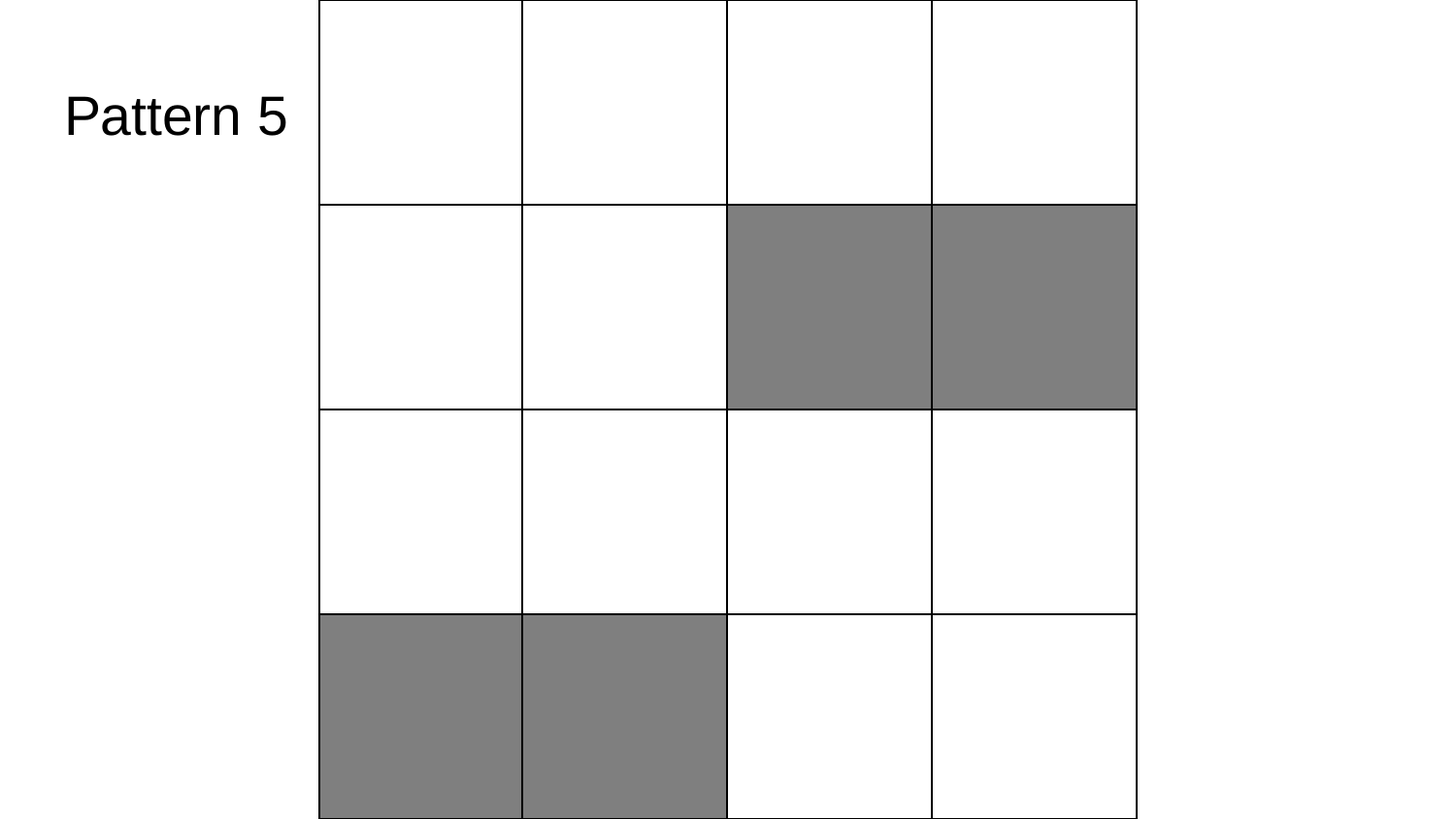

| | | | |
| --- | --- | --- | --- |
| | | | |
| | | | |
| | | | |
# Pattern 5

## Slide 30
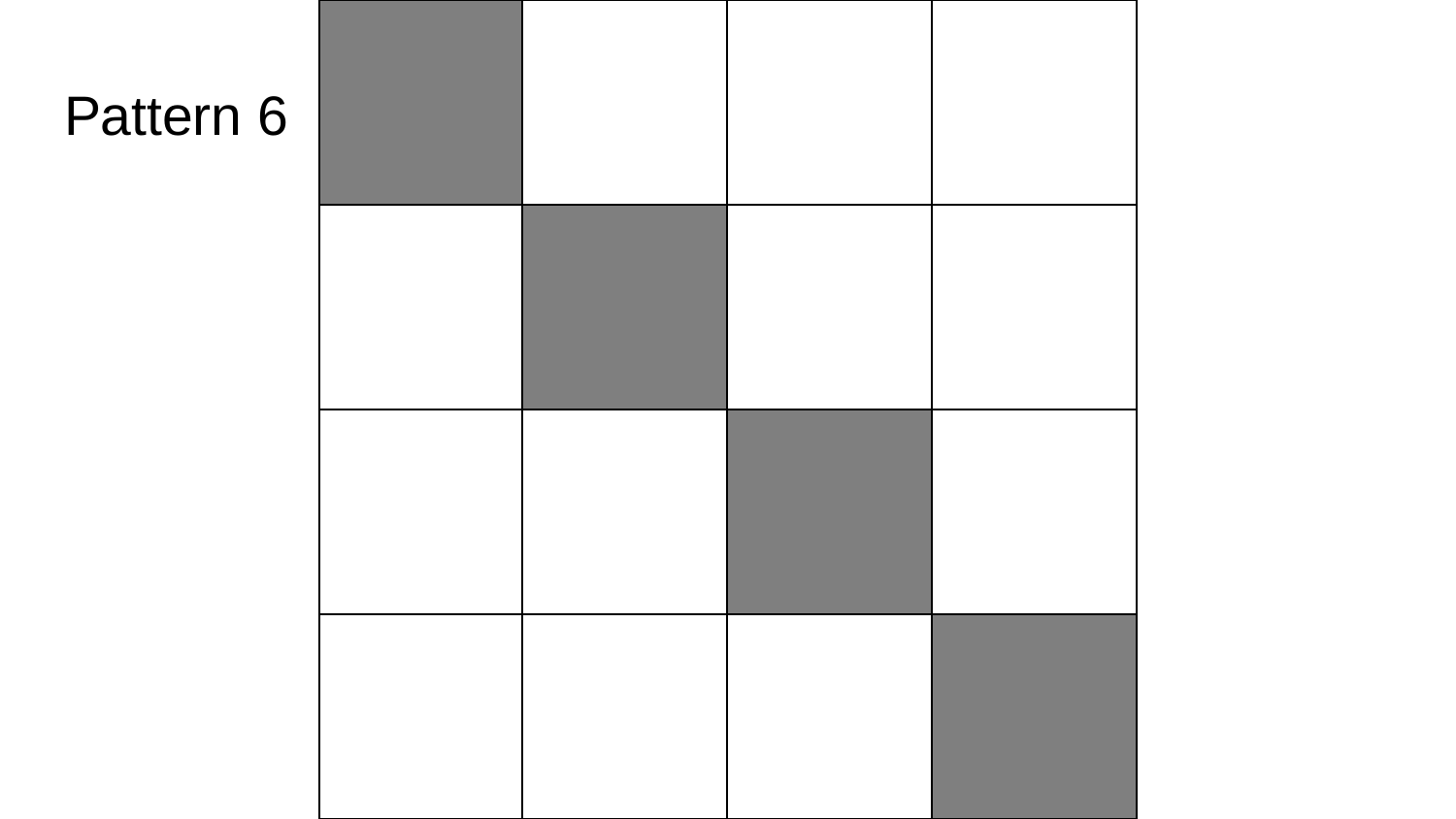

| | | | |
| --- | --- | --- | --- |
| | | | |
| | | | |
| | | | |
# Pattern 6

## Slide 31
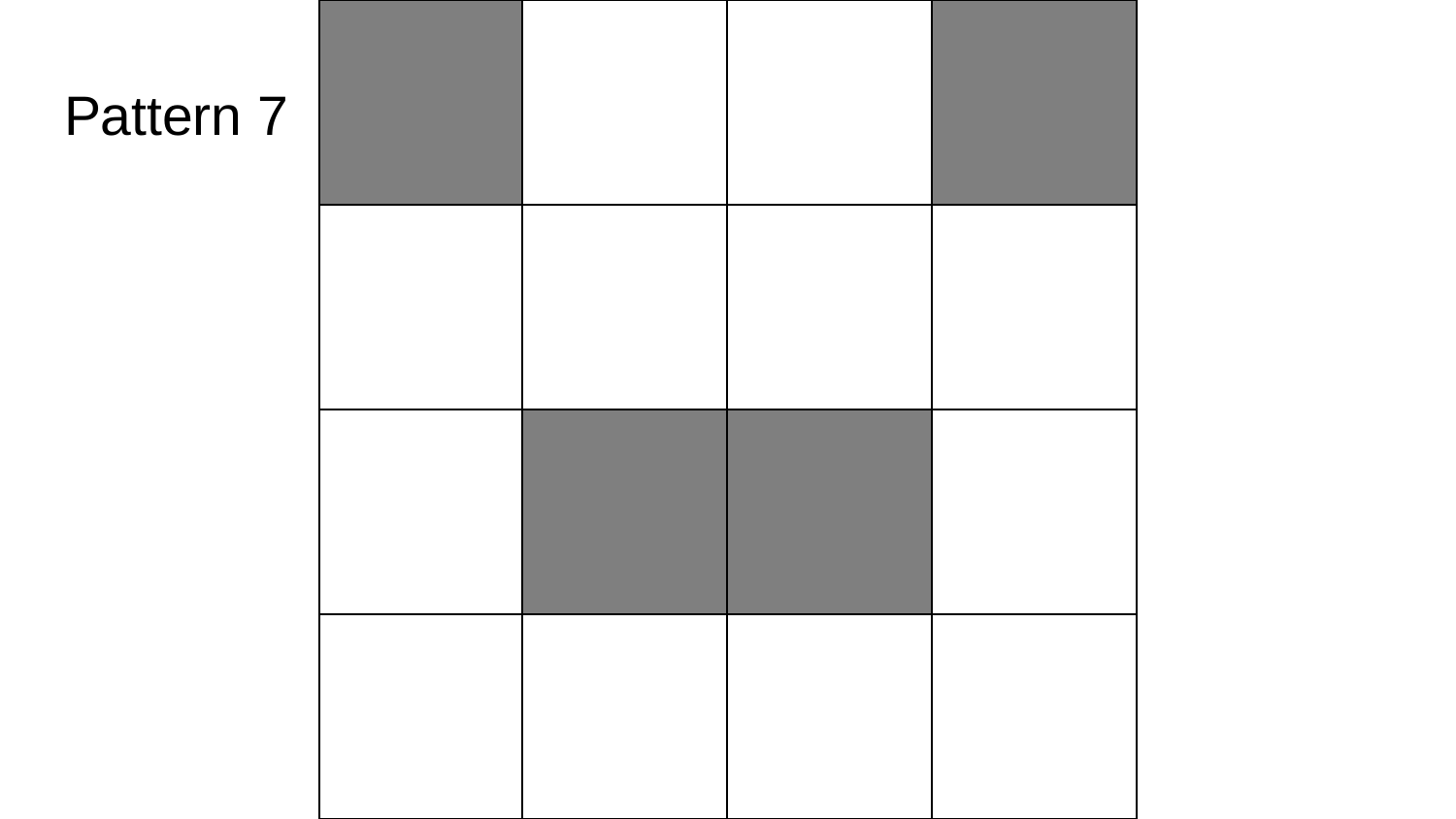

| | | | |
| --- | --- | --- | --- |
| | | | |
| | | | |
| | | | |
# Pattern 7

## Slide 32
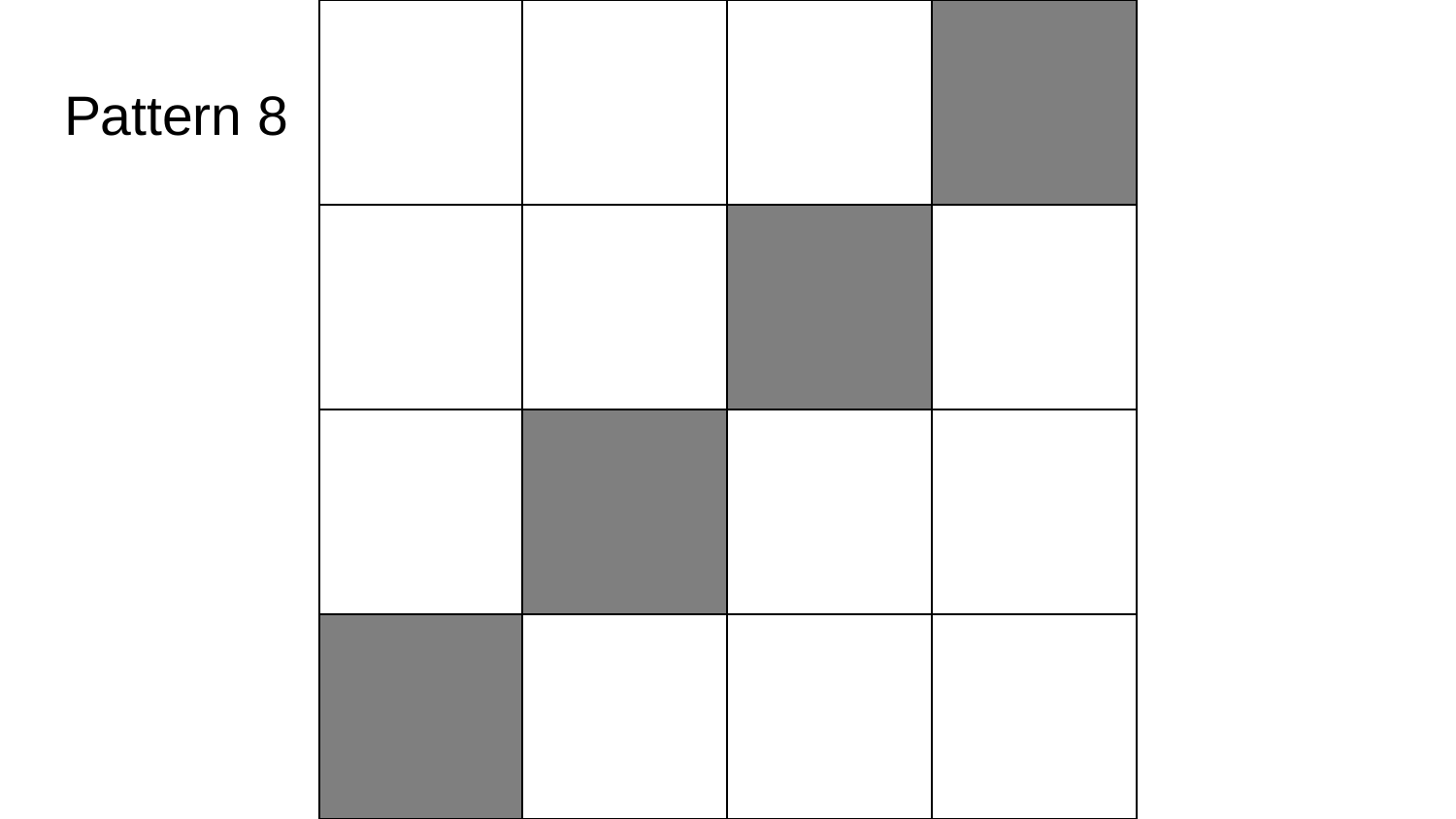

| | | | |
| --- | --- | --- | --- |
| | | | |
| | | | |
| | | | |
# Pattern 8

## Slide 33
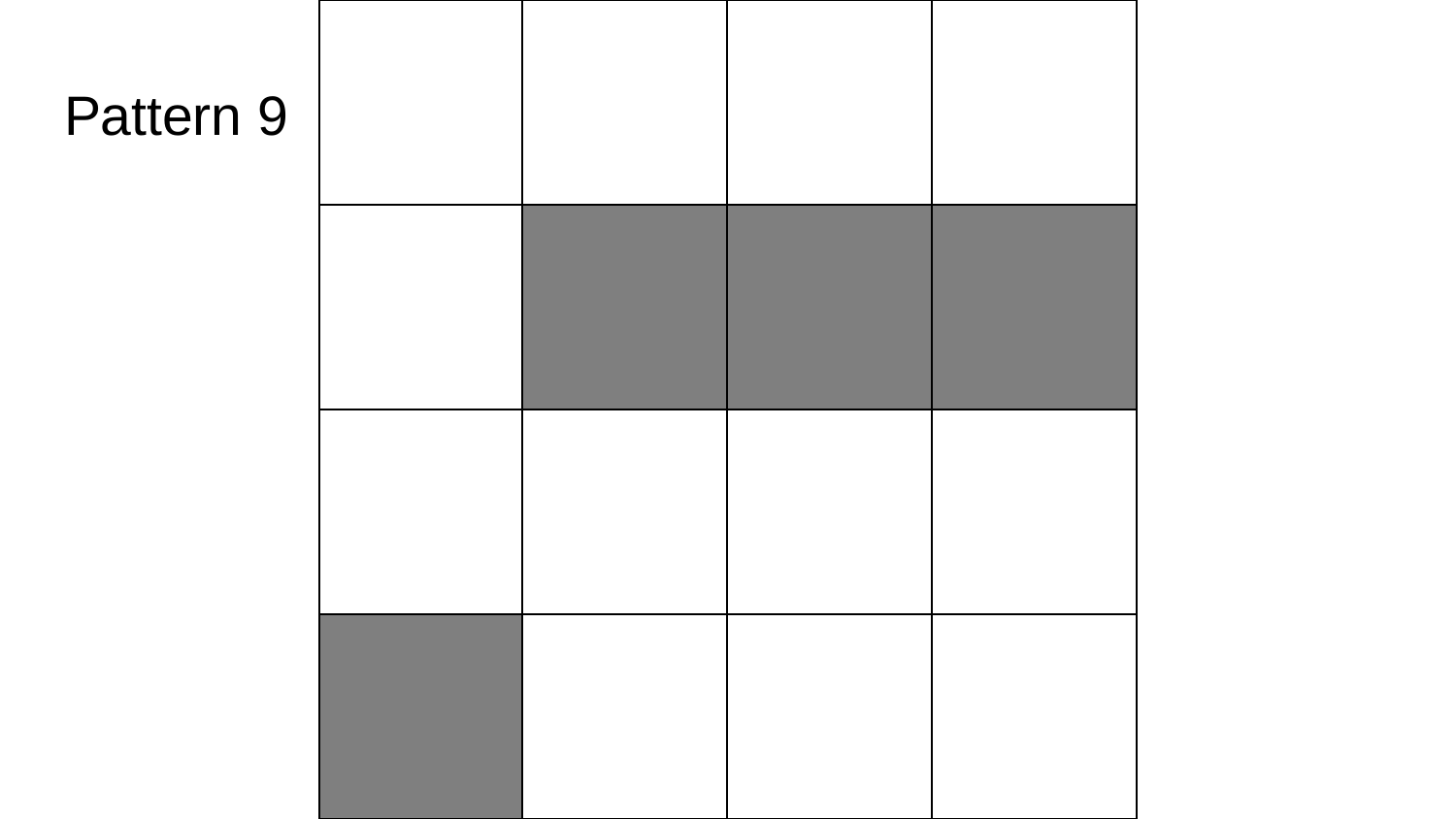

| | | | |
| --- | --- | --- | --- |
| | | | |
| | | | |
| | | | |
# Pattern 9

## Slide 34
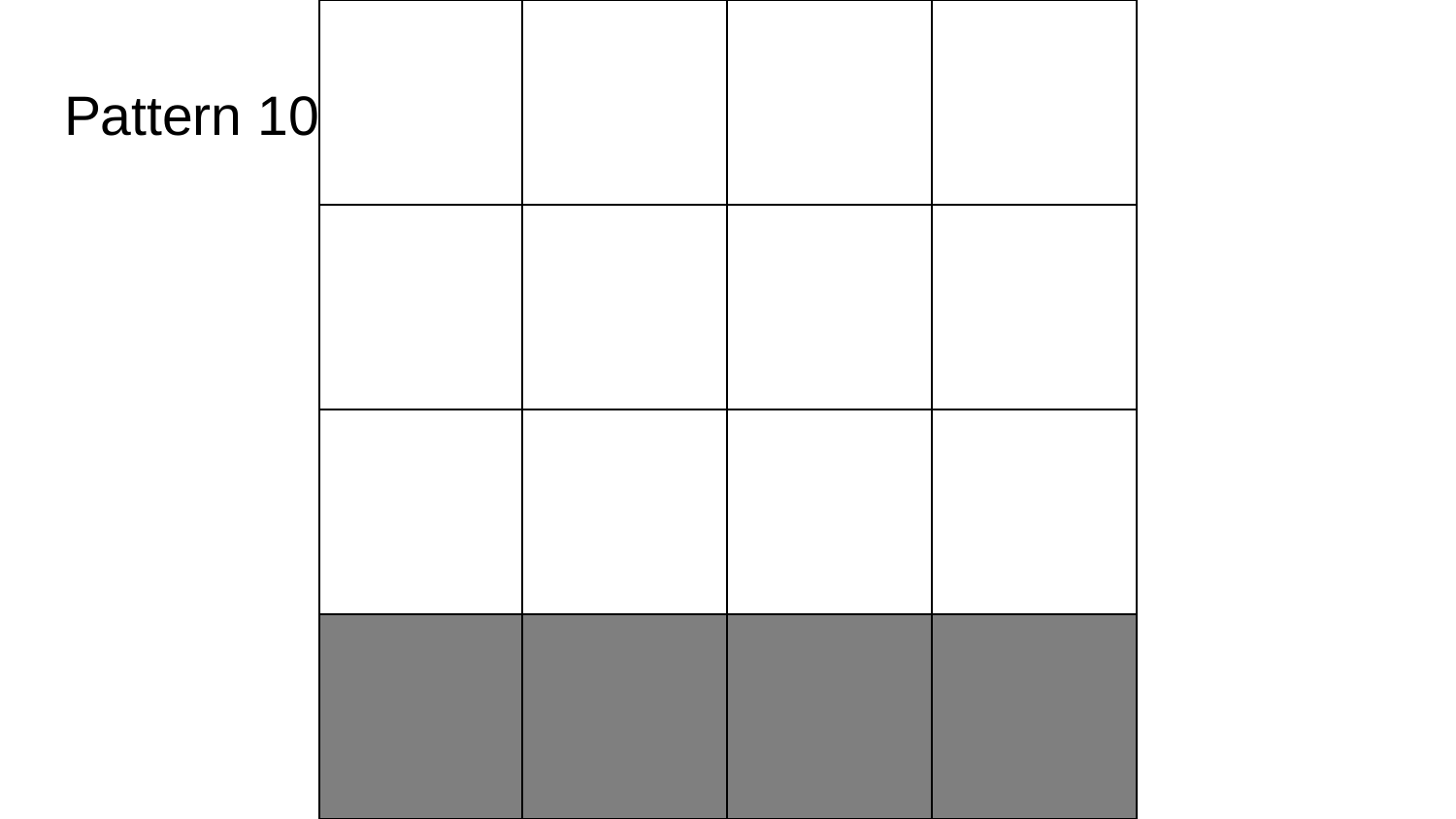

| | | | |
| --- | --- | --- | --- |
| | | | |
| | | | |
| | | | |
# Pattern 10

## Slide 35
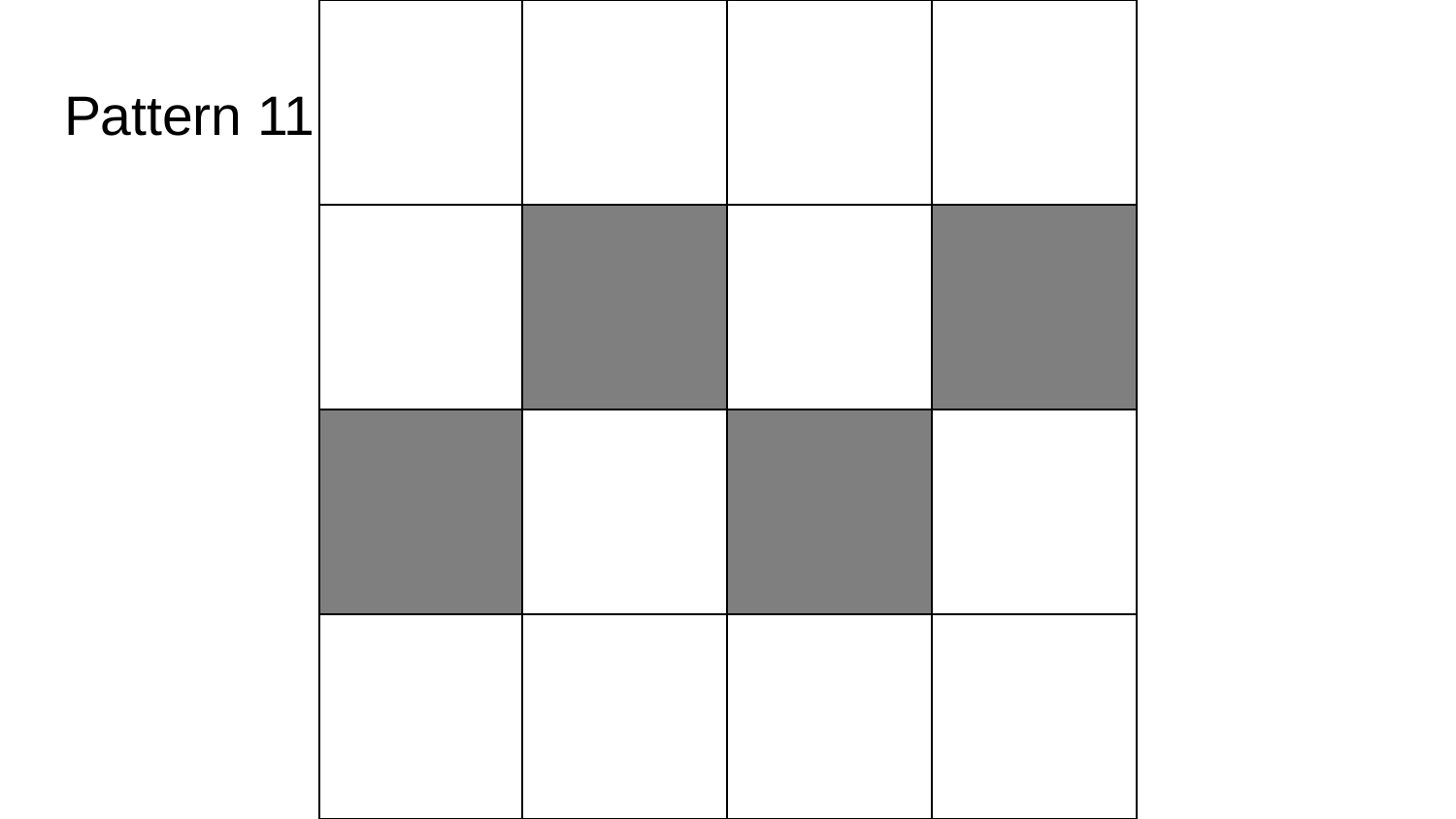

| | | | |
| --- | --- | --- | --- |
| | | | |
| | | | |
| | | | |
# Pattern 11

## Slide 36
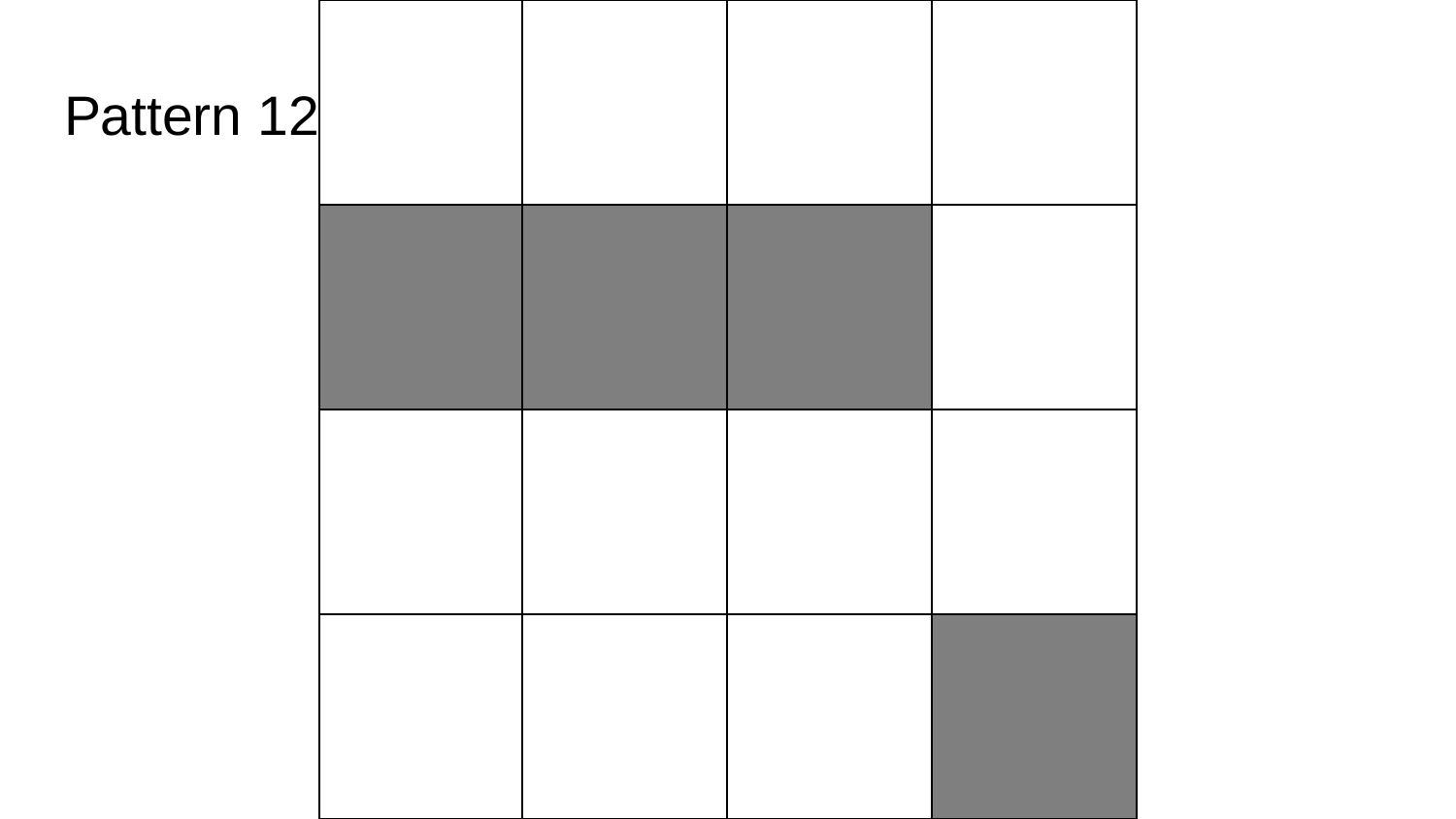

| | | | |
| --- | --- | --- | --- |
| | | | |
| | | | |
| | | | |
# Pattern 12

## Slide 37
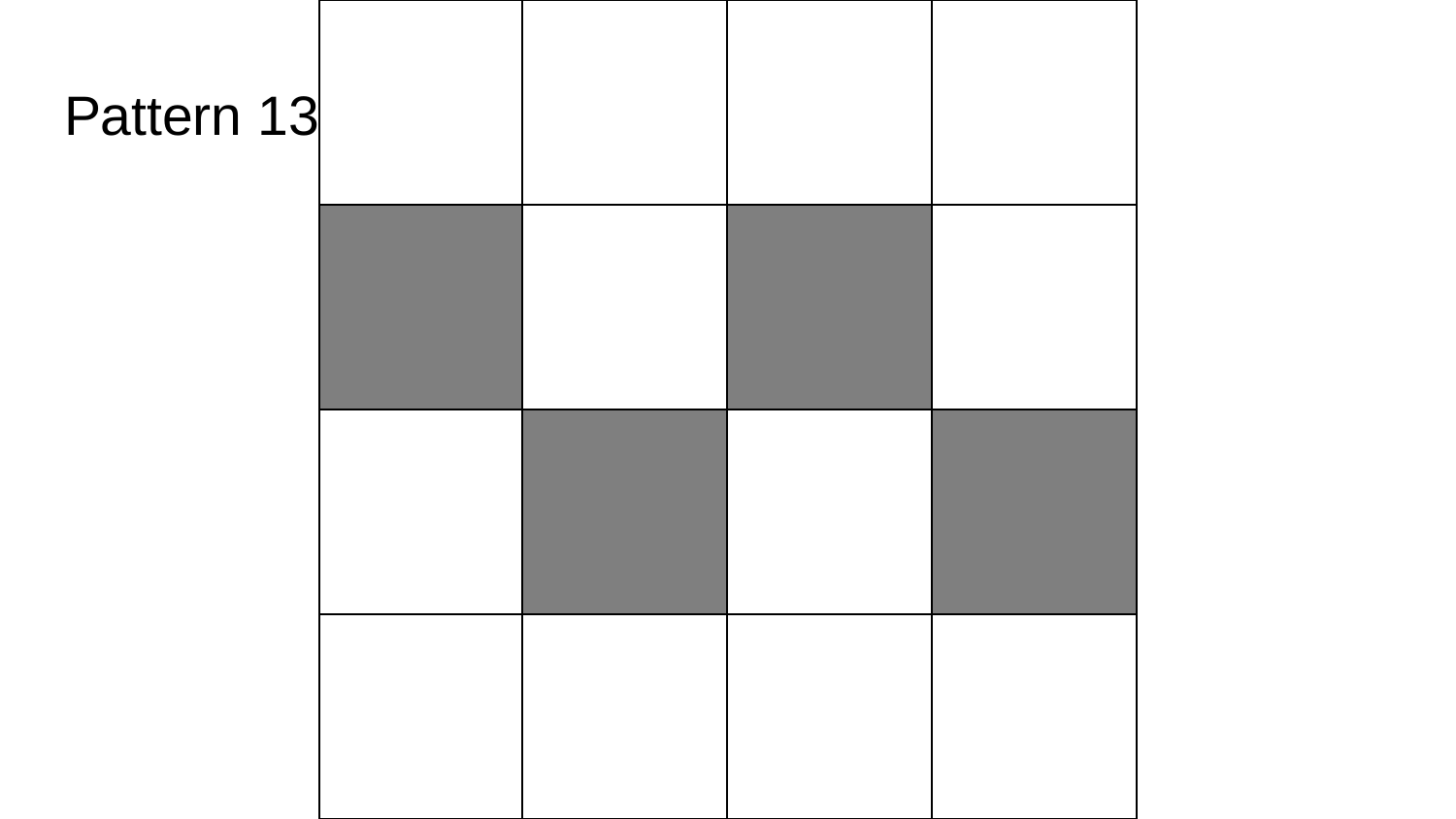

| | | | |
| --- | --- | --- | --- |
| | | | |
| | | | |
| | | | |
# Pattern 13

## Slide 38
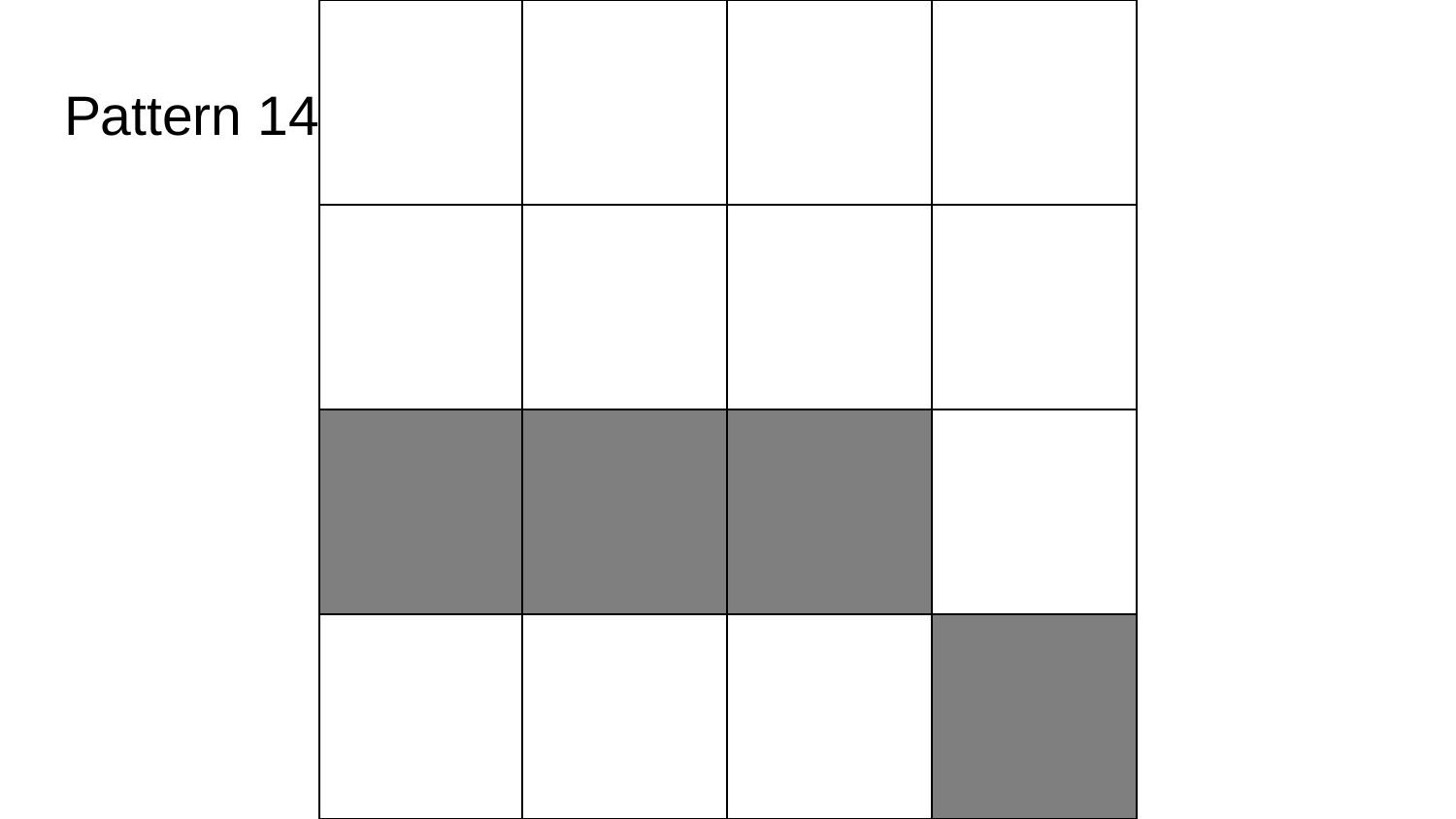

| | | | |
| --- | --- | --- | --- |
| | | | |
| | | | |
| | | | |
# Pattern 14

## Slide 39
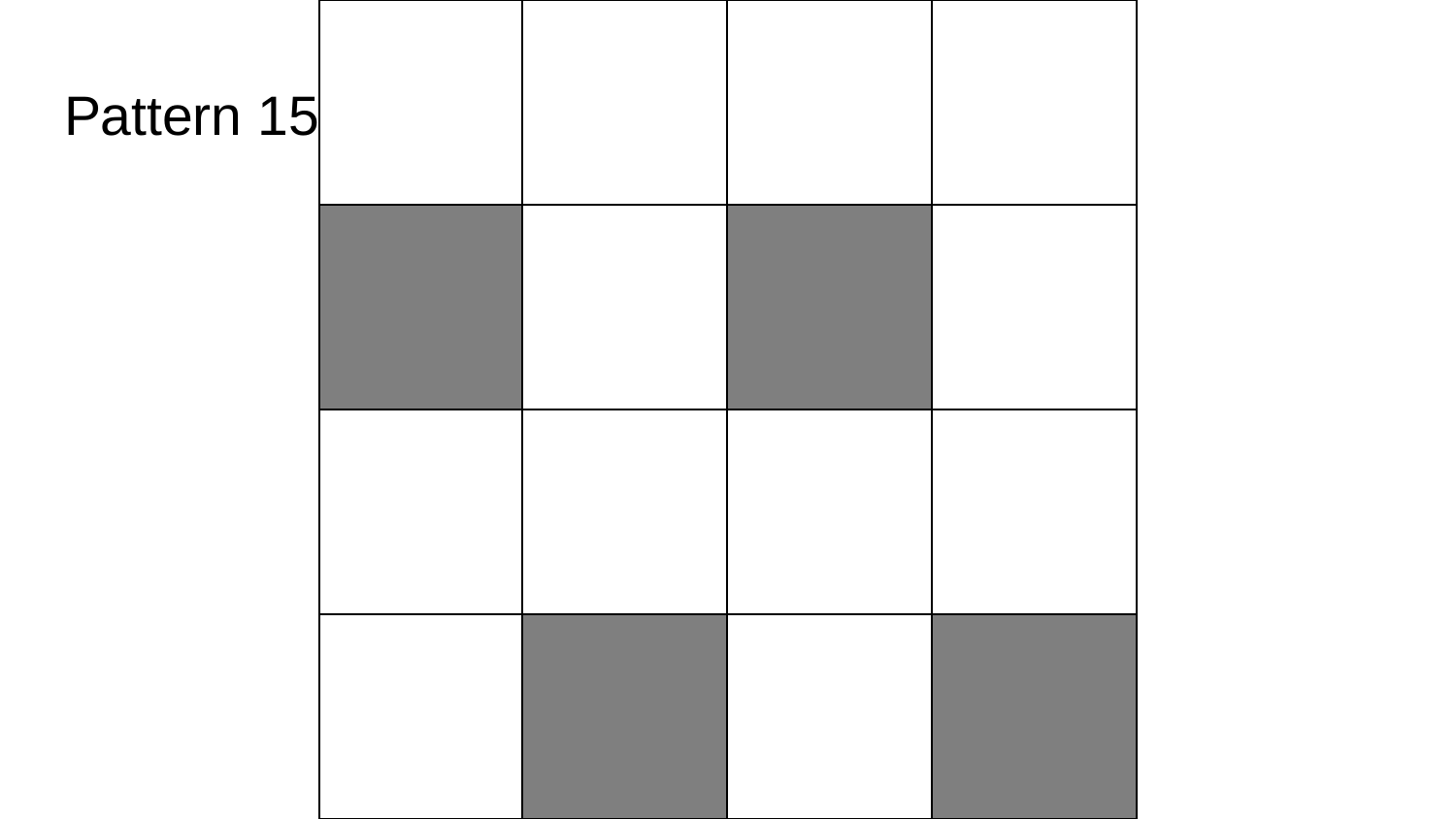

| | | | |
| --- | --- | --- | --- |
| | | | |
| | | | |
| | | | |
# Pattern 15

## Slide 40
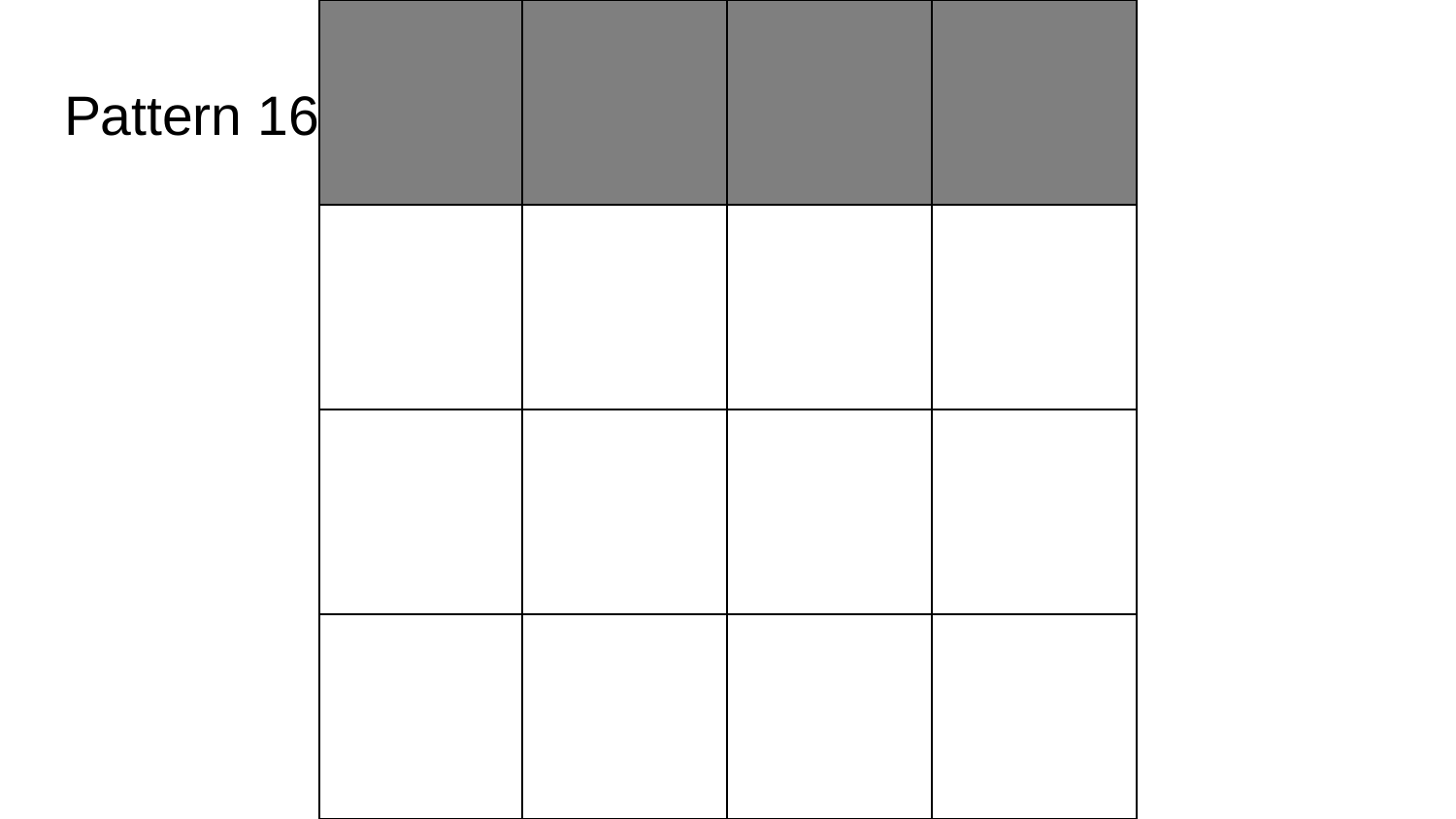

| | | | |
| --- | --- | --- | --- |
| | | | |
| | | | |
| | | | |
# Pattern 16

## Slide 41
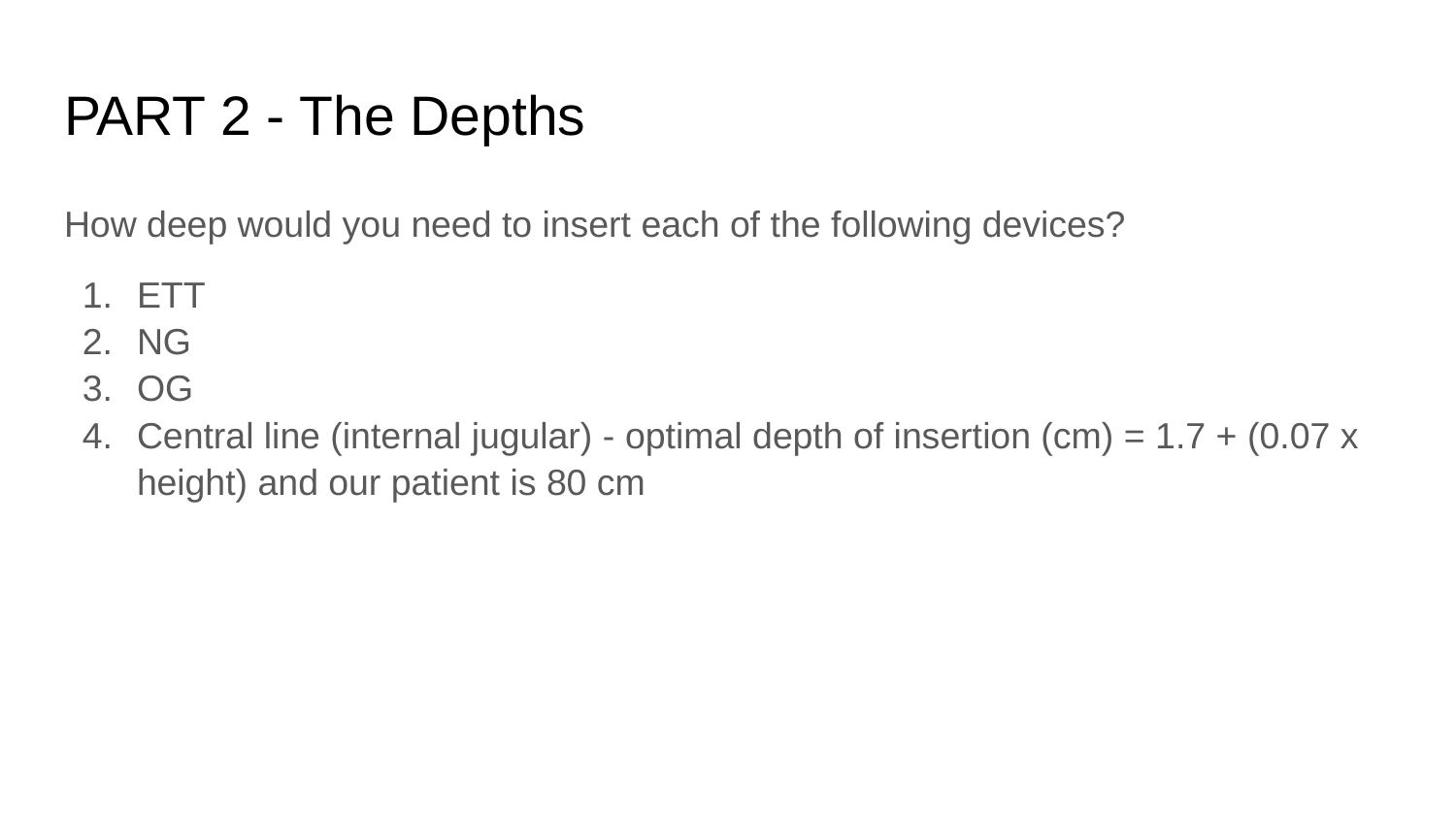

# PART 2 - The Depths
How deep would you need to insert each of the following devices?
ETT
NG
OG
Central line (internal jugular) - optimal depth of insertion (cm) = 1.7 + (0.07 x height) and our patient is 80 cm

## Slide 42
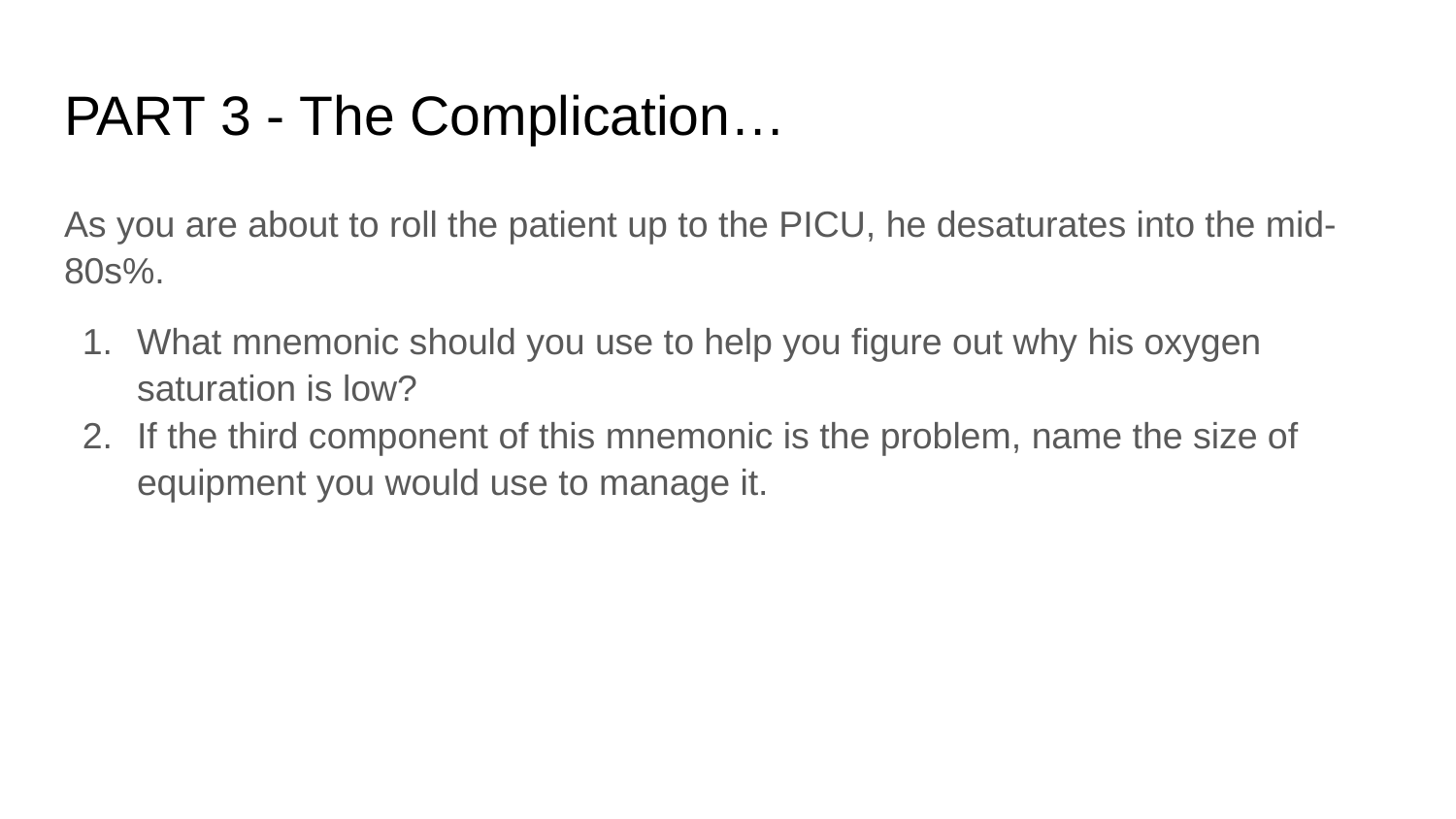

# PART 3 - The Complication…
As you are about to roll the patient up to the PICU, he desaturates into the mid-80s%.
What mnemonic should you use to help you figure out why his oxygen saturation is low?
If the third component of this mnemonic is the problem, name the size of equipment you would use to manage it.

## Slide 43
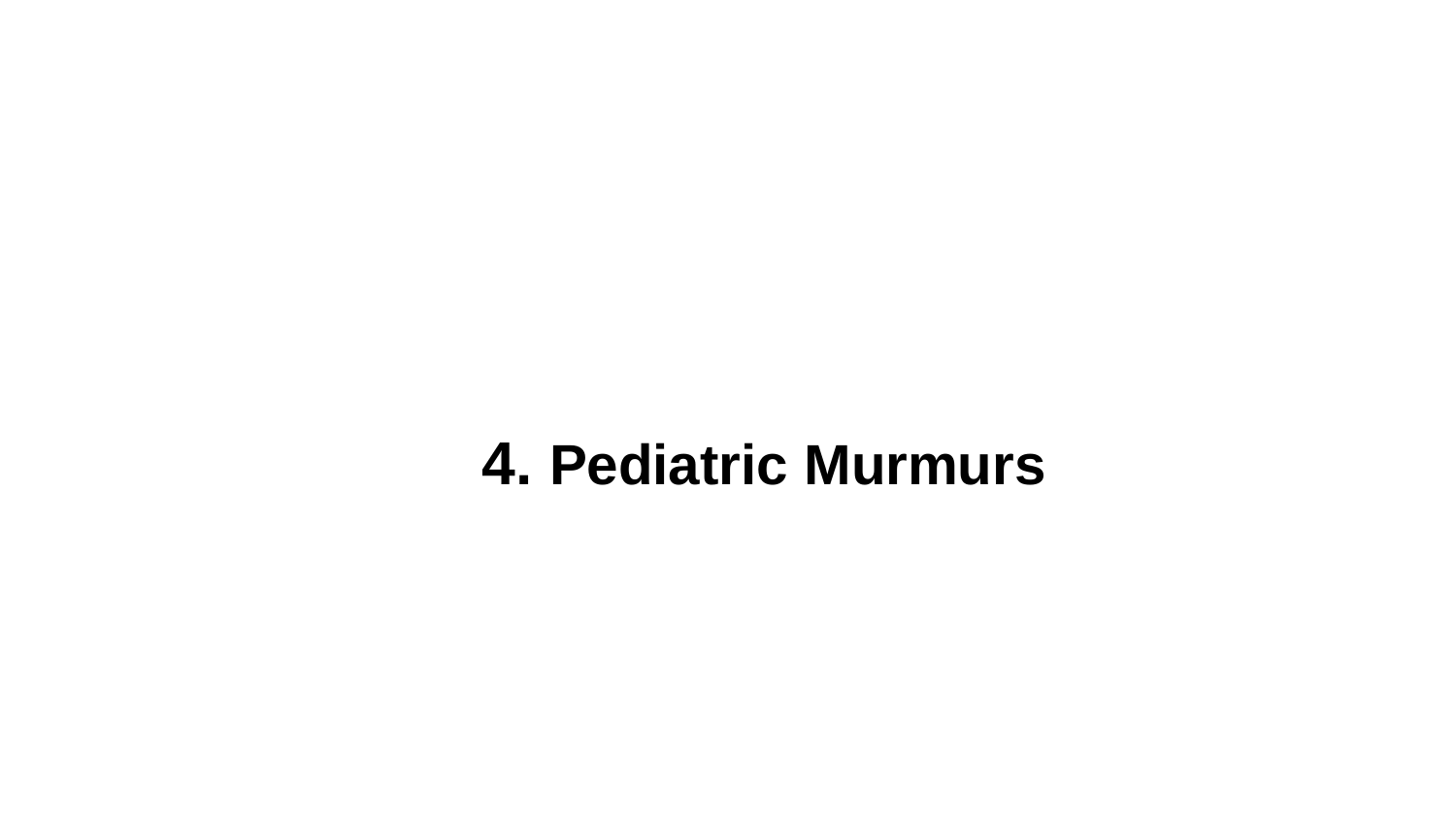

4. Pediatric Murmurs

## Slide 44
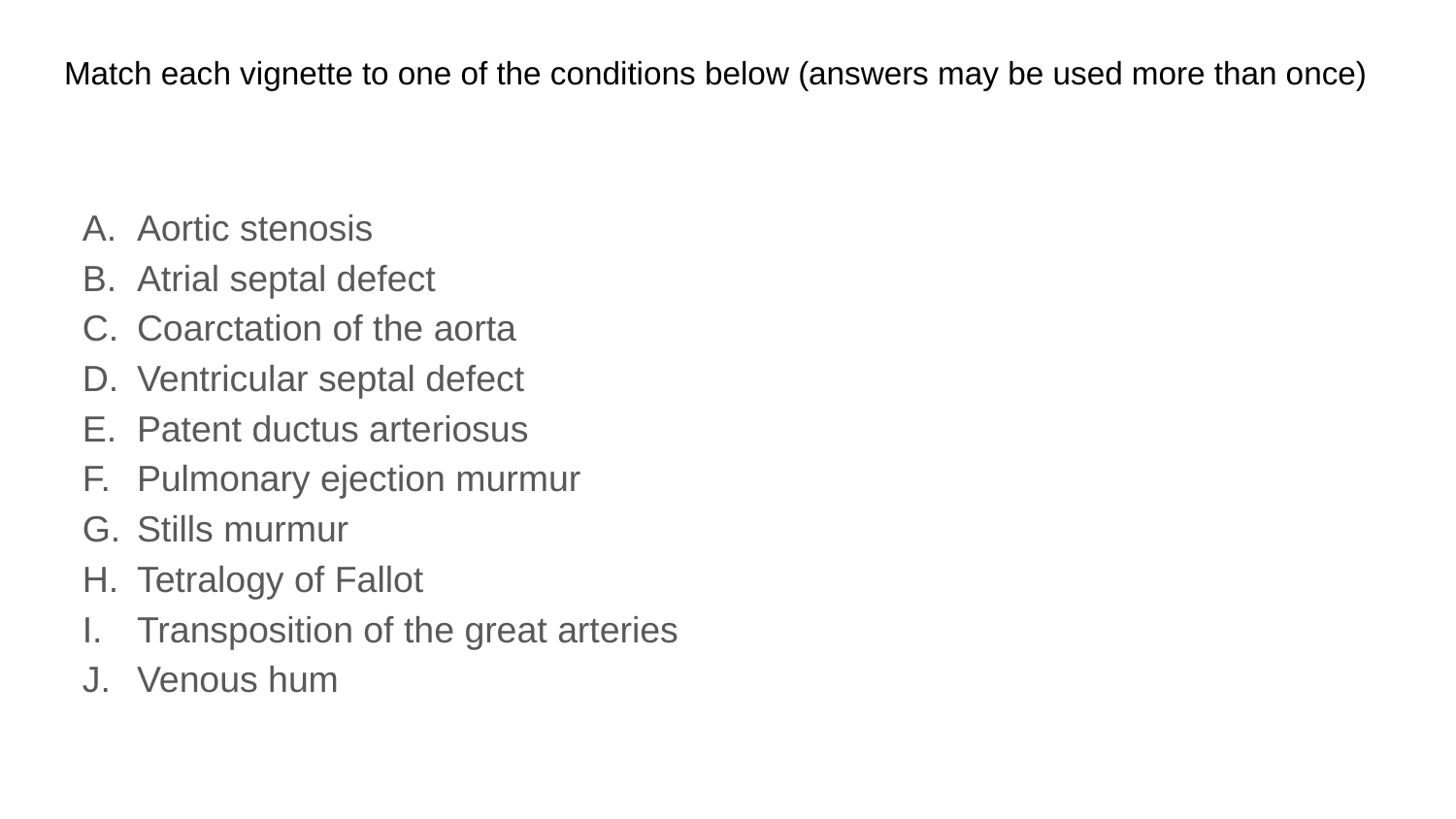

# Match each vignette to one of the conditions below (answers may be used more than once)
Aortic stenosis
Atrial septal defect
Coarctation of the aorta
Ventricular septal defect
Patent ductus arteriosus
Pulmonary ejection murmur
Stills murmur
Tetralogy of Fallot
Transposition of the great arteries
Venous hum

## Slide 45
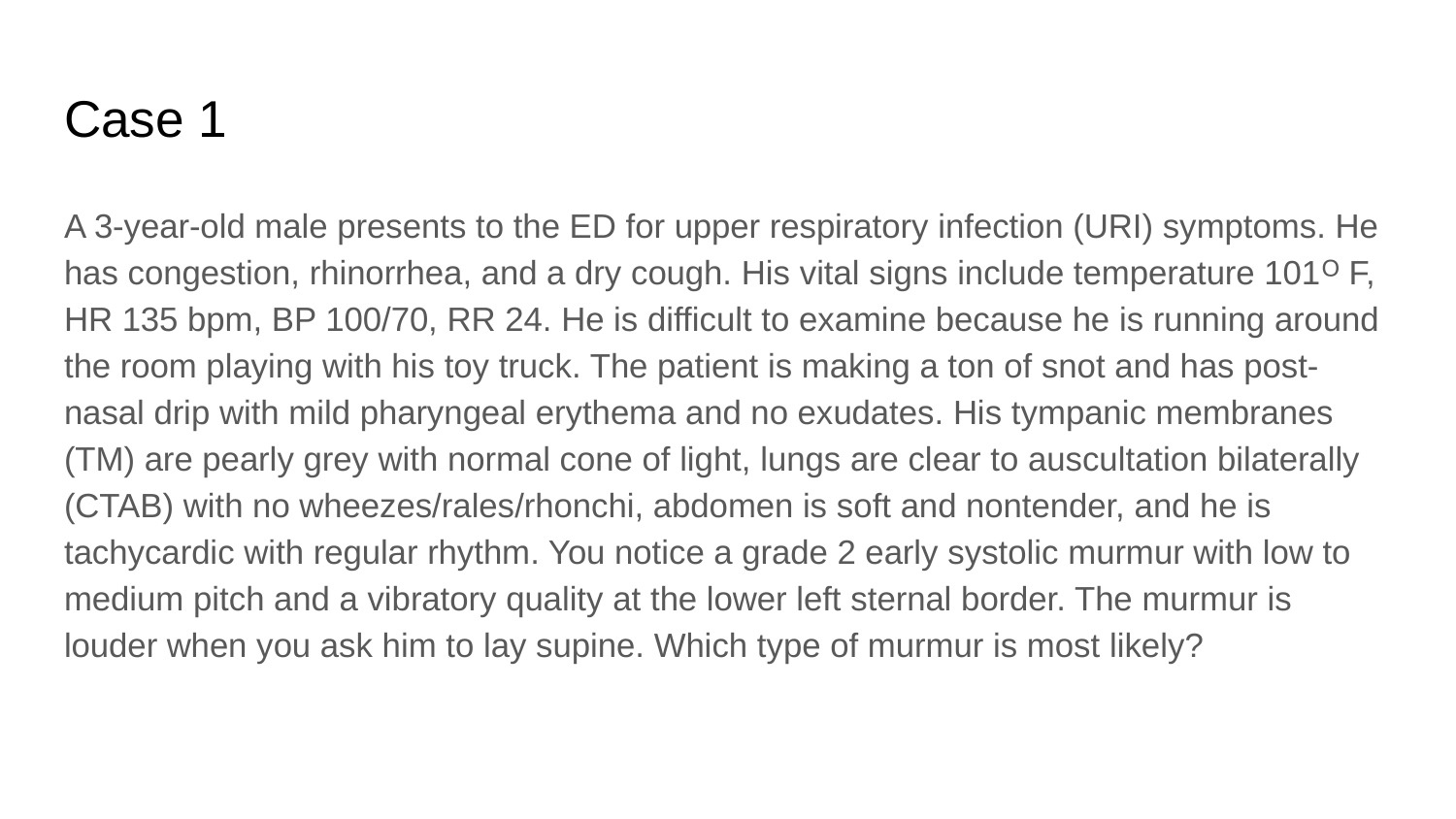

# Case 1
A 3-year-old male presents to the ED for upper respiratory infection (URI) symptoms. He has congestion, rhinorrhea, and a dry cough. His vital signs include temperature 101ᴼ F, HR 135 bpm, BP 100/70, RR 24. He is difficult to examine because he is running around the room playing with his toy truck. The patient is making a ton of snot and has post-nasal drip with mild pharyngeal erythema and no exudates. His tympanic membranes (TM) are pearly grey with normal cone of light, lungs are clear to auscultation bilaterally (CTAB) with no wheezes/rales/rhonchi, abdomen is soft and nontender, and he is tachycardic with regular rhythm. You notice a grade 2 early systolic murmur with low to medium pitch and a vibratory quality at the lower left sternal border. The murmur is louder when you ask him to lay supine. Which type of murmur is most likely?

## Slide 46
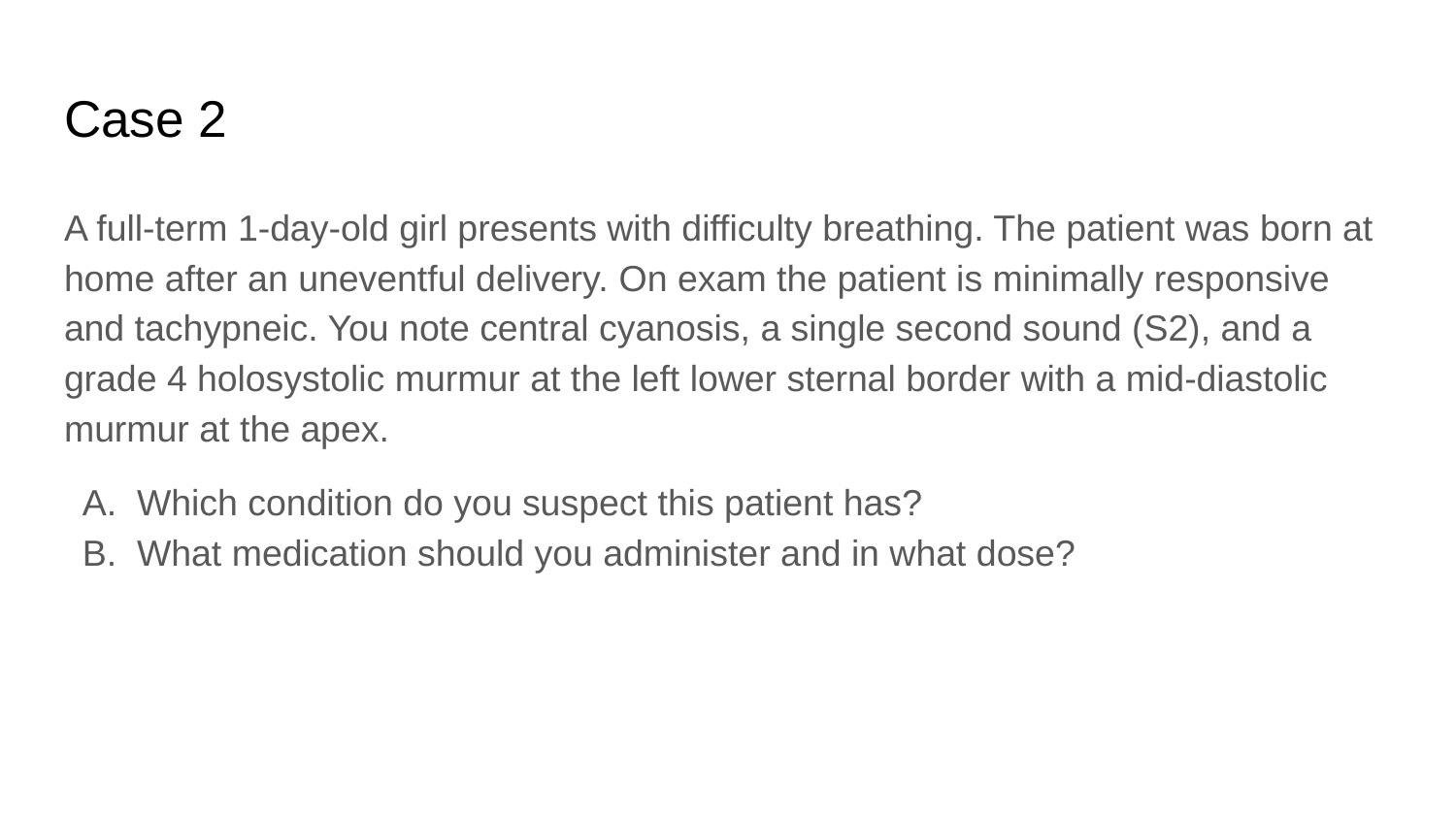

# Case 2
A full-term 1-day-old girl presents with difficulty breathing. The patient was born at home after an uneventful delivery. On exam the patient is minimally responsive and tachypneic. You note central cyanosis, a single second sound (S2), and a grade 4 holosystolic murmur at the left lower sternal border with a mid-diastolic murmur at the apex.
Which condition do you suspect this patient has?
What medication should you administer and in what dose?

## Slide 47
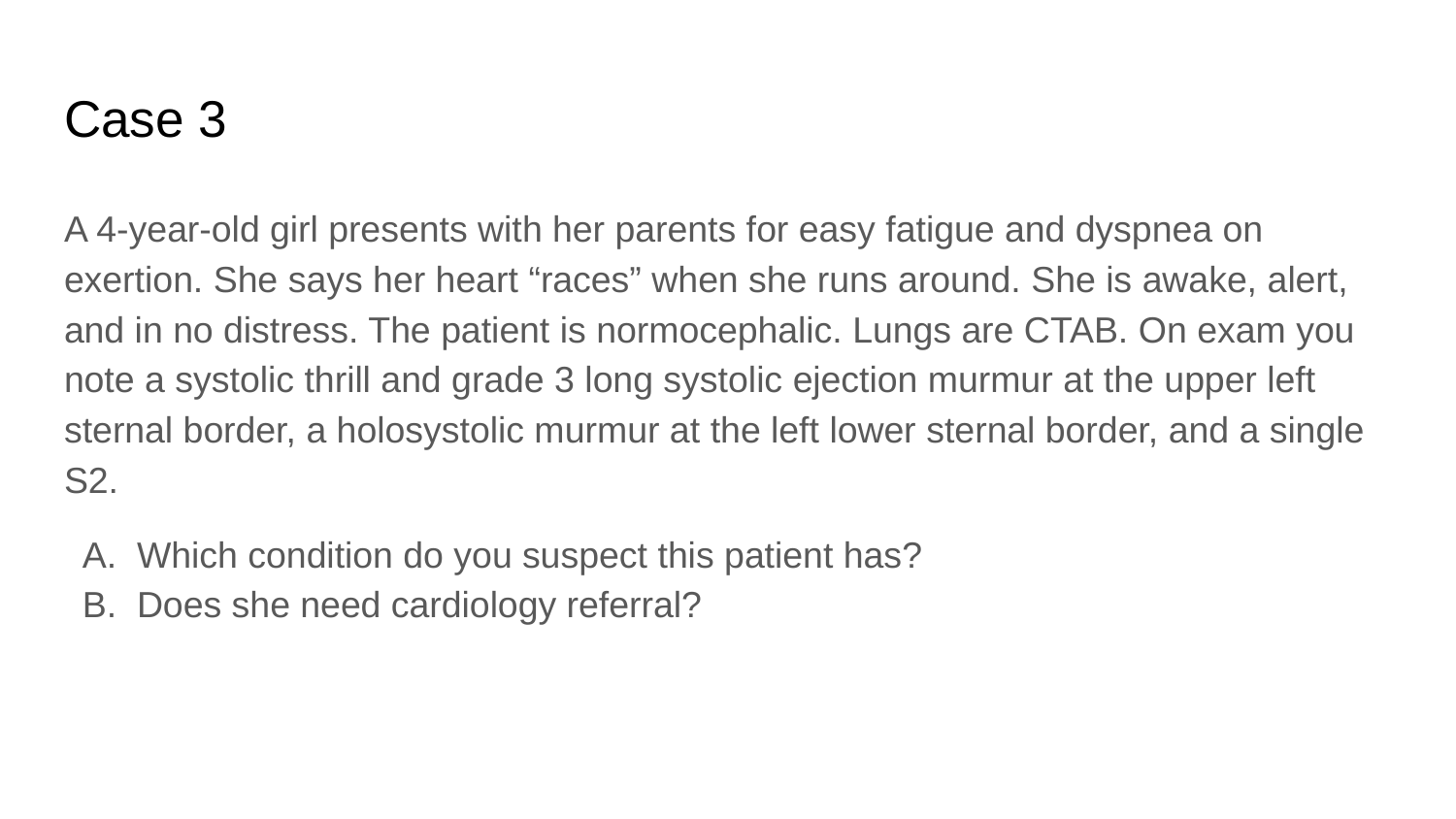

# Case 3
A 4-year-old girl presents with her parents for easy fatigue and dyspnea on exertion. She says her heart “races” when she runs around. She is awake, alert, and in no distress. The patient is normocephalic. Lungs are CTAB. On exam you note a systolic thrill and grade 3 long systolic ejection murmur at the upper left sternal border, a holosystolic murmur at the left lower sternal border, and a single S2.
Which condition do you suspect this patient has?
Does she need cardiology referral?

## Slide 48
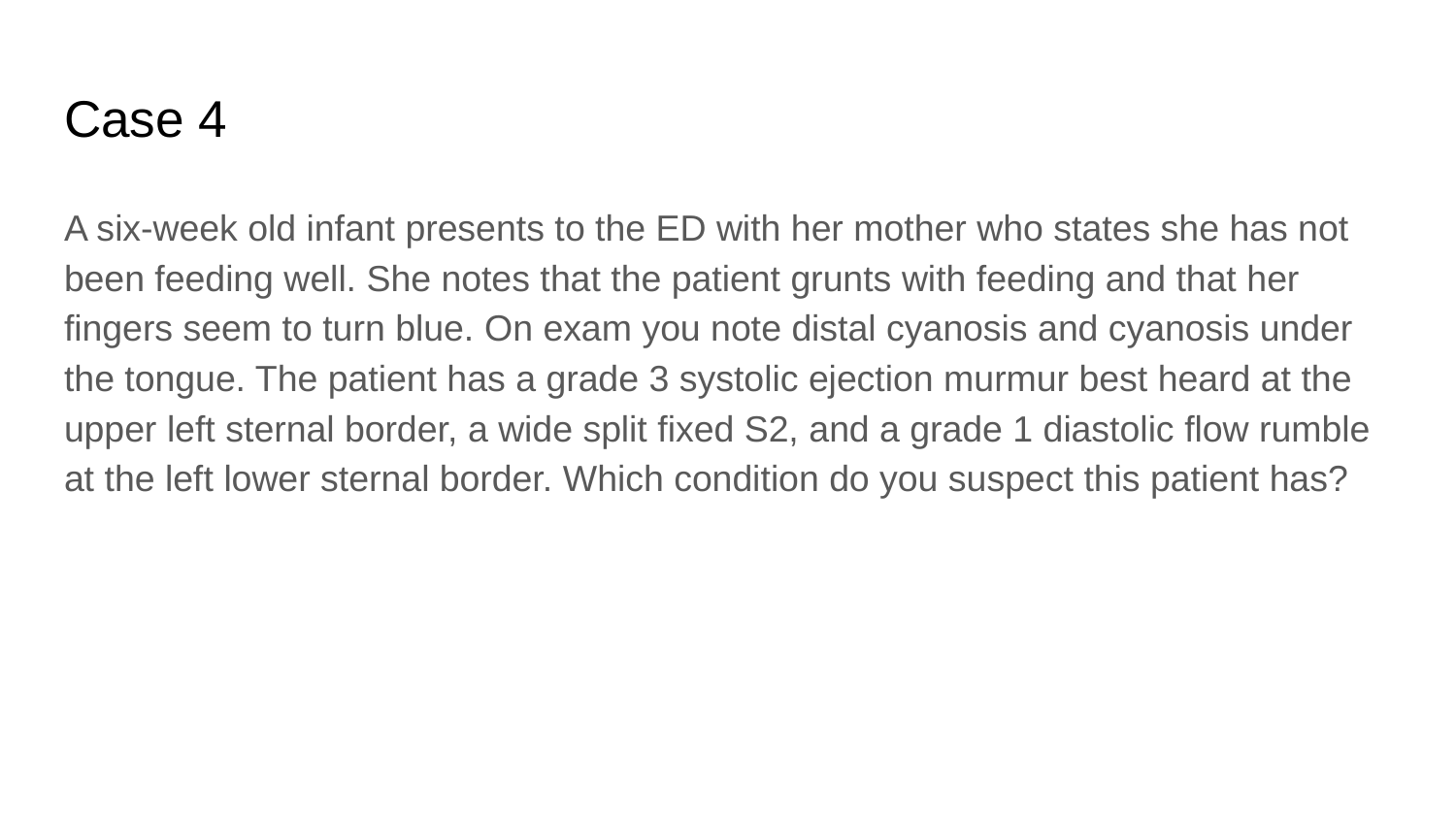

# Case 4
A six-week old infant presents to the ED with her mother who states she has not been feeding well. She notes that the patient grunts with feeding and that her fingers seem to turn blue. On exam you note distal cyanosis and cyanosis under the tongue. The patient has a grade 3 systolic ejection murmur best heard at the upper left sternal border, a wide split fixed S2, and a grade 1 diastolic flow rumble at the left lower sternal border. Which condition do you suspect this patient has?

## Slide 49
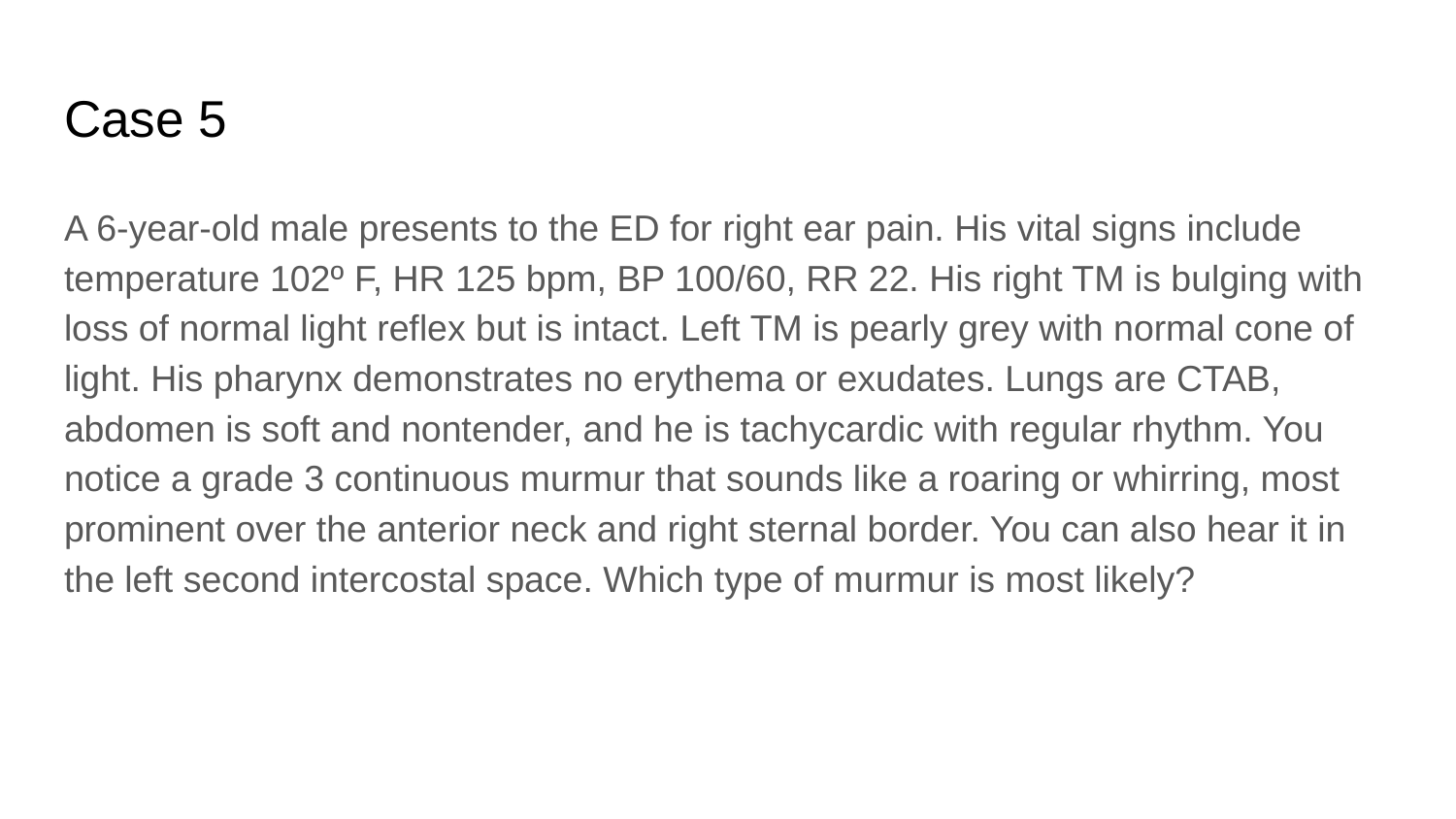

# Case 5
A 6-year-old male presents to the ED for right ear pain. His vital signs include temperature 102º F, HR 125 bpm, BP 100/60, RR 22. His right TM is bulging with loss of normal light reflex but is intact. Left TM is pearly grey with normal cone of light. His pharynx demonstrates no erythema or exudates. Lungs are CTAB, abdomen is soft and nontender, and he is tachycardic with regular rhythm. You notice a grade 3 continuous murmur that sounds like a roaring or whirring, most prominent over the anterior neck and right sternal border. You can also hear it in the left second intercostal space. Which type of murmur is most likely?

## Slide 50
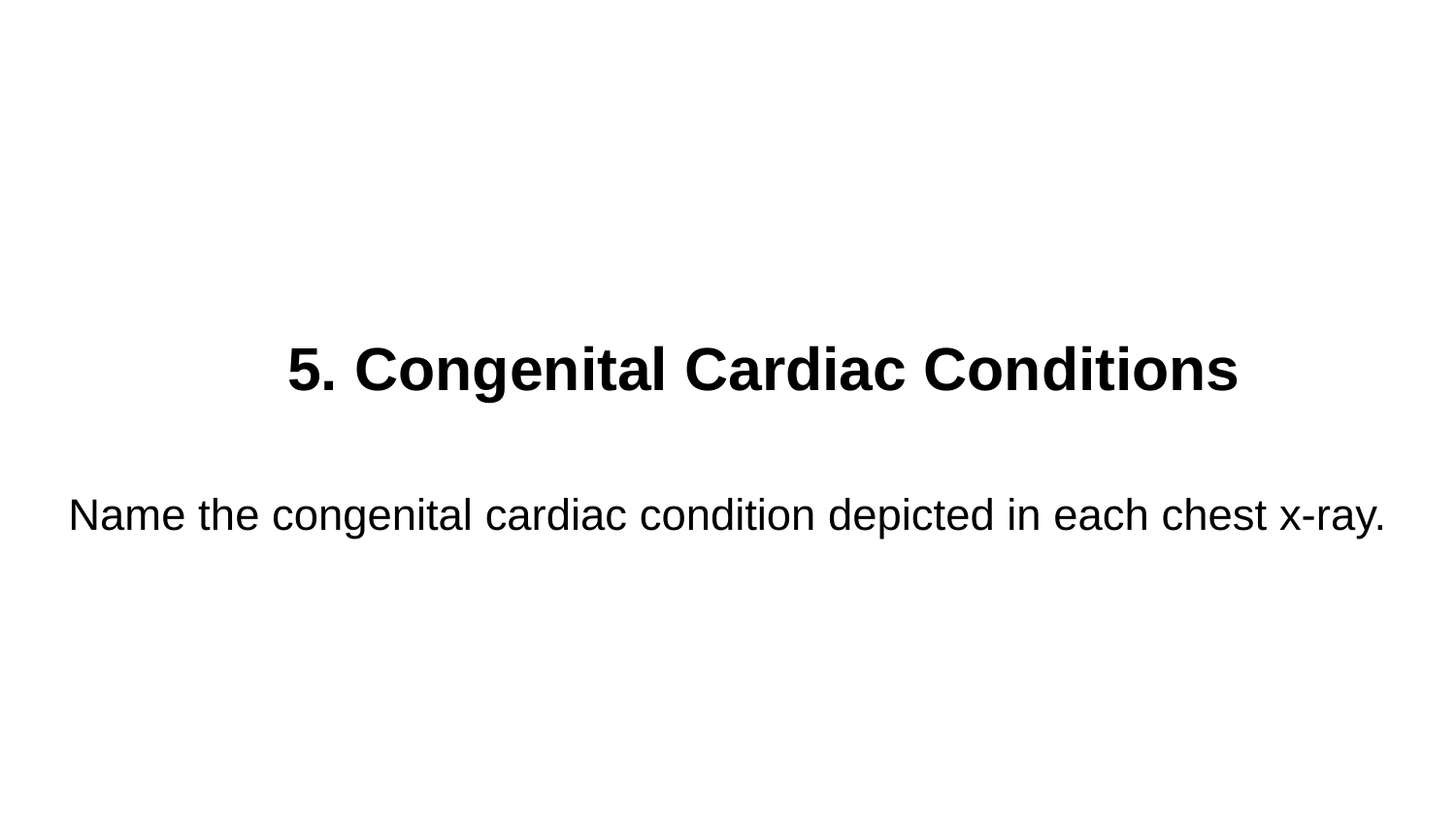

5. Congenital Cardiac Conditions
# Name the congenital cardiac condition depicted in each chest x-ray.

## Slide 51
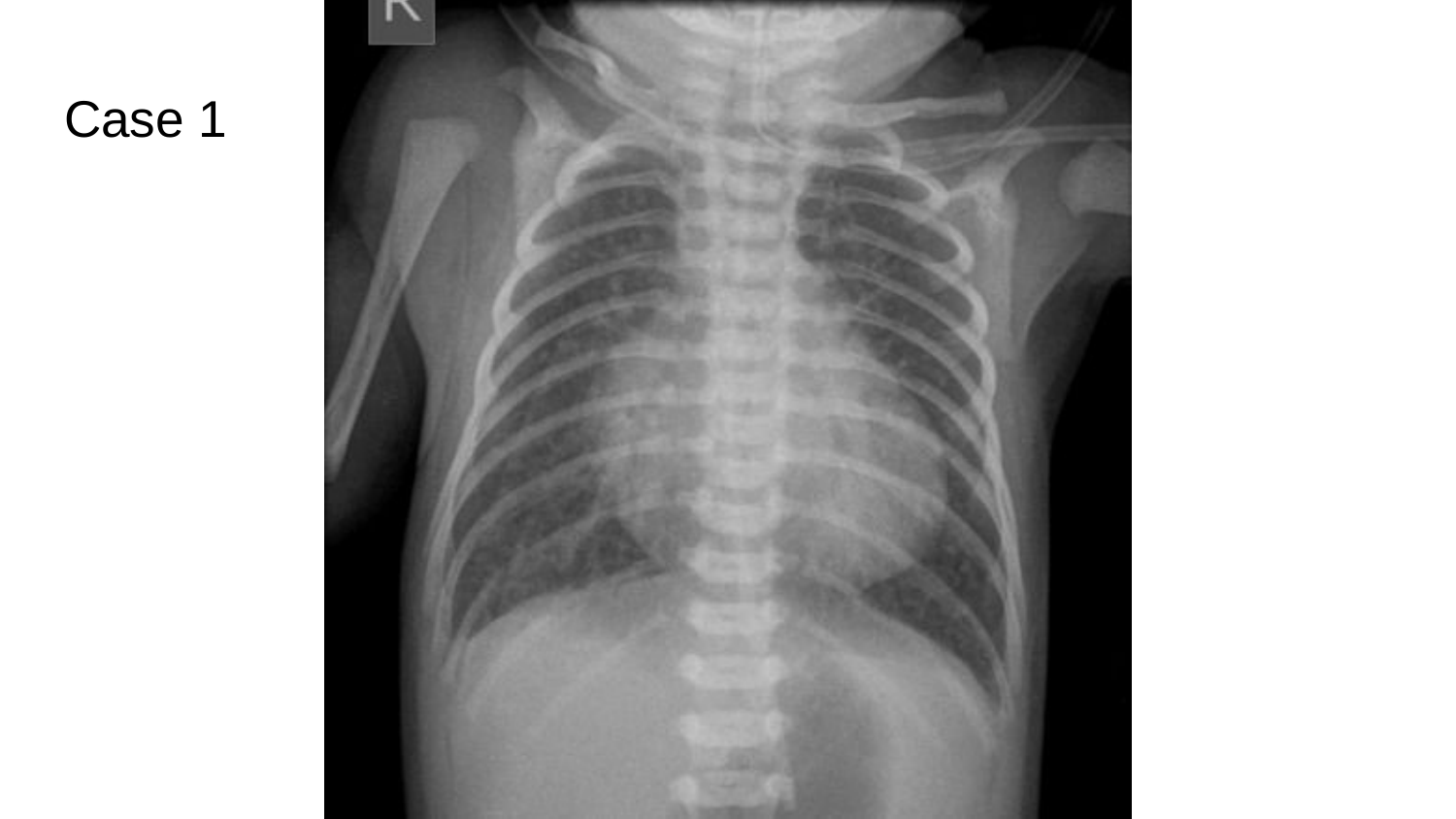

# Case 1

## Slide 52
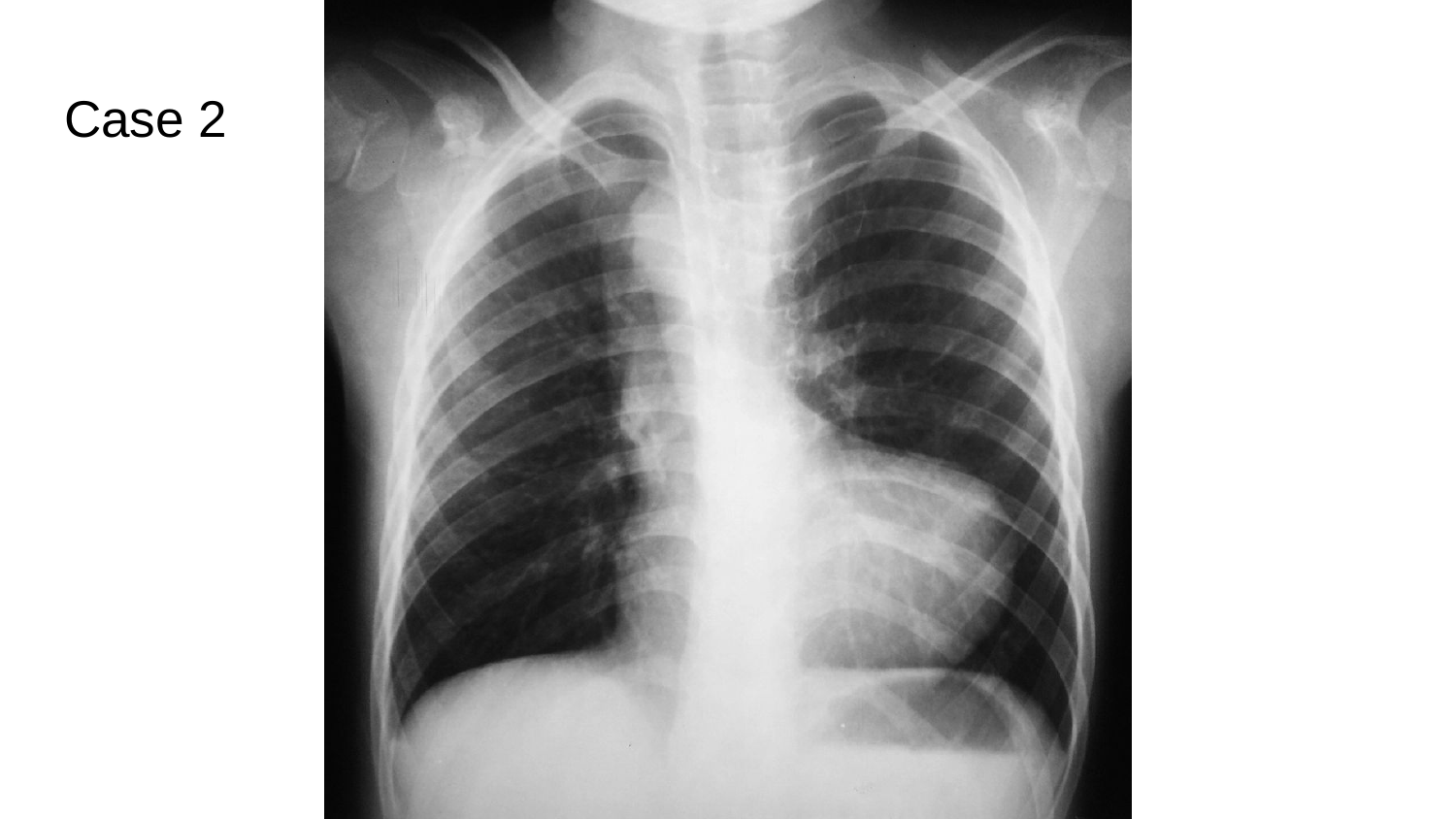

# Case 2

## Slide 53
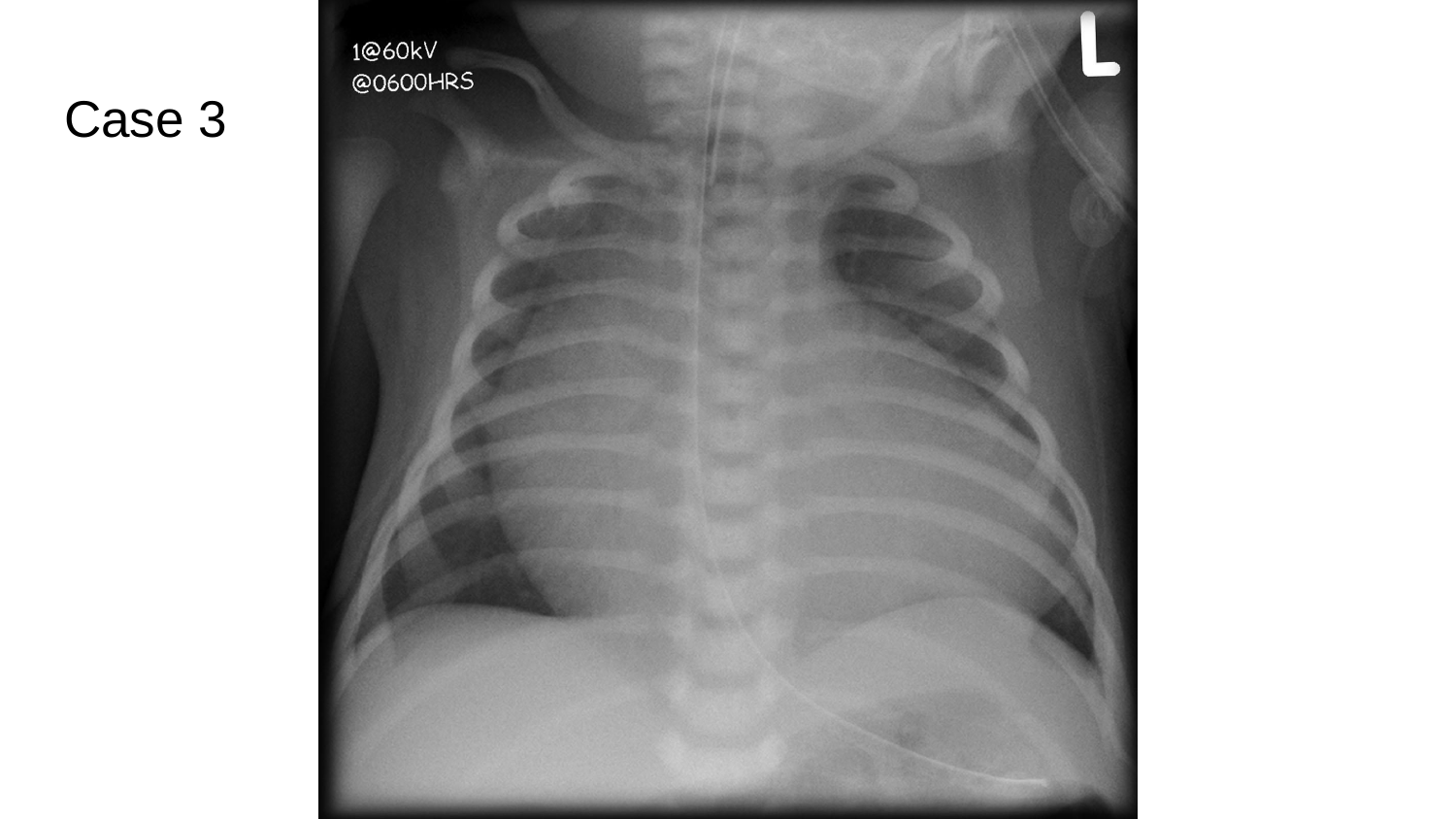

# Case 3

## Slide 54
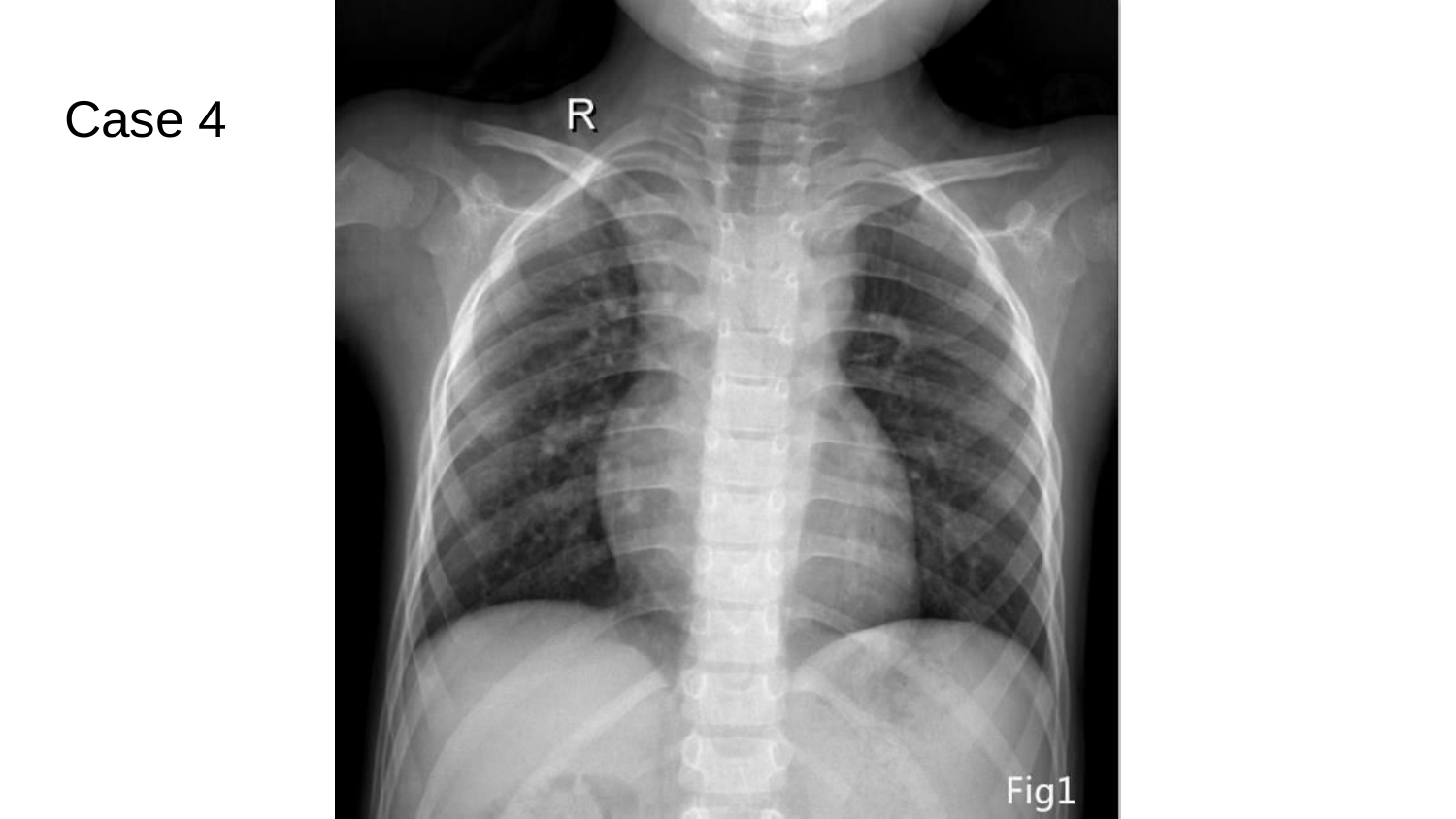

# Case 4

## Slide 55
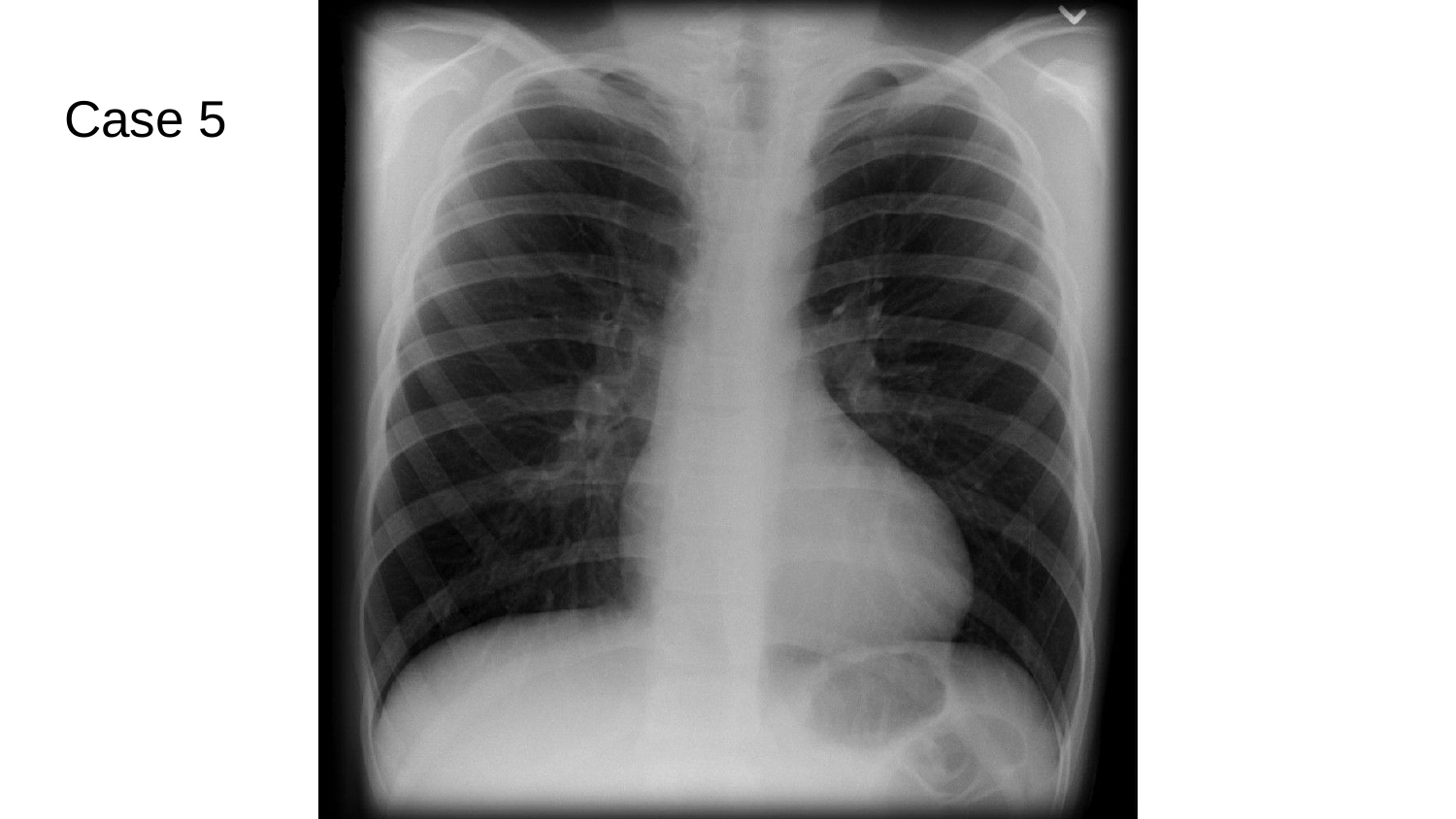

# Case 5

## Slide 56
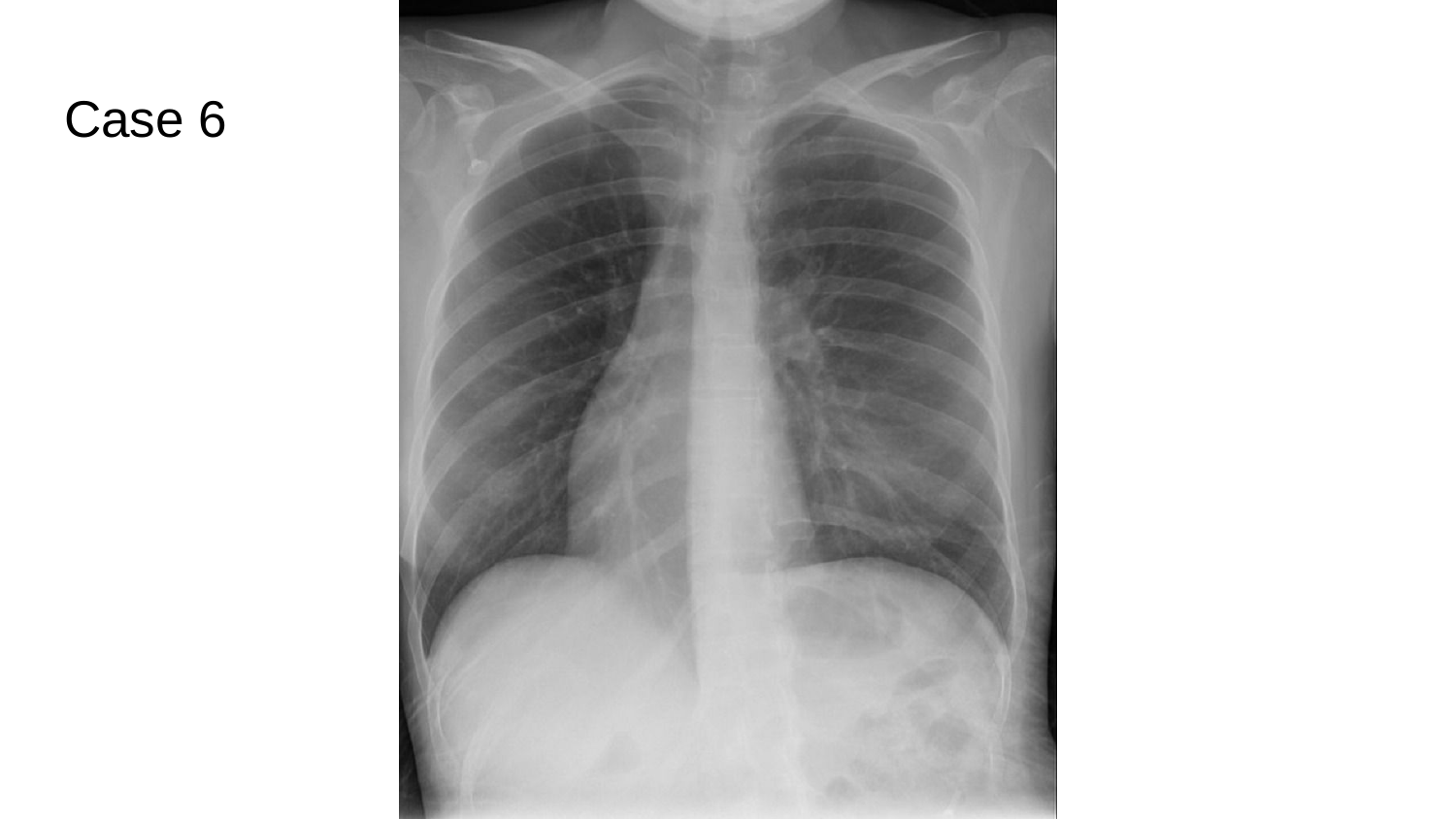

# Case 6

## Slide 57
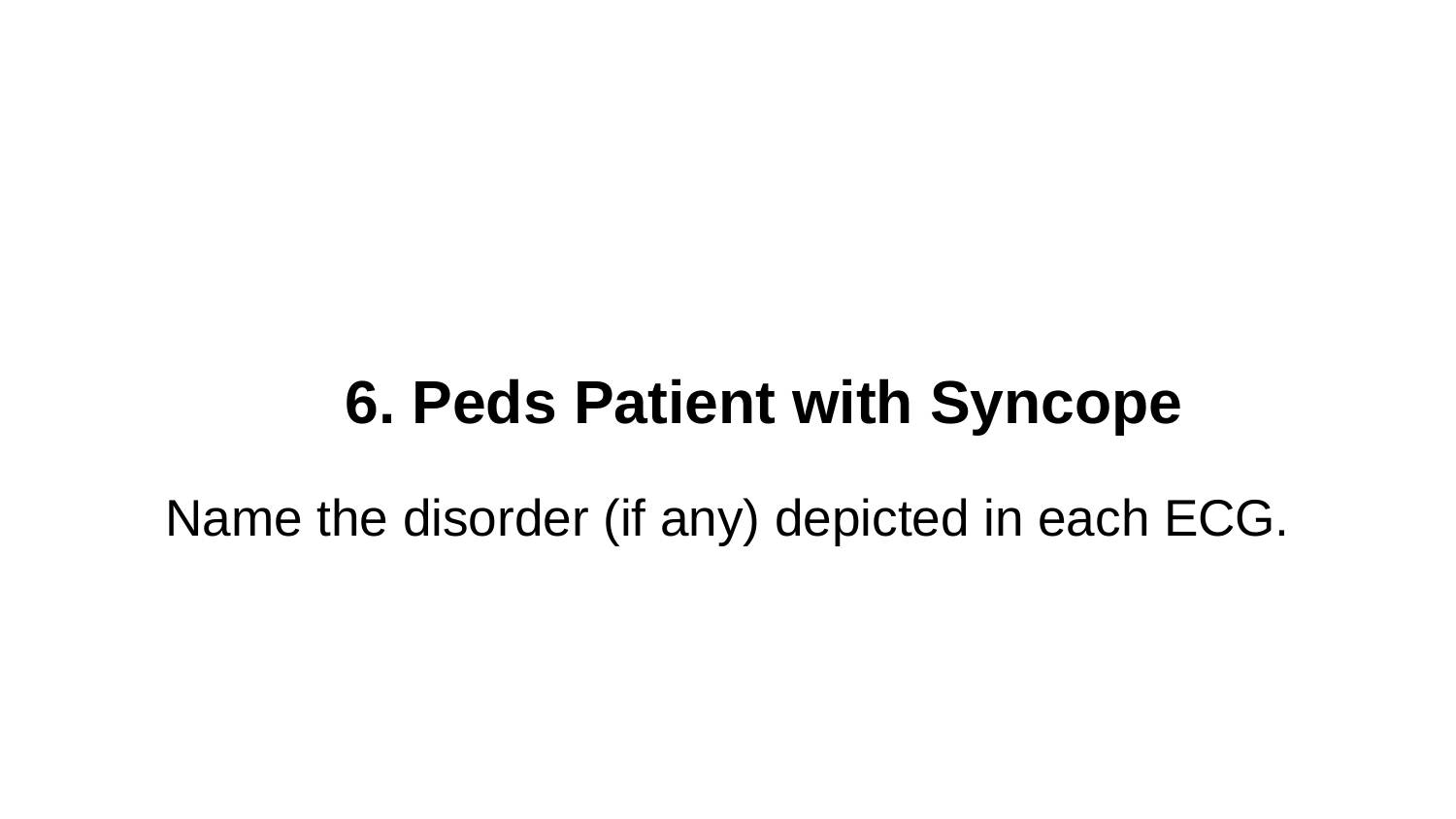

6. Peds Patient with Syncope
# Name the disorder (if any) depicted in each ECG.

## Slide 58
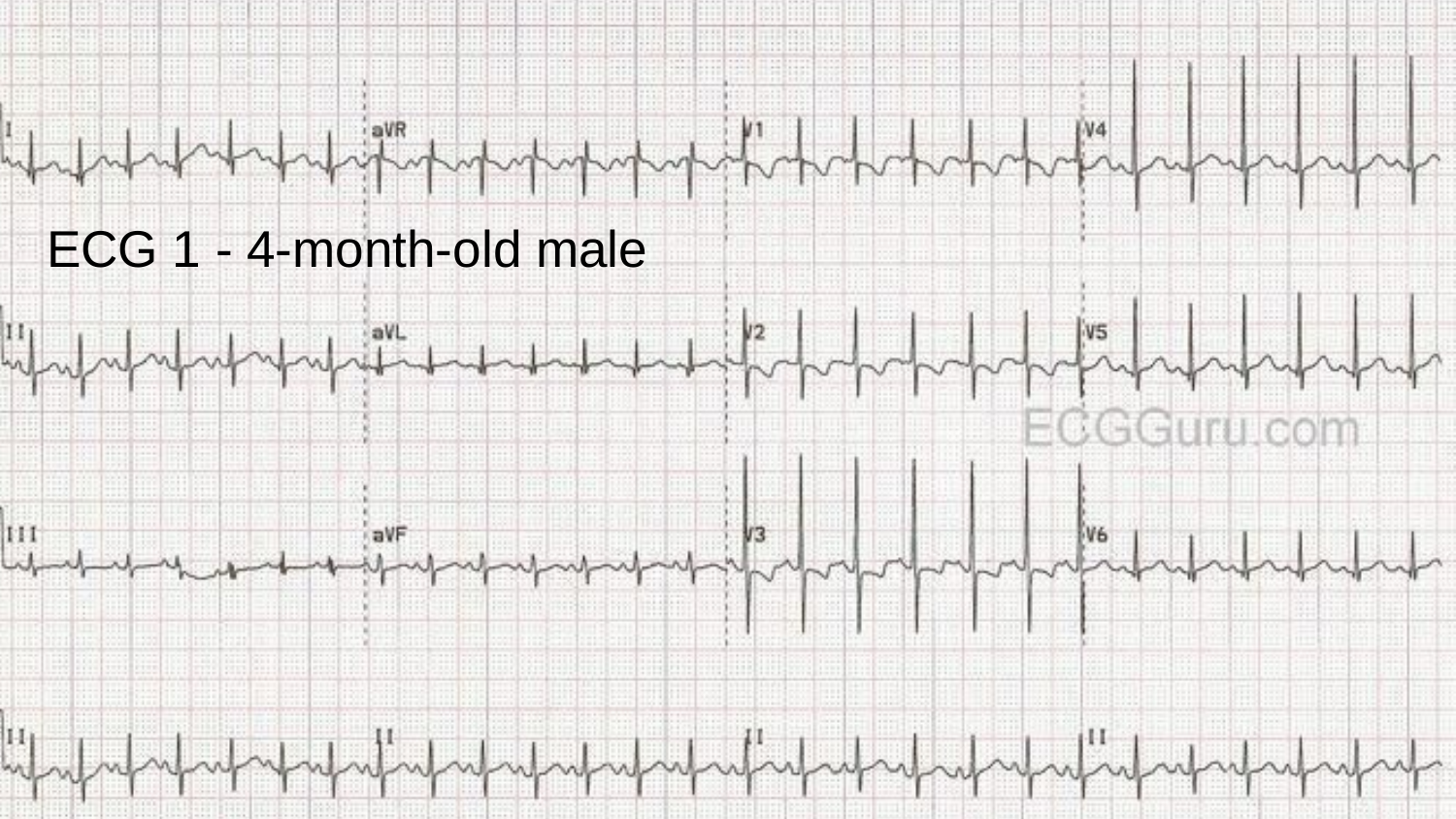

# ECG 1 - 4-month-old male

## Slide 59
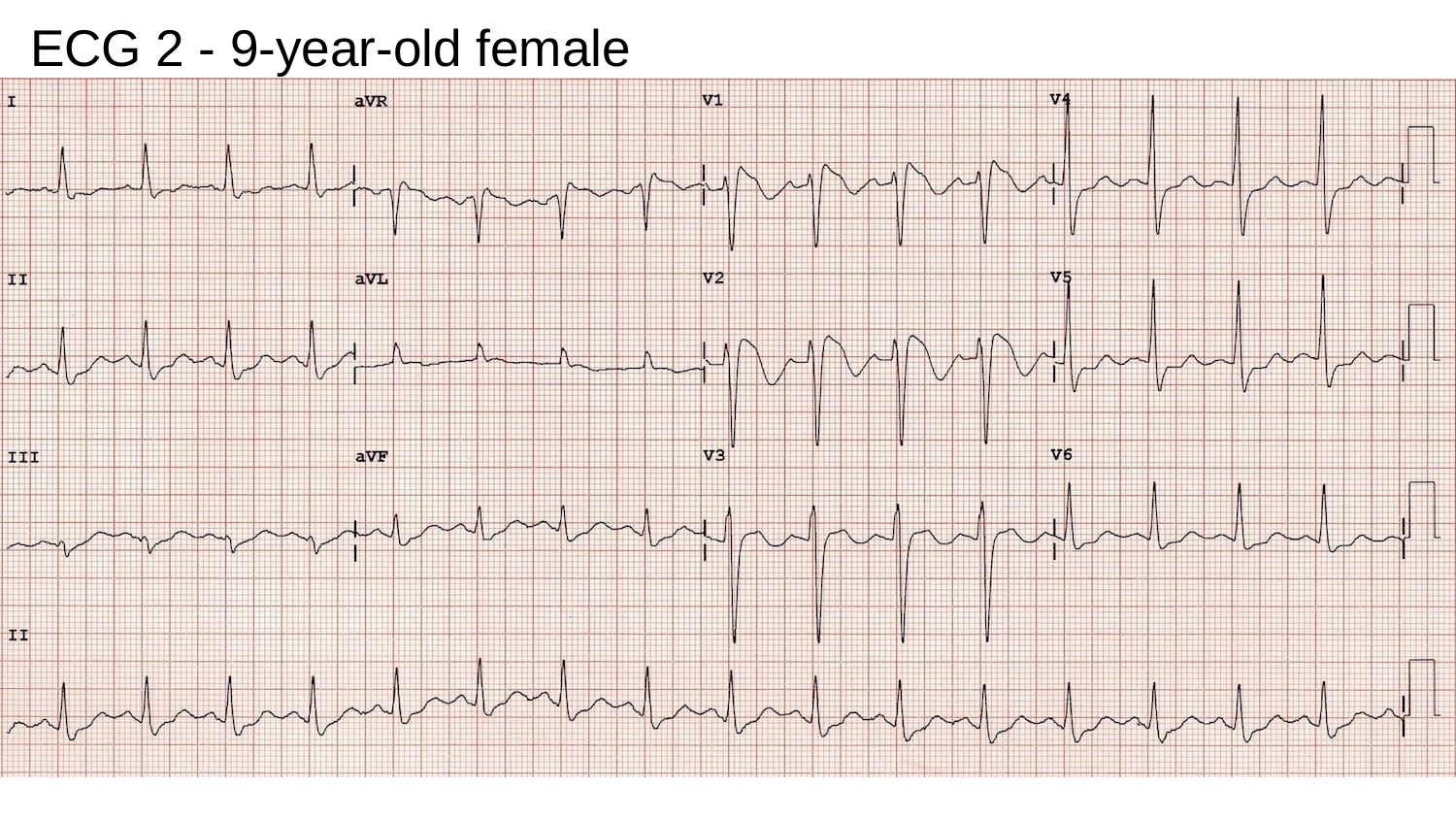

# ECG 2 - 9-year-old female

## Slide 60
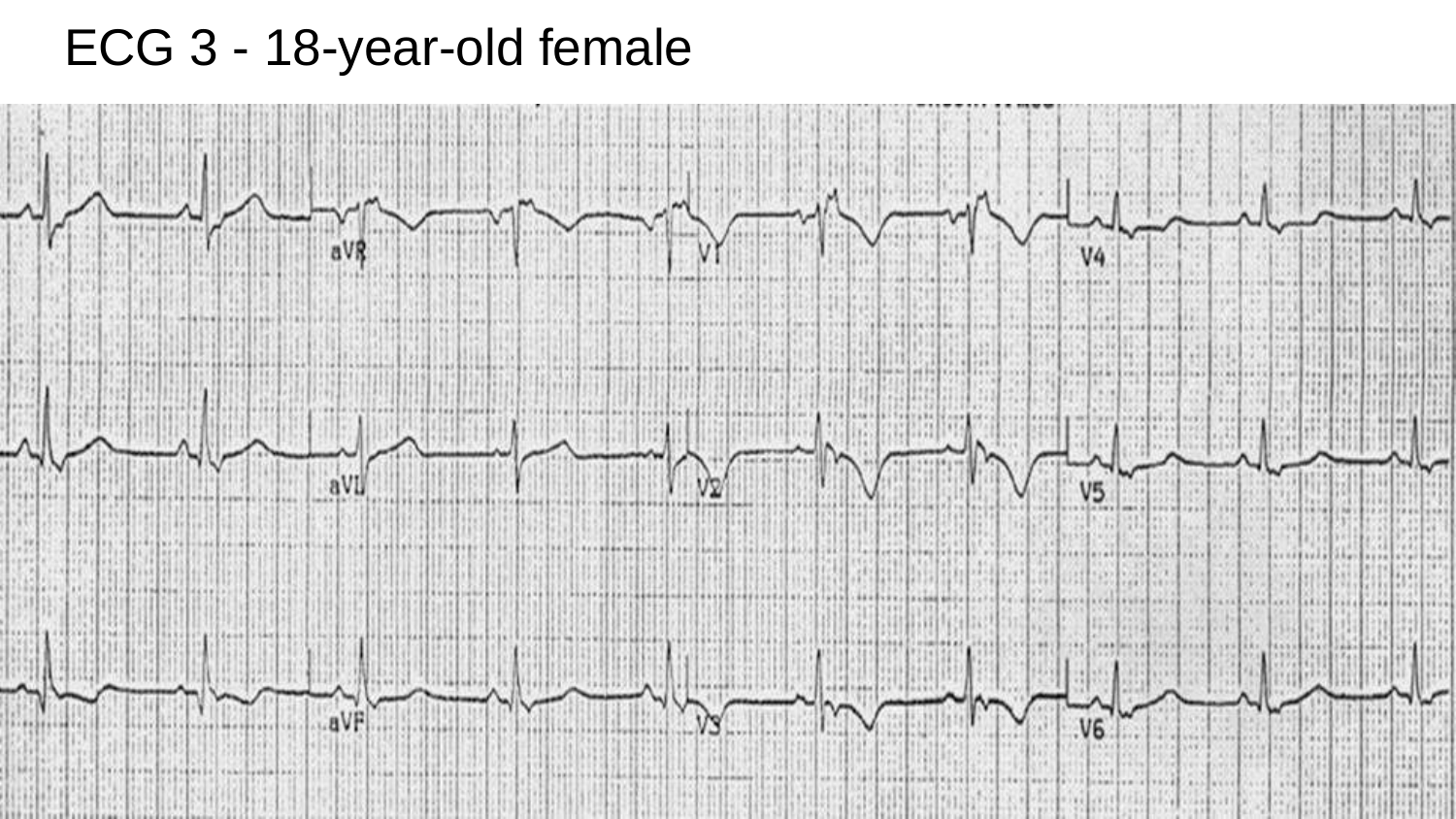

# ECG 3 - 18-year-old female

## Slide 61
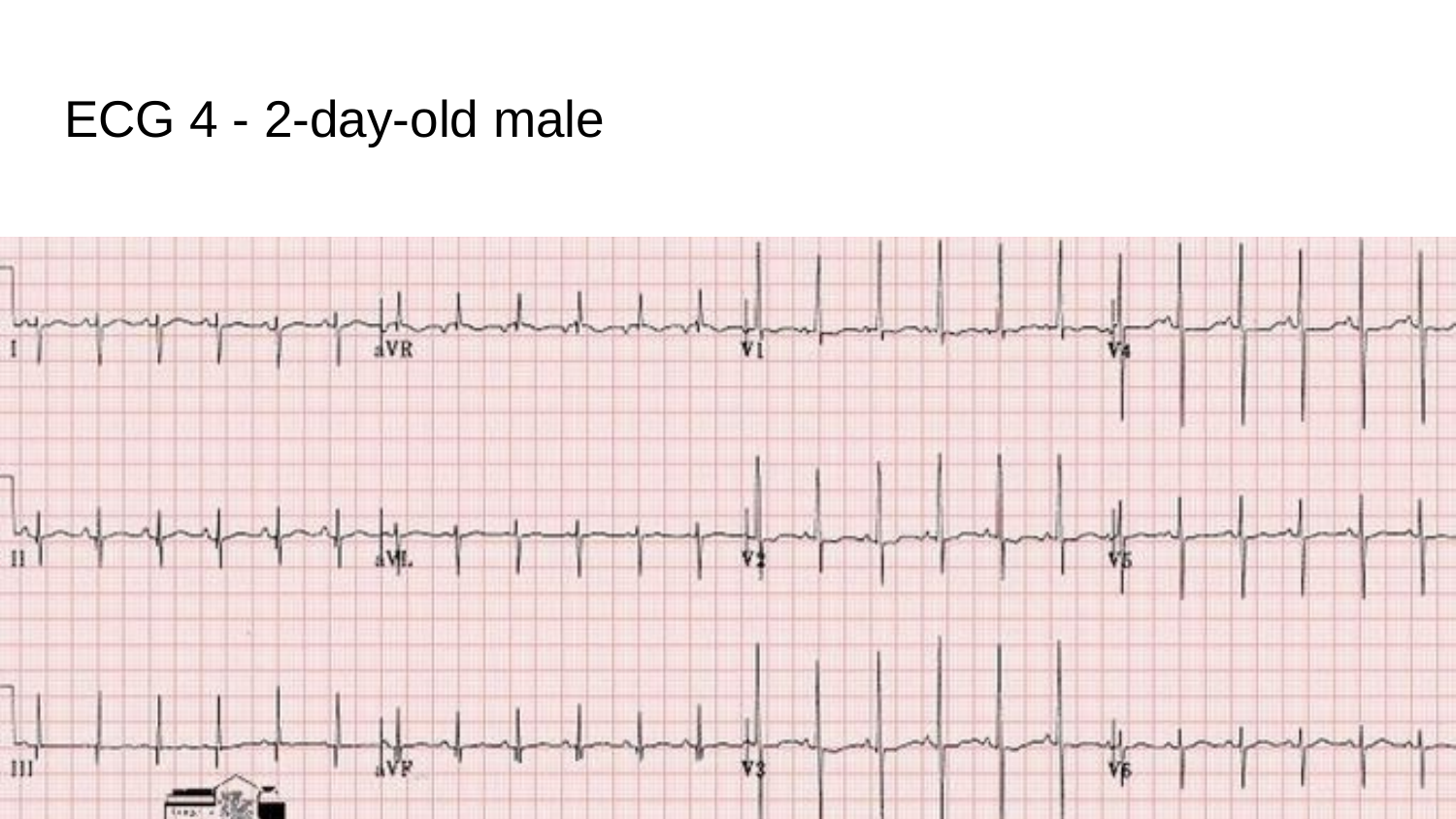

# ECG 4 - 2-day-old male

## Slide 62
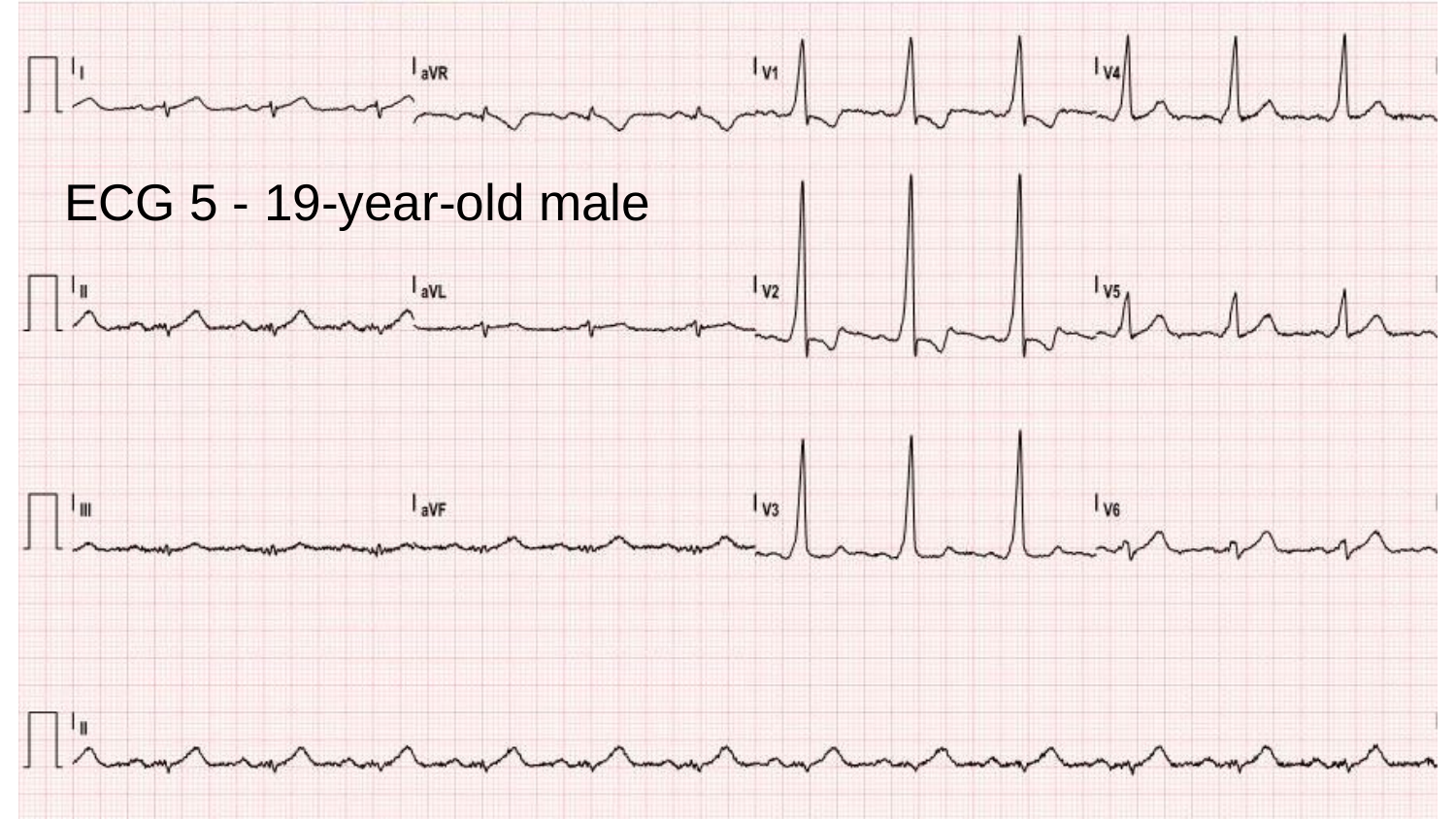

# ECG 5 - 19-year-old male

## Slide 63
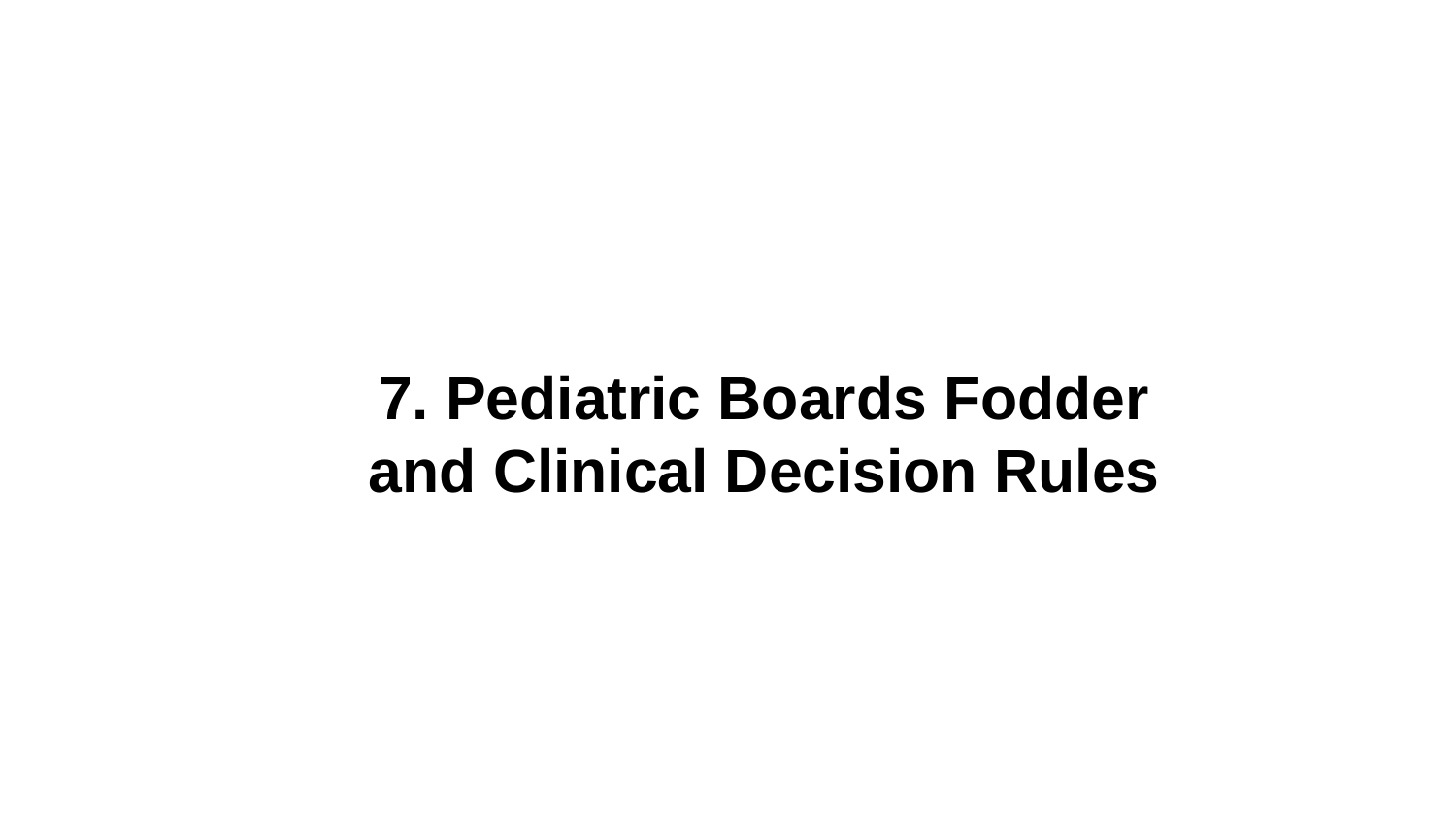

7. Pediatric Boards Fodder and Clinical Decision Rules

## Slide 64
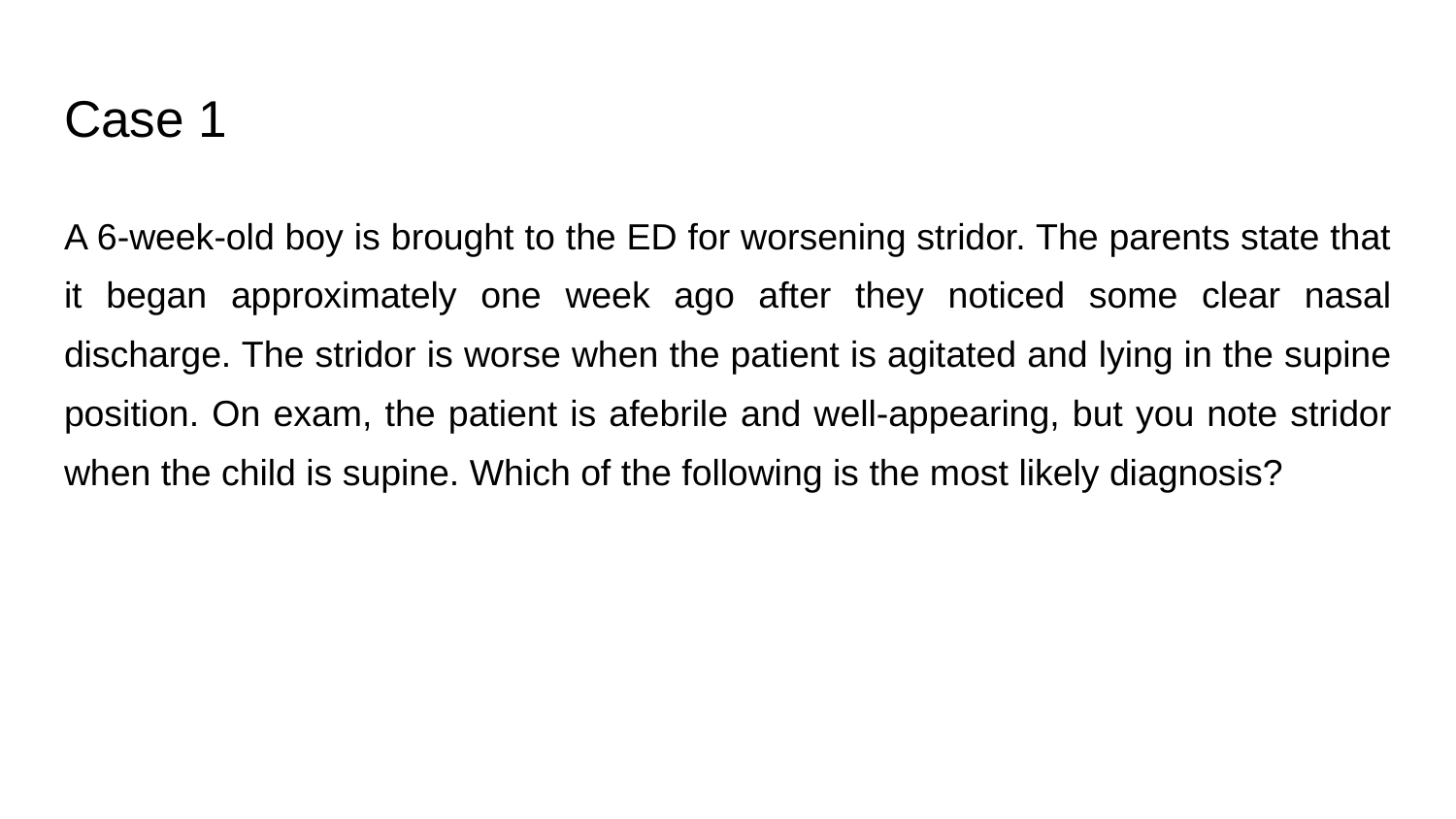

# Case 1
A 6-week-old boy is brought to the ED for worsening stridor. The parents state that it began approximately one week ago after they noticed some clear nasal discharge. The stridor is worse when the patient is agitated and lying in the supine position. On exam, the patient is afebrile and well-appearing, but you note stridor when the child is supine. Which of the following is the most likely diagnosis?

## Slide 65
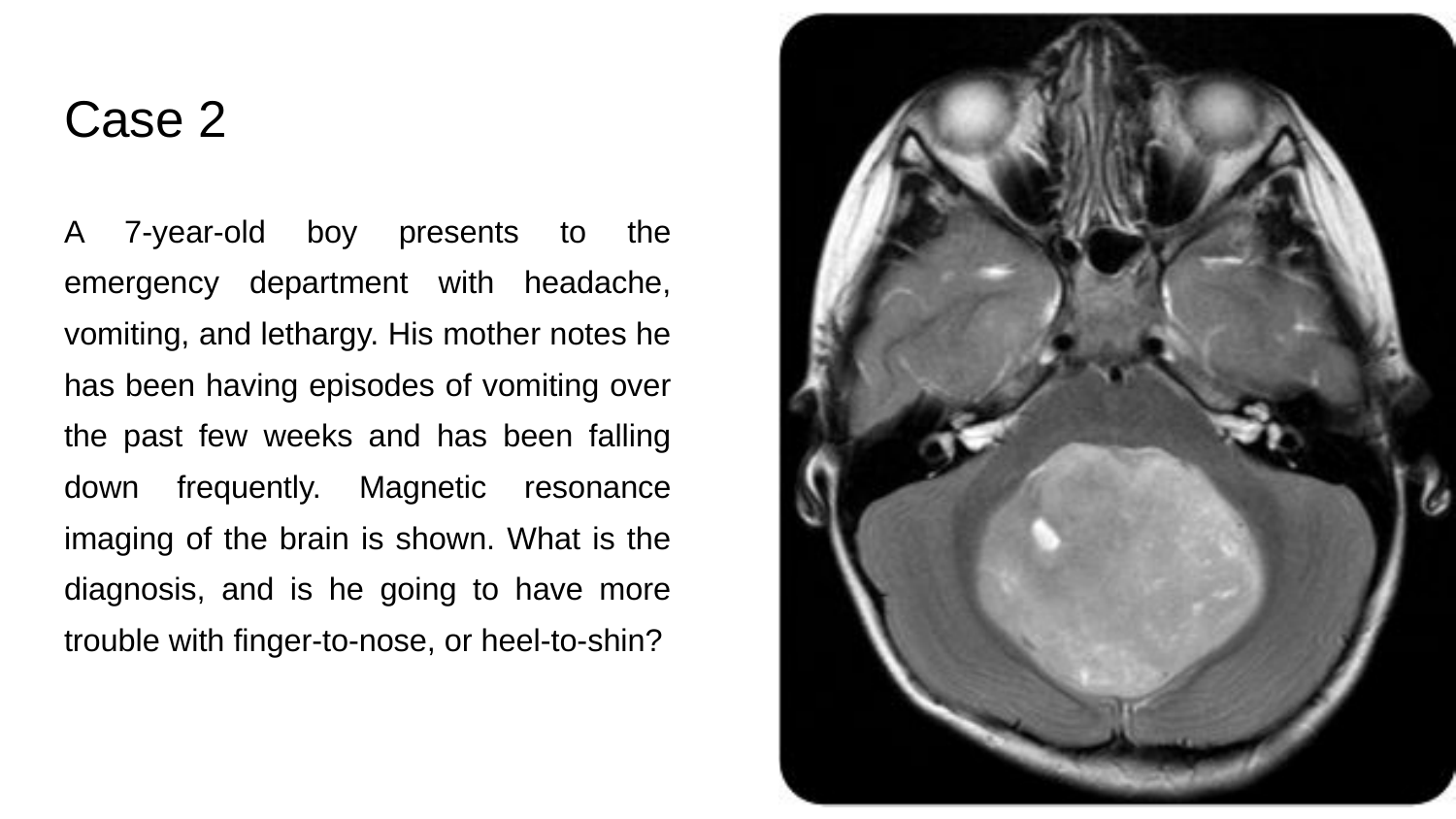

# Case 2
A 7-year-old boy presents to the emergency department with headache, vomiting, and lethargy. His mother notes he has been having episodes of vomiting over the past few weeks and has been falling down frequently. Magnetic resonance imaging of the brain is shown. What is the diagnosis, and is he going to have more trouble with finger-to-nose, or heel-to-shin?

## Slide 66
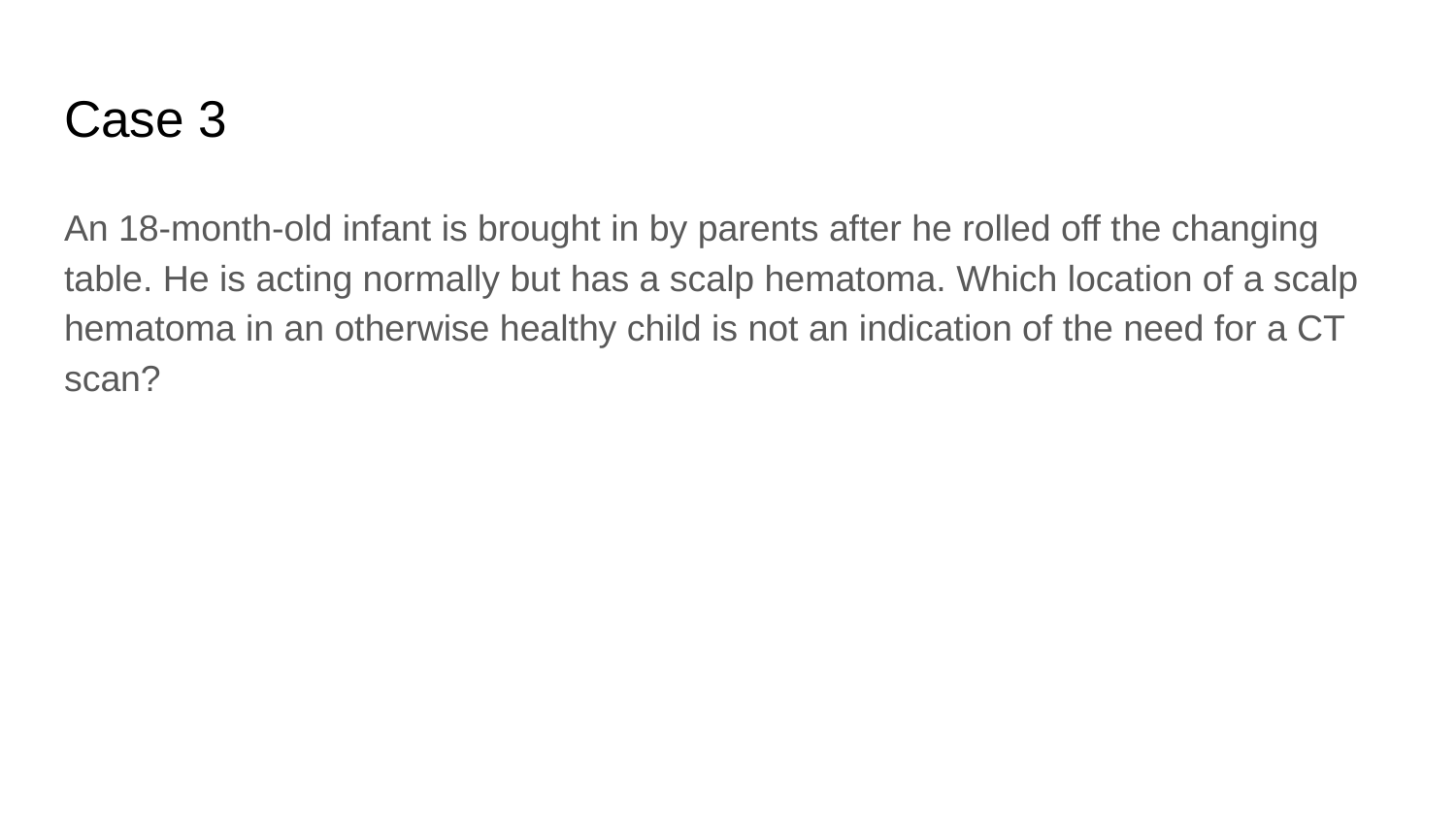

# Case 3
An 18-month-old infant is brought in by parents after he rolled off the changing table. He is acting normally but has a scalp hematoma. Which location of a scalp hematoma in an otherwise healthy child is not an indication of the need for a CT scan?

## Slide 67
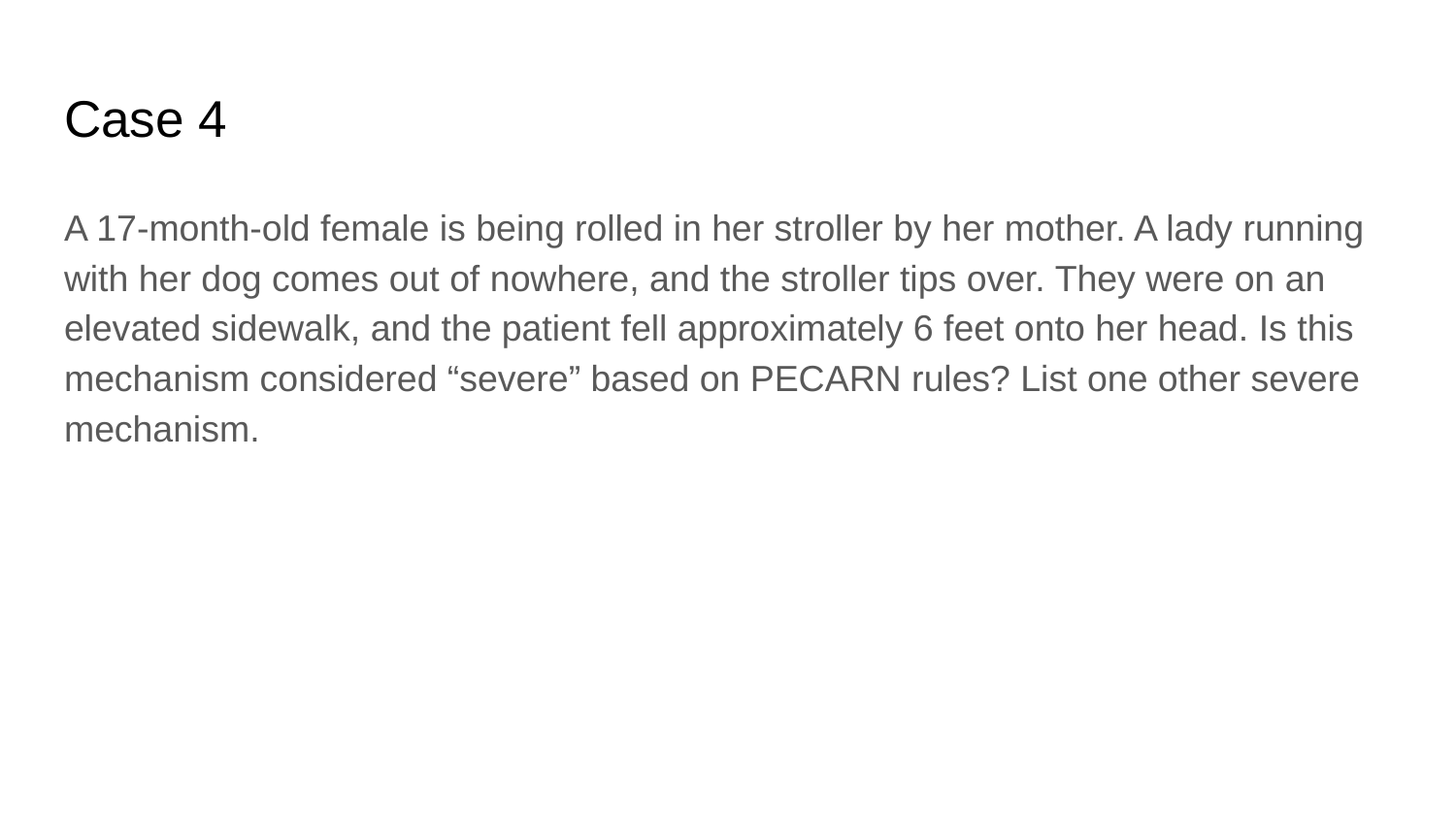

# Case 4
A 17-month-old female is being rolled in her stroller by her mother. A lady running with her dog comes out of nowhere, and the stroller tips over. They were on an elevated sidewalk, and the patient fell approximately 6 feet onto her head. Is this mechanism considered “severe” based on PECARN rules? List one other severe mechanism.

## Slide 68
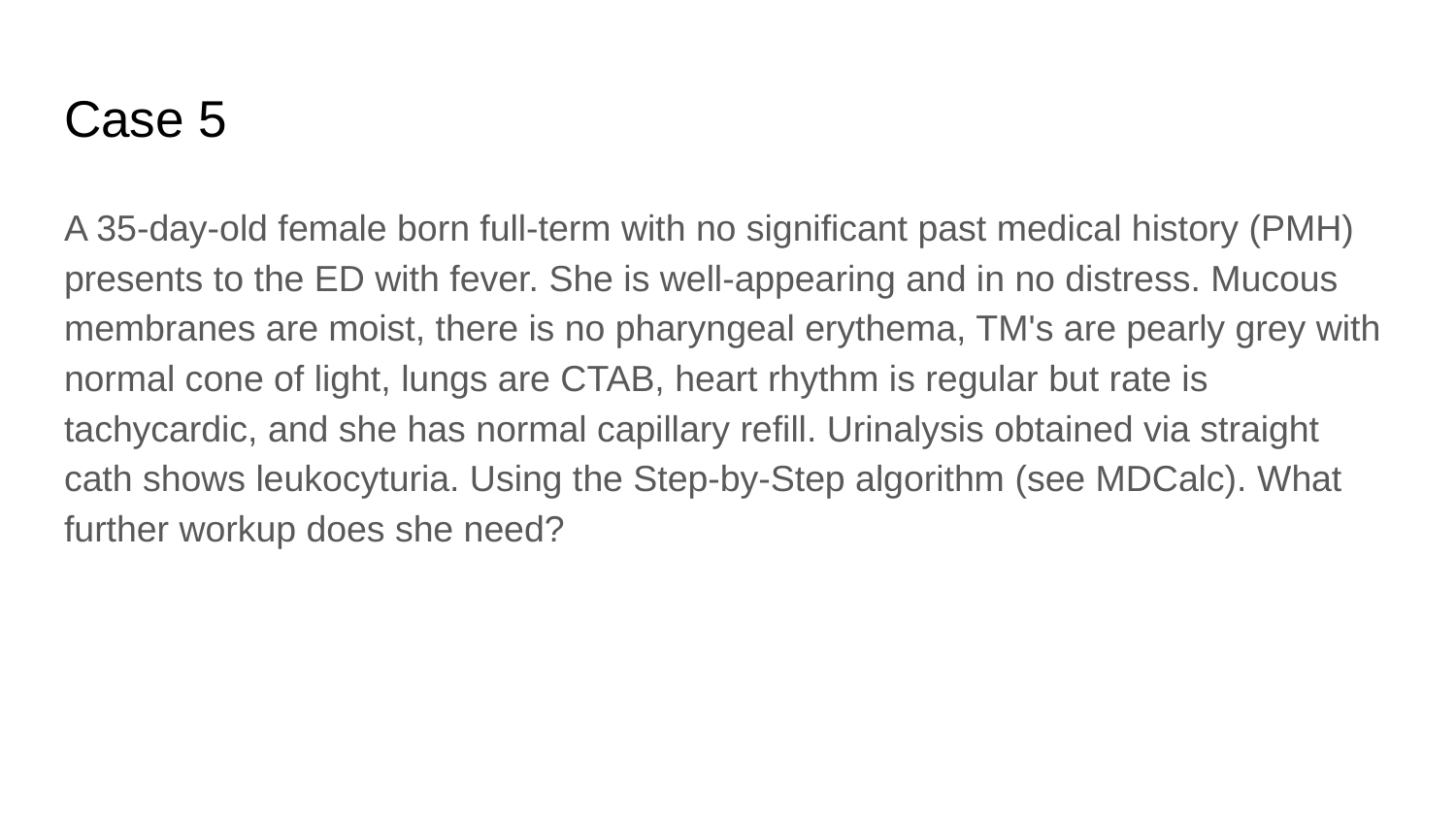

# Case 5
A 35-day-old female born full-term with no significant past medical history (PMH) presents to the ED with fever. She is well-appearing and in no distress. Mucous membranes are moist, there is no pharyngeal erythema, TM's are pearly grey with normal cone of light, lungs are CTAB, heart rhythm is regular but rate is tachycardic, and she has normal capillary refill. Urinalysis obtained via straight cath shows leukocyturia. Using the Step-by-Step algorithm (see MDCalc). What further workup does she need?

## Slide 69
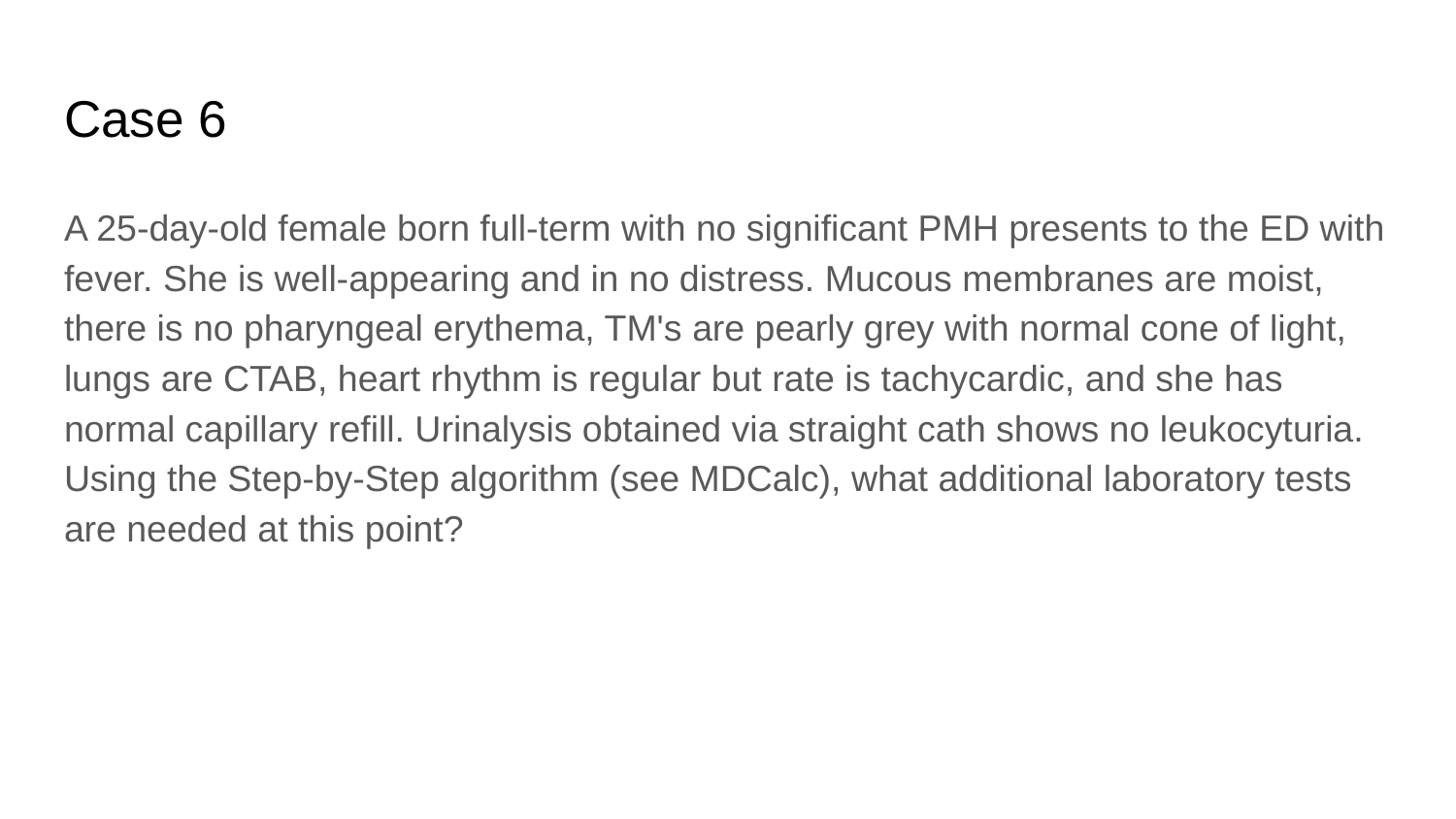

# Case 6
A 25-day-old female born full-term with no significant PMH presents to the ED with fever. She is well-appearing and in no distress. Mucous membranes are moist, there is no pharyngeal erythema, TM's are pearly grey with normal cone of light, lungs are CTAB, heart rhythm is regular but rate is tachycardic, and she has normal capillary refill. Urinalysis obtained via straight cath shows no leukocyturia. Using the Step-by-Step algorithm (see MDCalc), what additional laboratory tests are needed at this point?

## Slide 70
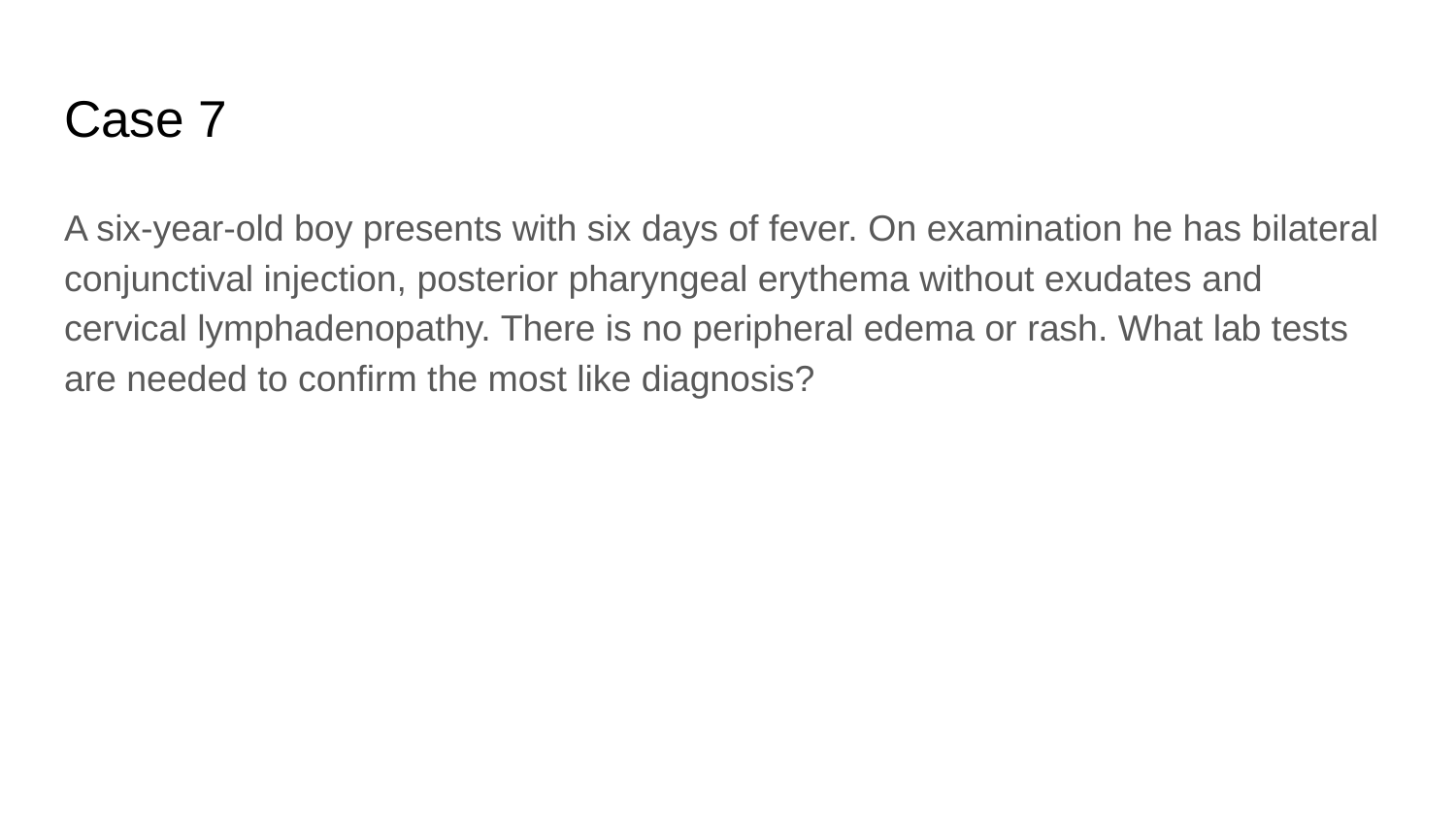

# Case 7
A six-year-old boy presents with six days of fever. On examination he has bilateral conjunctival injection, posterior pharyngeal erythema without exudates and cervical lymphadenopathy. There is no peripheral edema or rash. What lab tests are needed to confirm the most like diagnosis?

## Slide 71
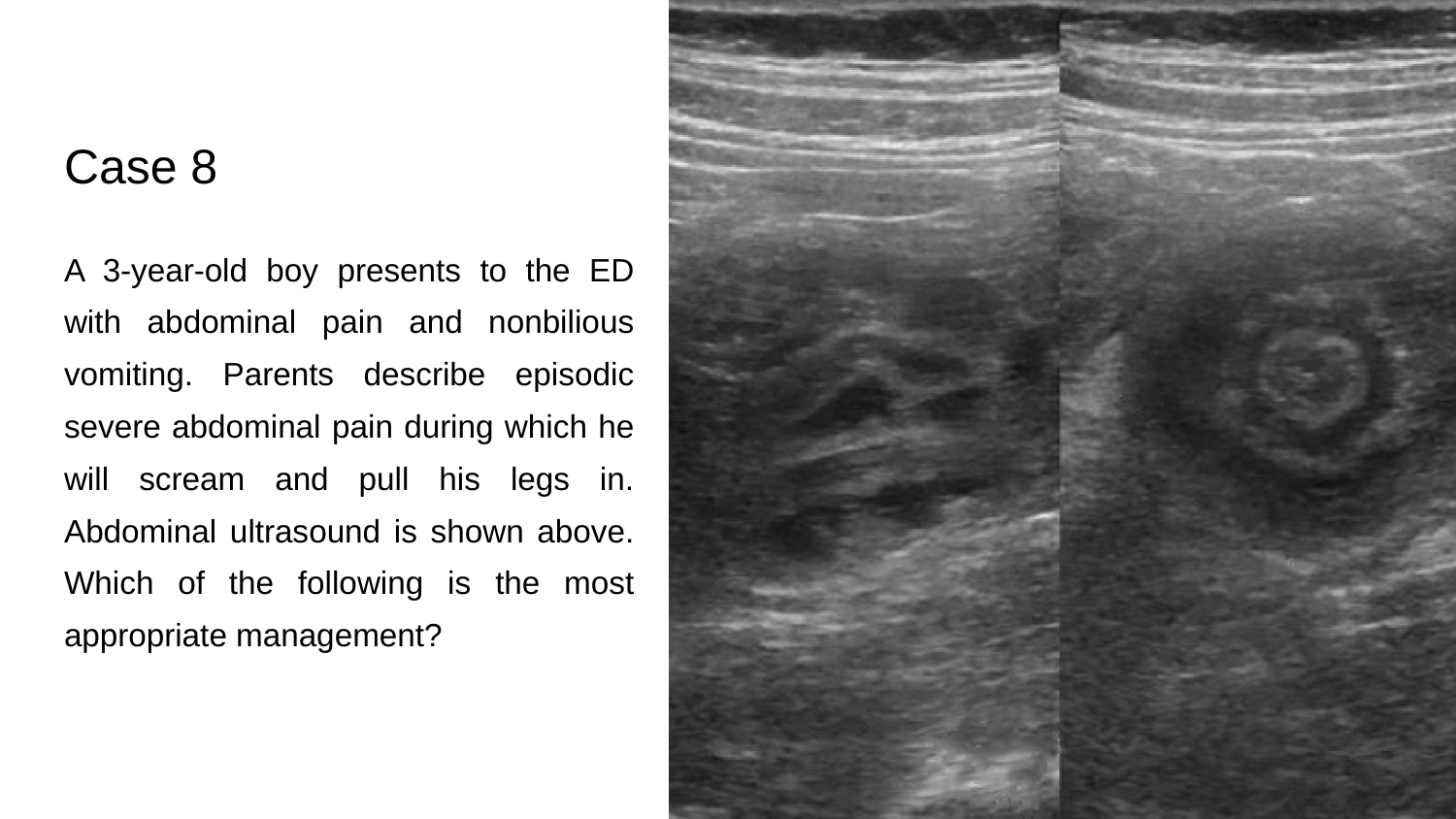

# Case 8
A 3-year-old boy presents to the ED with abdominal pain and nonbilious vomiting. Parents describe episodic severe abdominal pain during which he will scream and pull his legs in. Abdominal ultrasound is shown above. Which of the following is the most appropriate management?

## Slide 72
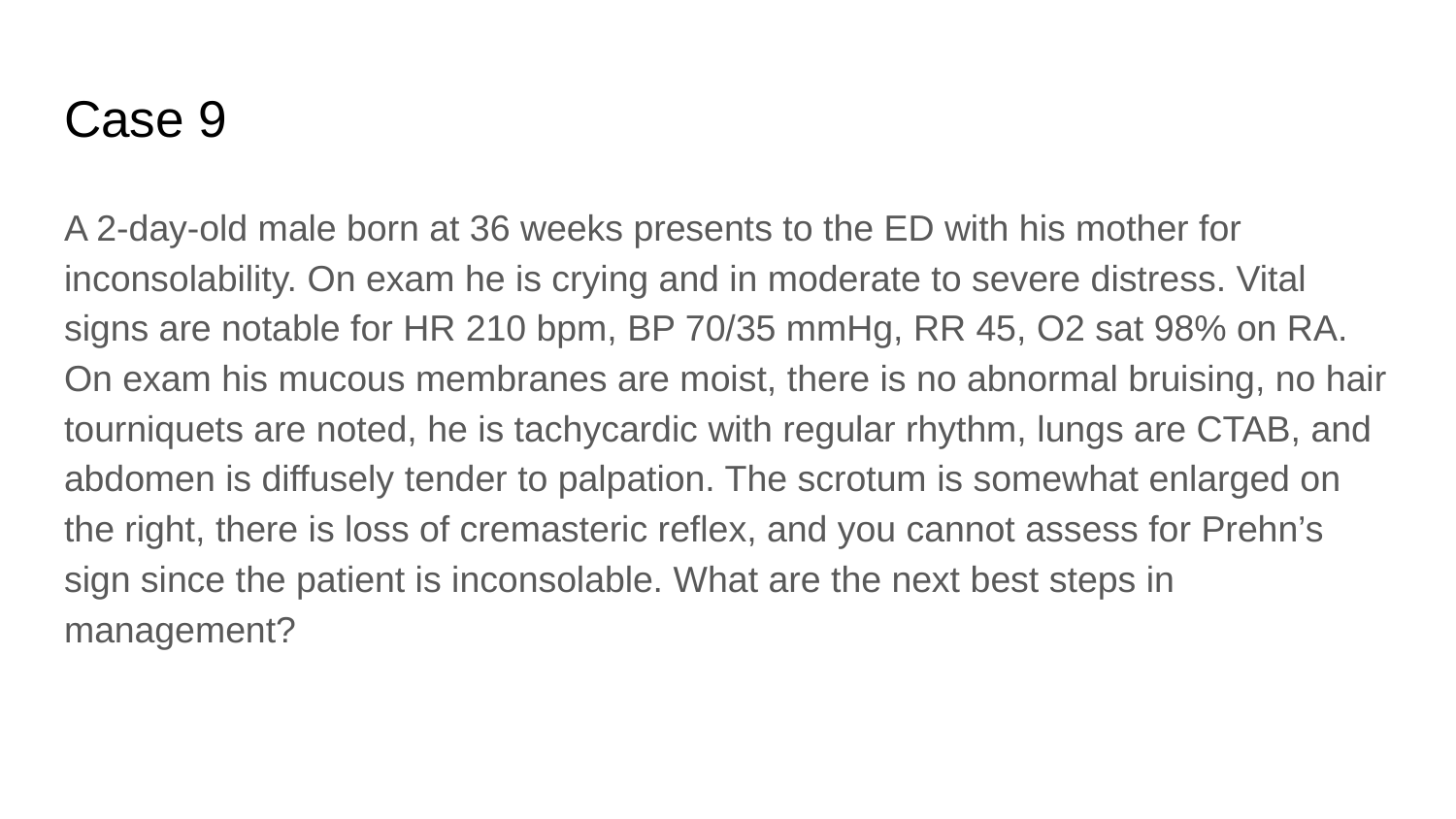

# Case 9
A 2-day-old male born at 36 weeks presents to the ED with his mother for inconsolability. On exam he is crying and in moderate to severe distress. Vital signs are notable for HR 210 bpm, BP 70/35 mmHg, RR 45, O2 sat 98% on RA. On exam his mucous membranes are moist, there is no abnormal bruising, no hair tourniquets are noted, he is tachycardic with regular rhythm, lungs are CTAB, and abdomen is diffusely tender to palpation. The scrotum is somewhat enlarged on the right, there is loss of cremasteric reflex, and you cannot assess for Prehn’s sign since the patient is inconsolable. What are the next best steps in management?

## Slide 73
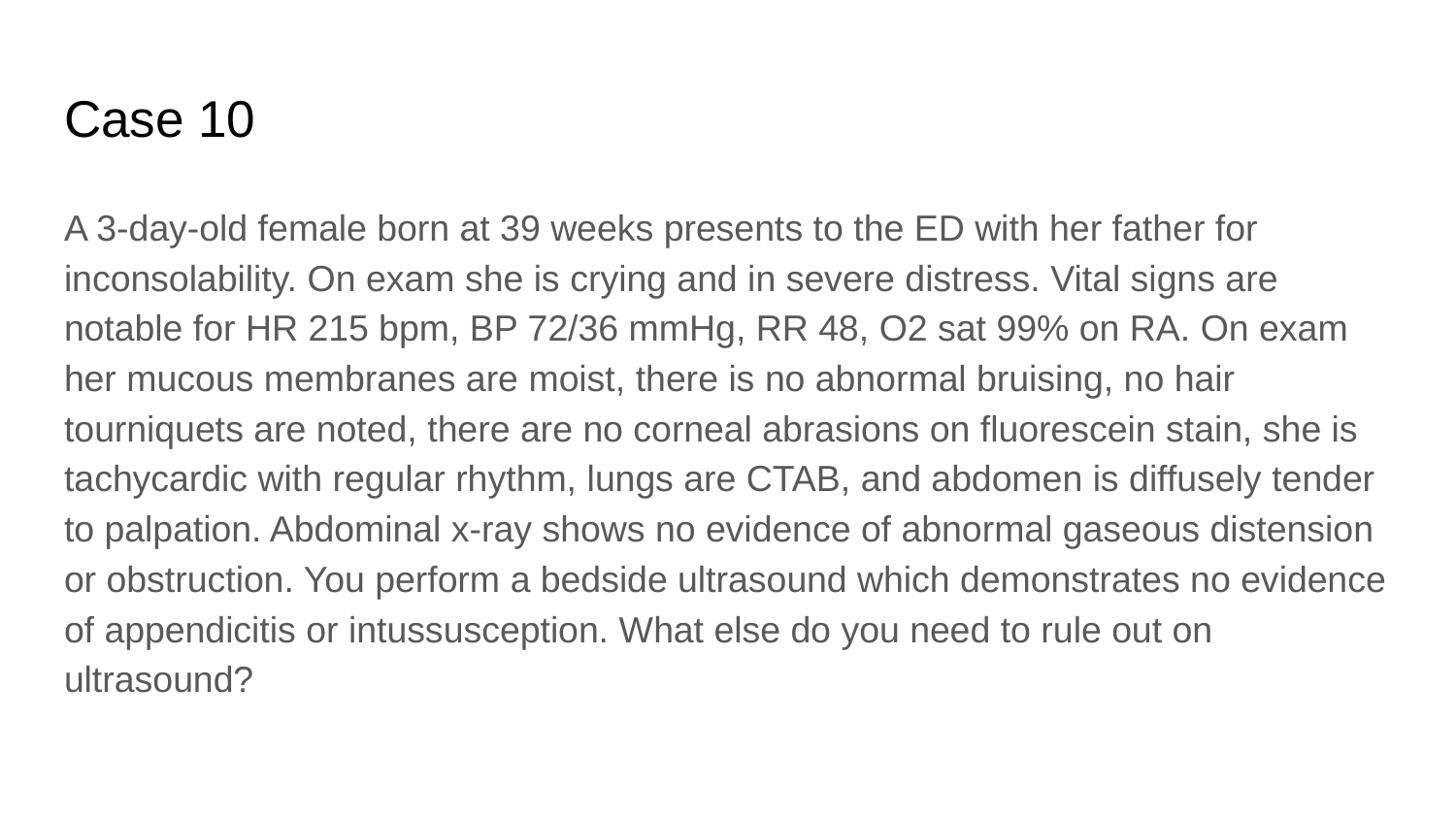

# Case 10
A 3-day-old female born at 39 weeks presents to the ED with her father for inconsolability. On exam she is crying and in severe distress. Vital signs are notable for HR 215 bpm, BP 72/36 mmHg, RR 48, O2 sat 99% on RA. On exam her mucous membranes are moist, there is no abnormal bruising, no hair tourniquets are noted, there are no corneal abrasions on fluorescein stain, she is tachycardic with regular rhythm, lungs are CTAB, and abdomen is diffusely tender to palpation. Abdominal x-ray shows no evidence of abnormal gaseous distension or obstruction. You perform a bedside ultrasound which demonstrates no evidence of appendicitis or intussusception. What else do you need to rule out on ultrasound?

## Slide 74
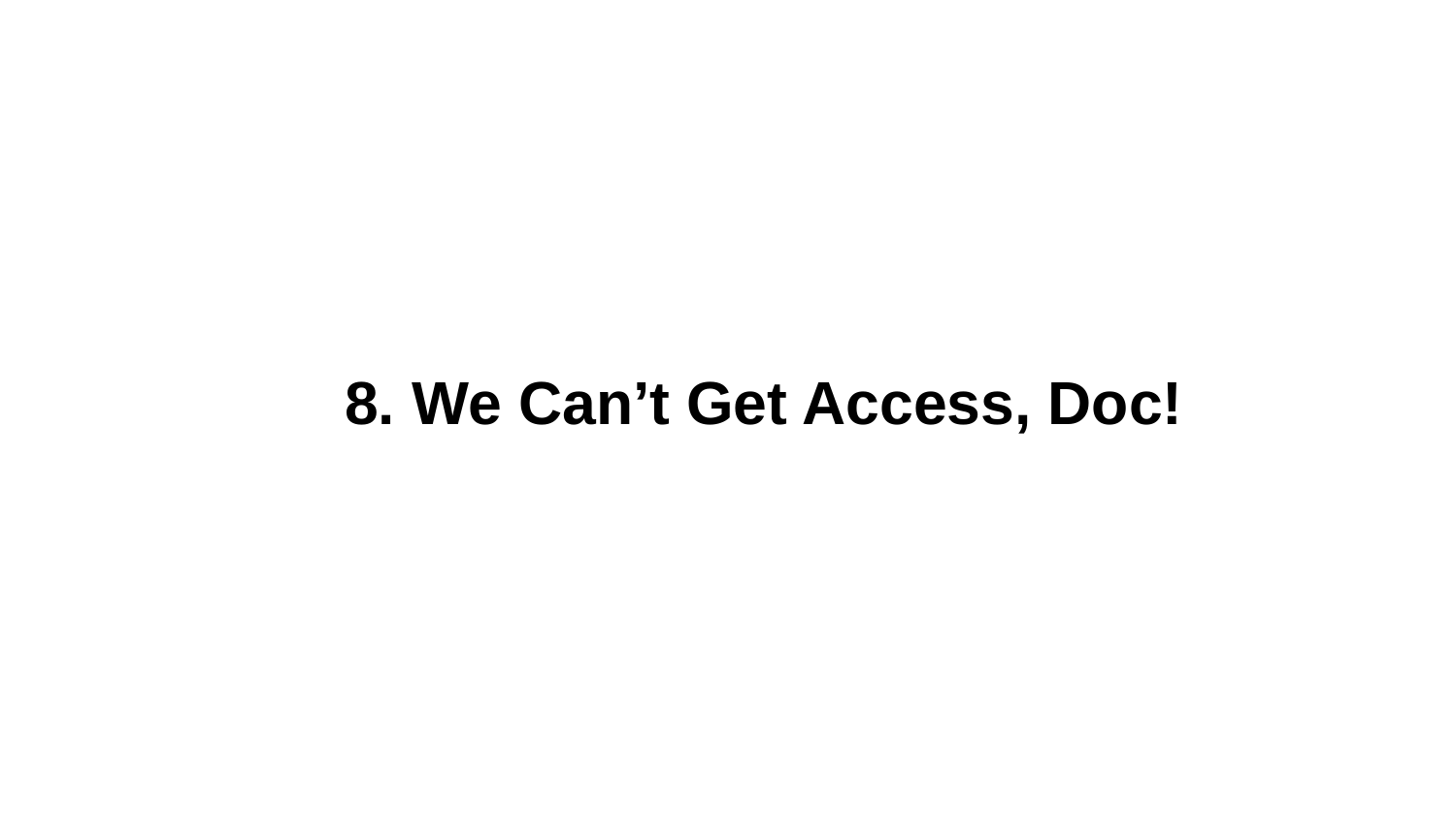

8. We Can’t Get Access, Doc!

## Slide 75
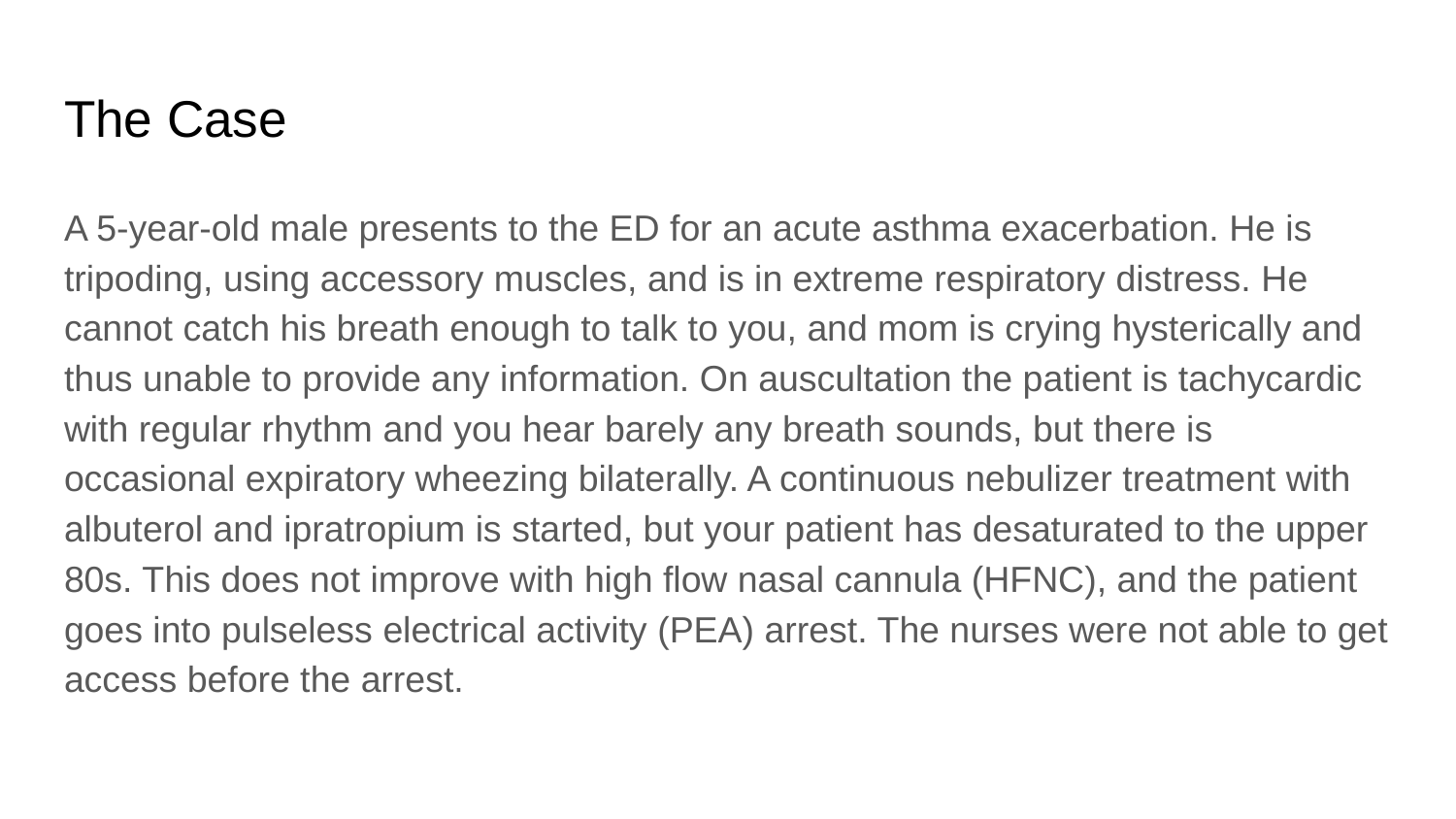

# The Case
A 5-year-old male presents to the ED for an acute asthma exacerbation. He is tripoding, using accessory muscles, and is in extreme respiratory distress. He cannot catch his breath enough to talk to you, and mom is crying hysterically and thus unable to provide any information. On auscultation the patient is tachycardic with regular rhythm and you hear barely any breath sounds, but there is occasional expiratory wheezing bilaterally. A continuous nebulizer treatment with albuterol and ipratropium is started, but your patient has desaturated to the upper 80s. This does not improve with high flow nasal cannula (HFNC), and the patient goes into pulseless electrical activity (PEA) arrest. The nurses were not able to get access before the arrest.

## Slide 76
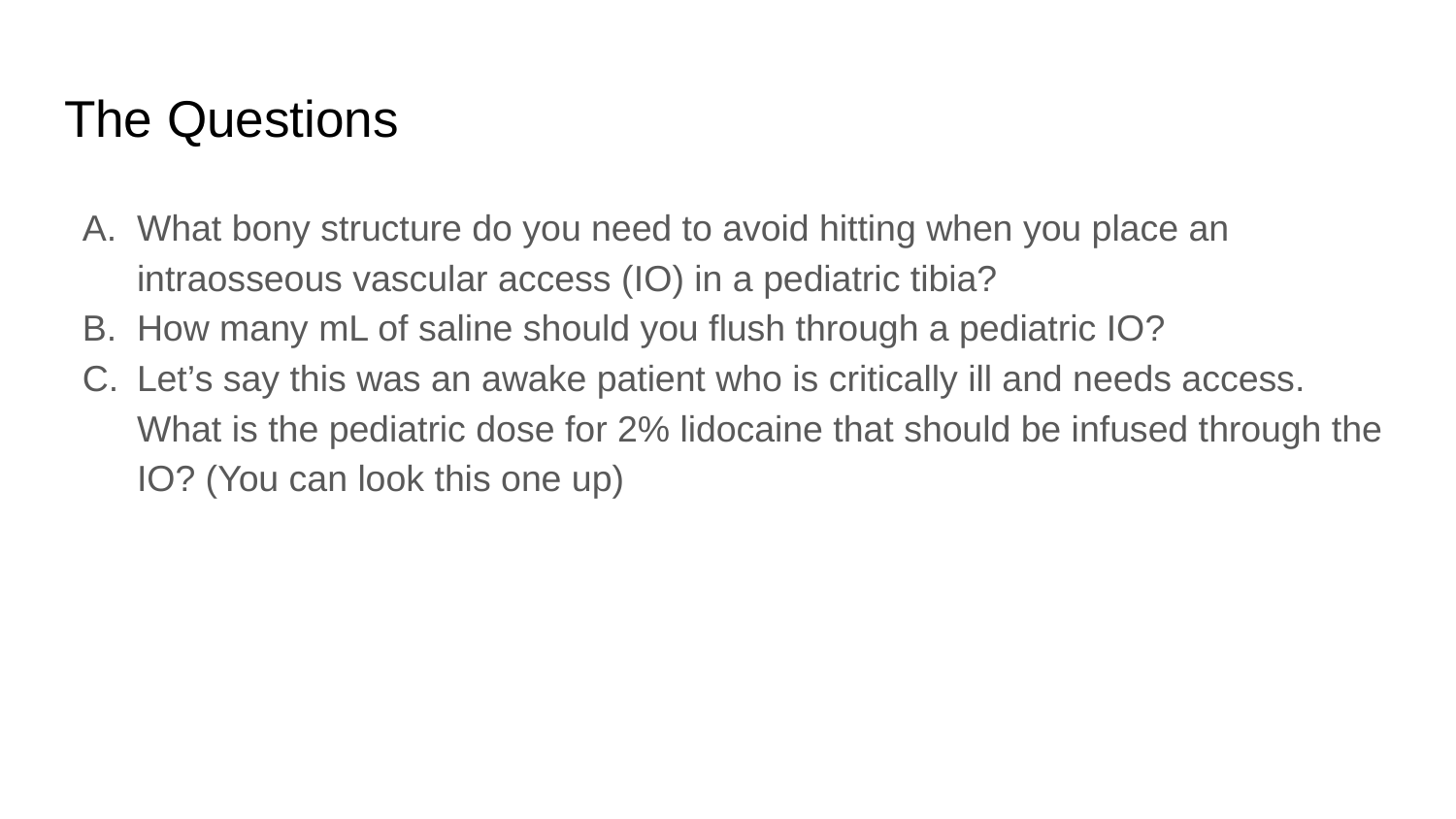

# The Questions
What bony structure do you need to avoid hitting when you place an intraosseous vascular access (IO) in a pediatric tibia?
How many mL of saline should you flush through a pediatric IO?
Let’s say this was an awake patient who is critically ill and needs access. What is the pediatric dose for 2% lidocaine that should be infused through the IO? (You can look this one up)

## Slide 77
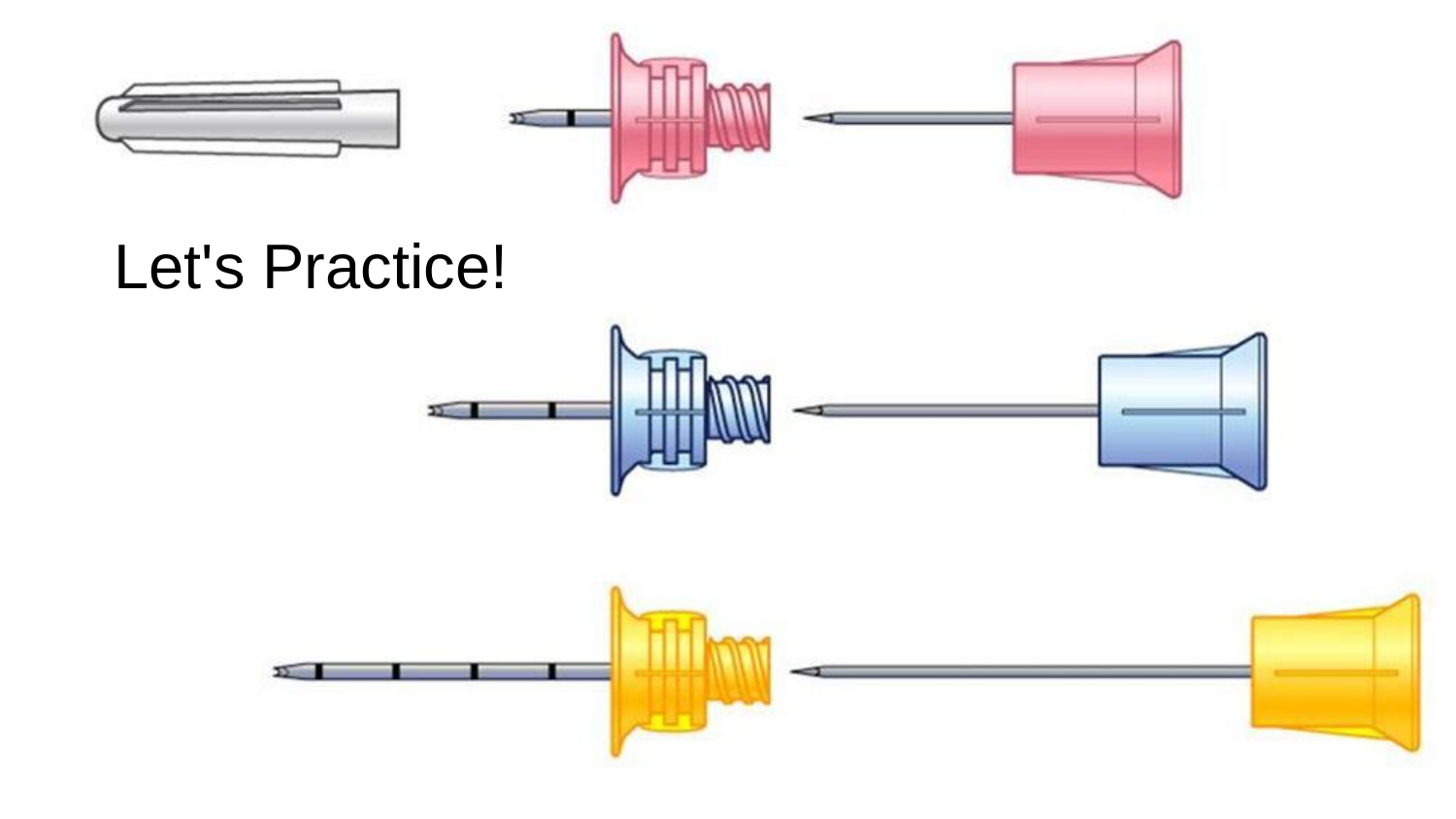

# Let's Practice!

## Slide 78
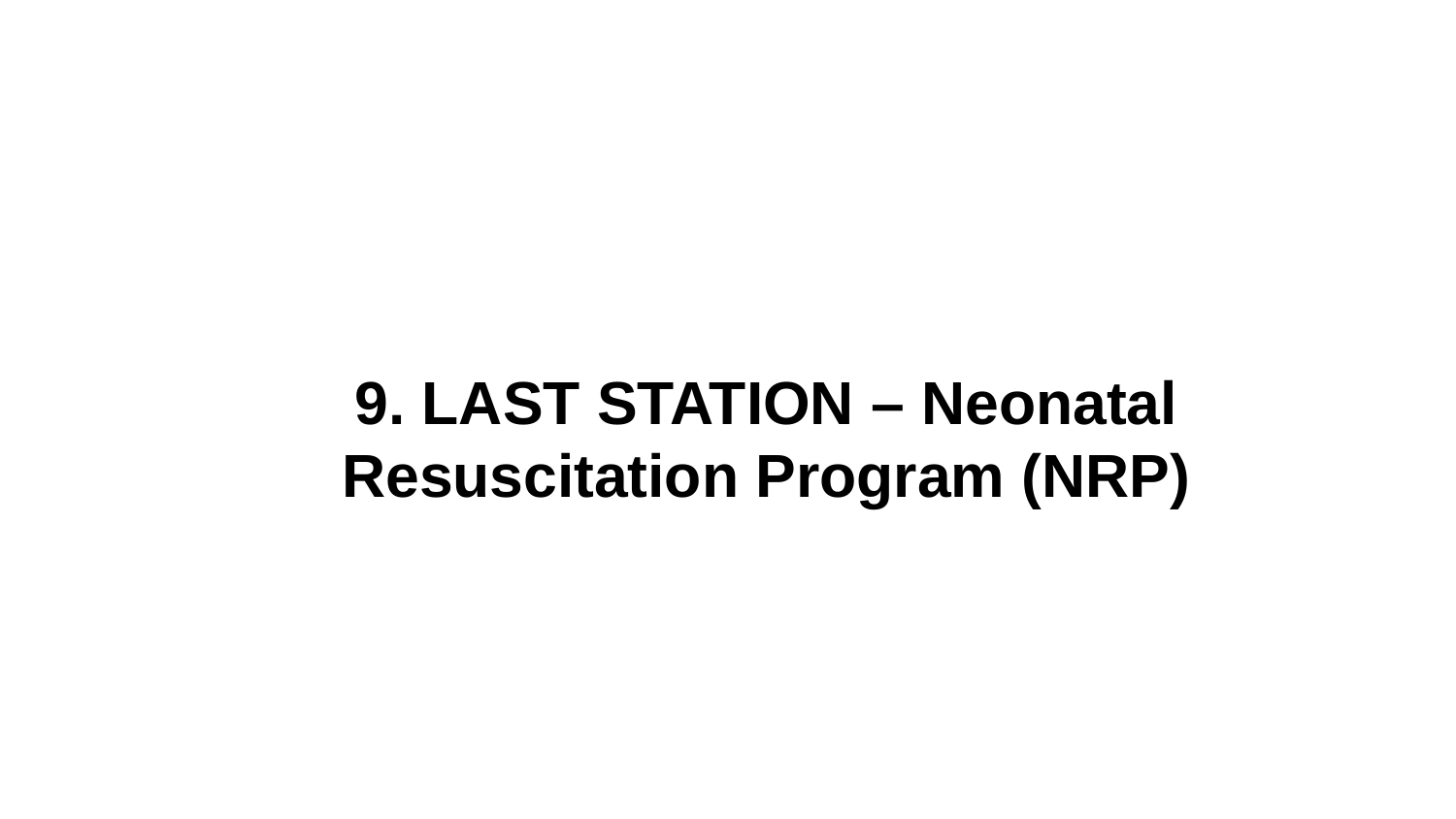

9. LAST STATION – Neonatal Resuscitation Program (NRP)

## Slide 79
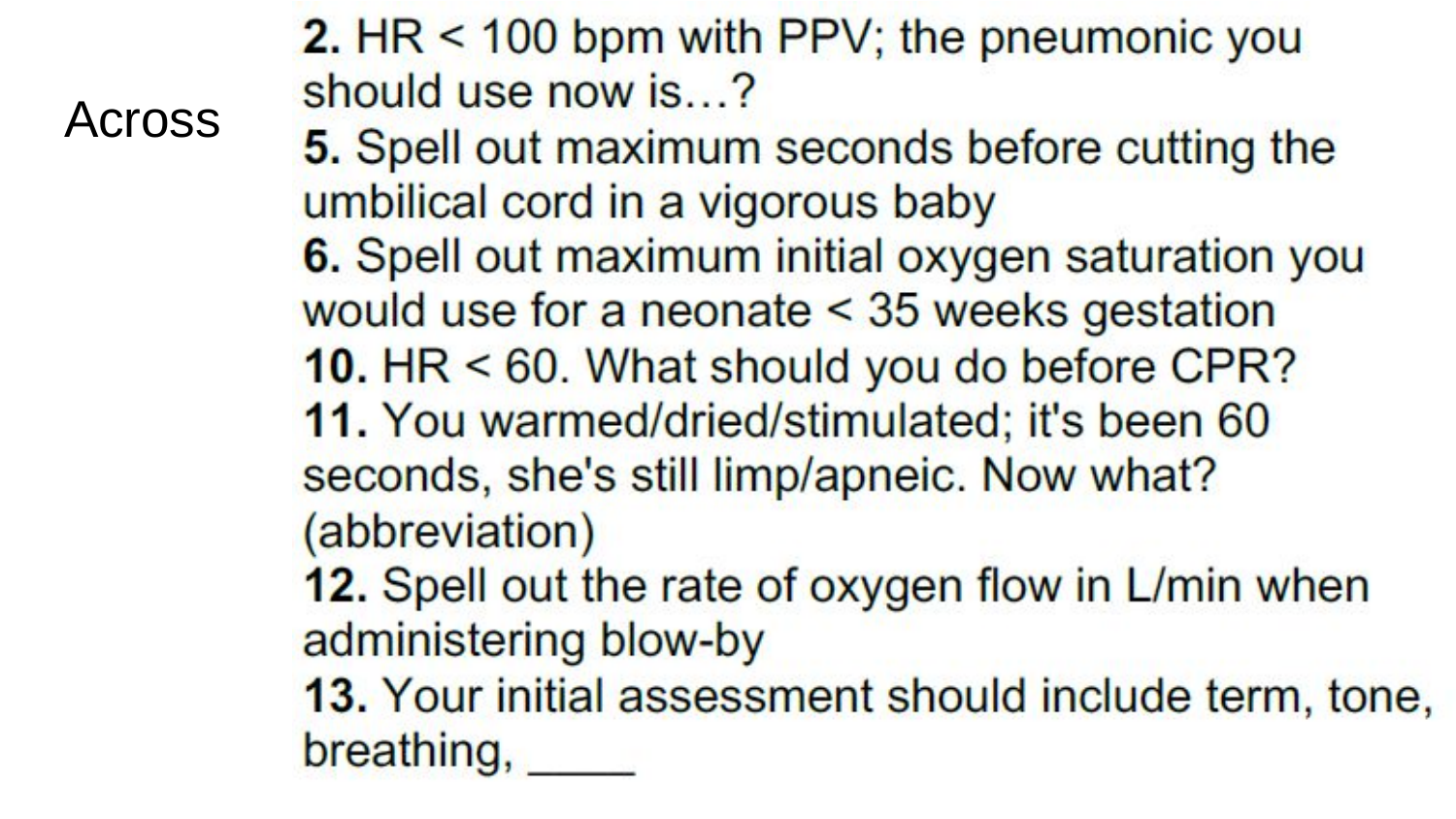

# Across

## Slide 80
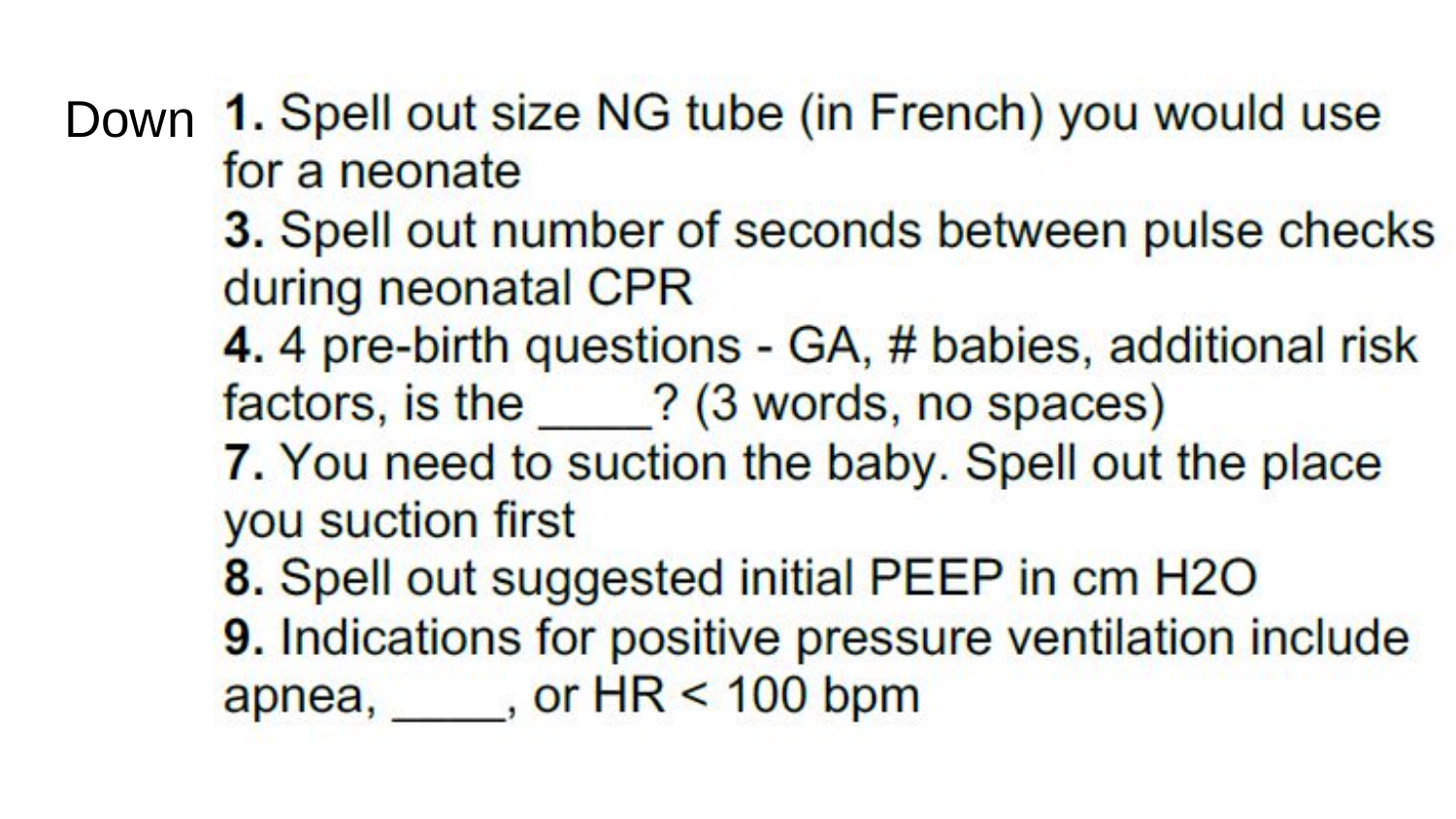

# Down

## Slide 81
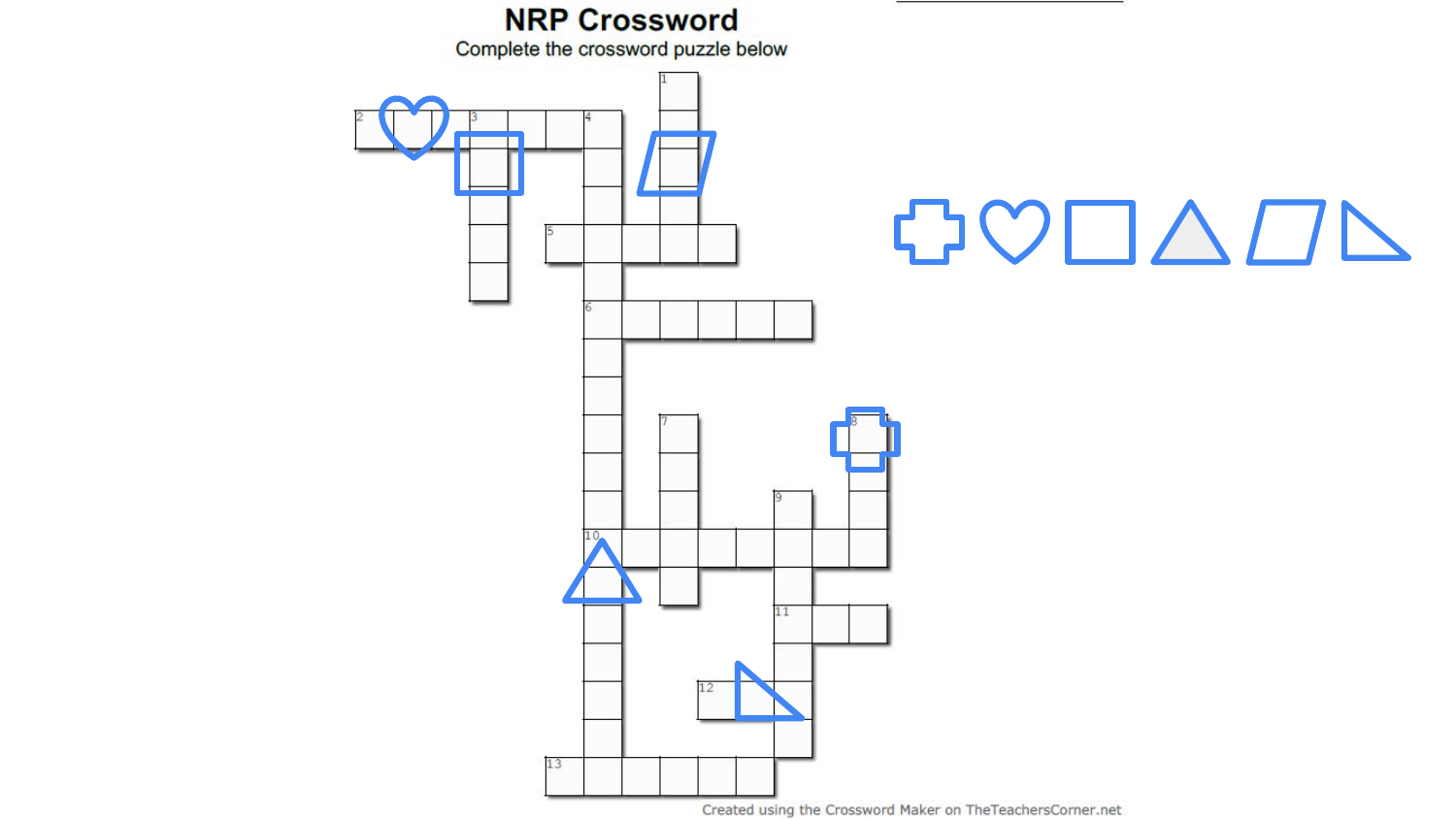

## Slide 82
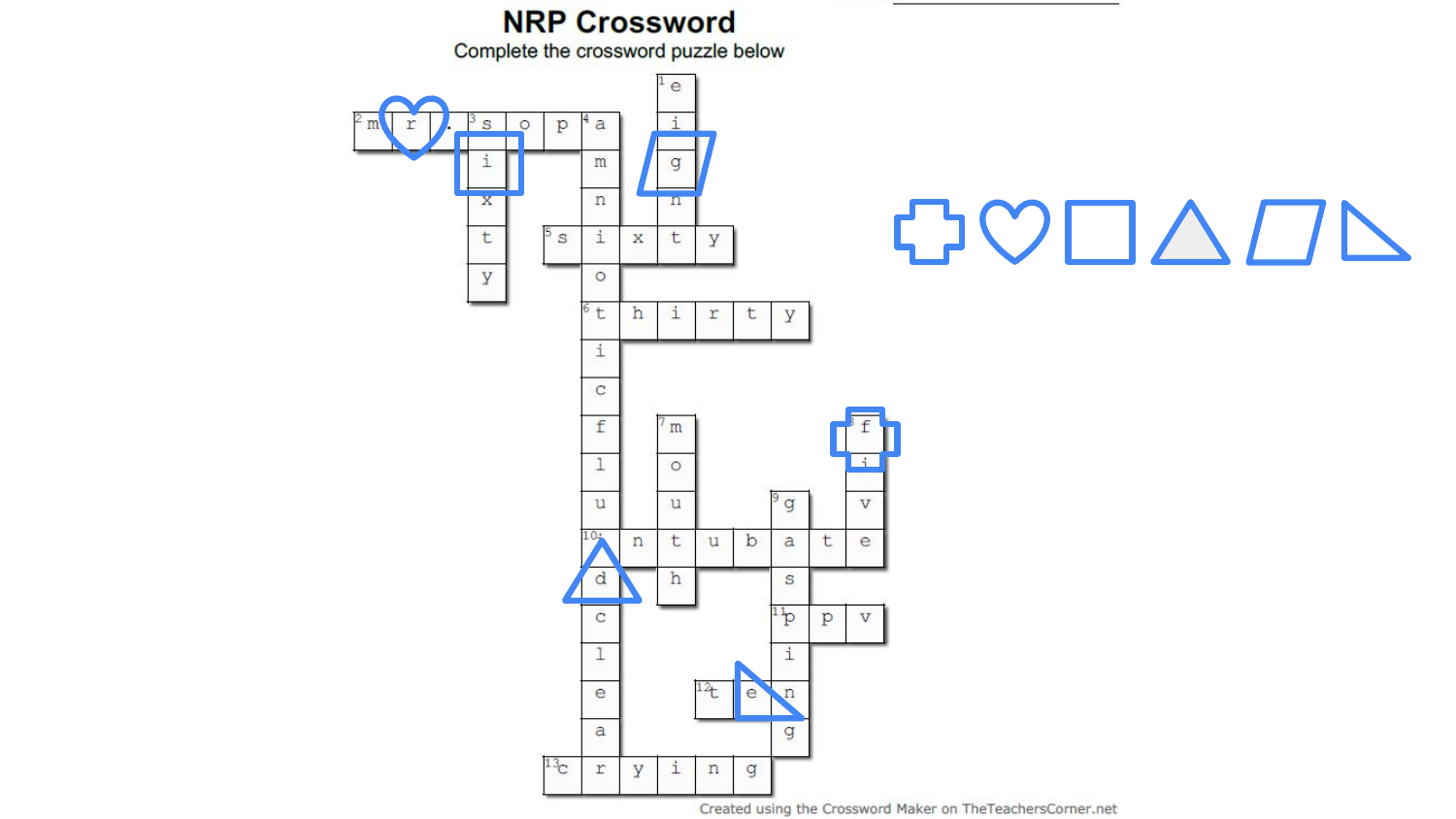

## Slide 83
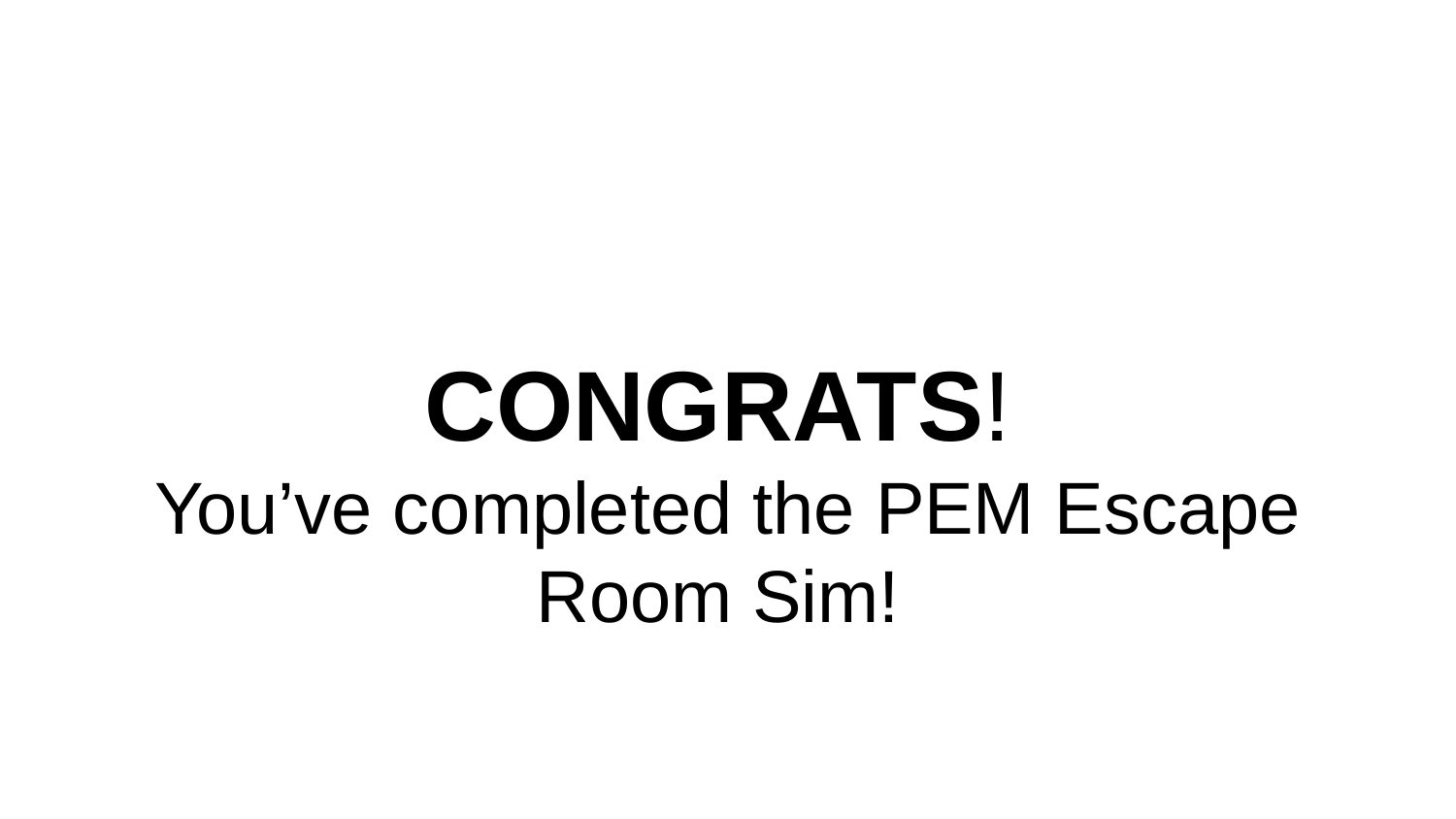

# CONGRATS!
You’ve completed the PEM Escape Room Sim!

## Slide 84
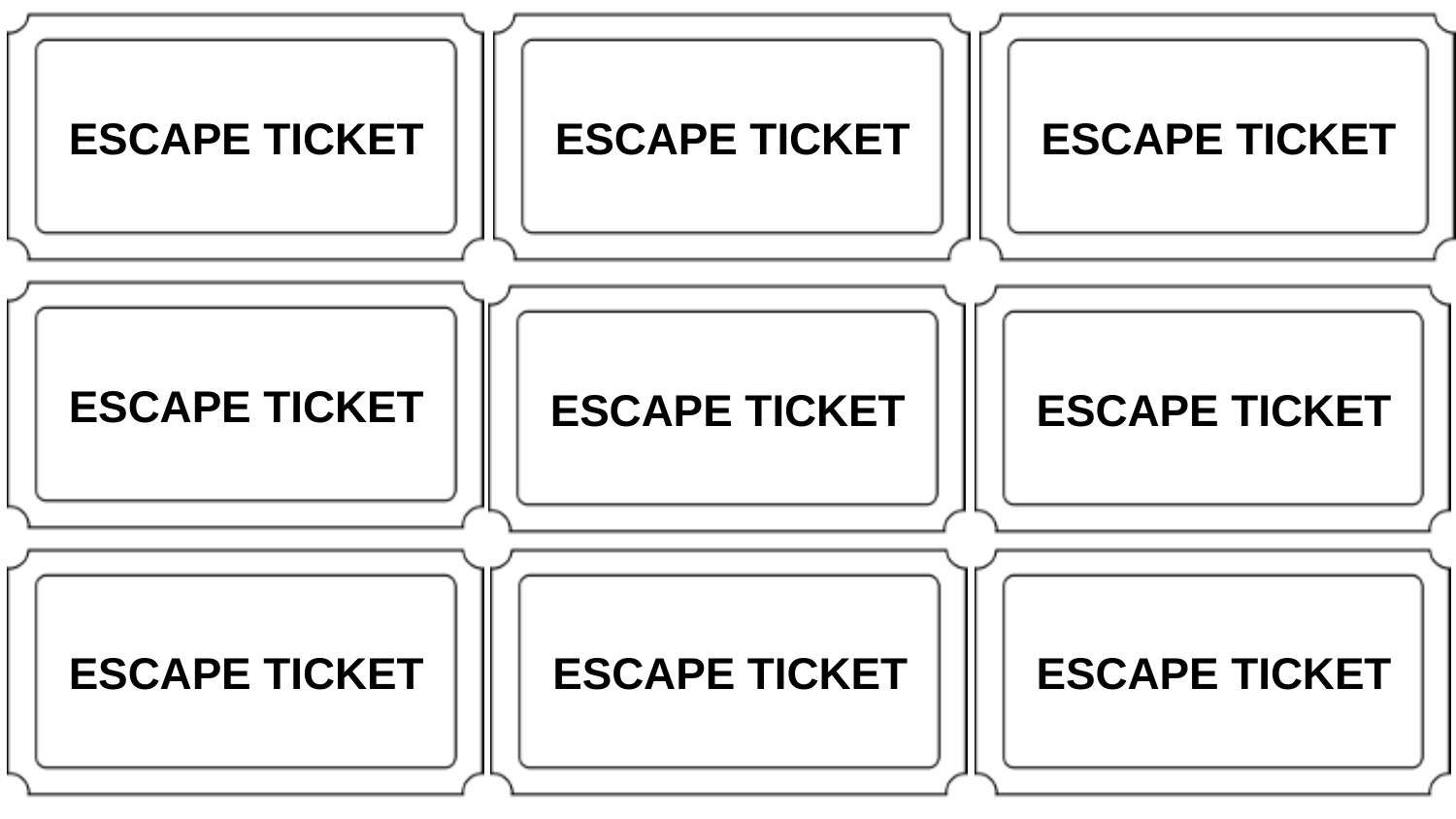

ESCAPE TICKET
ESCAPE TICKET
ESCAPE TICKET
ESCAPE TICKET
ESCAPE TICKET
ESCAPE TICKET
ESCAPE TICKET
ESCAPE TICKET
ESCAPE TICKET
