# Supplementary material for: Little Patients, Big Tasks - A Pediatric Emergency Medicine Escape Room
Source: J Educ Teach Emerg Med. 2023 Oct 31;8(4):SG1–SG19. doi: 10.21980/J89W70 (PMC10631808; doi:10.21980/J89W70)
Supplement: Supplementary file 2 [file jetem-8-4-sg1-supp2.pptx]

## Slide 1
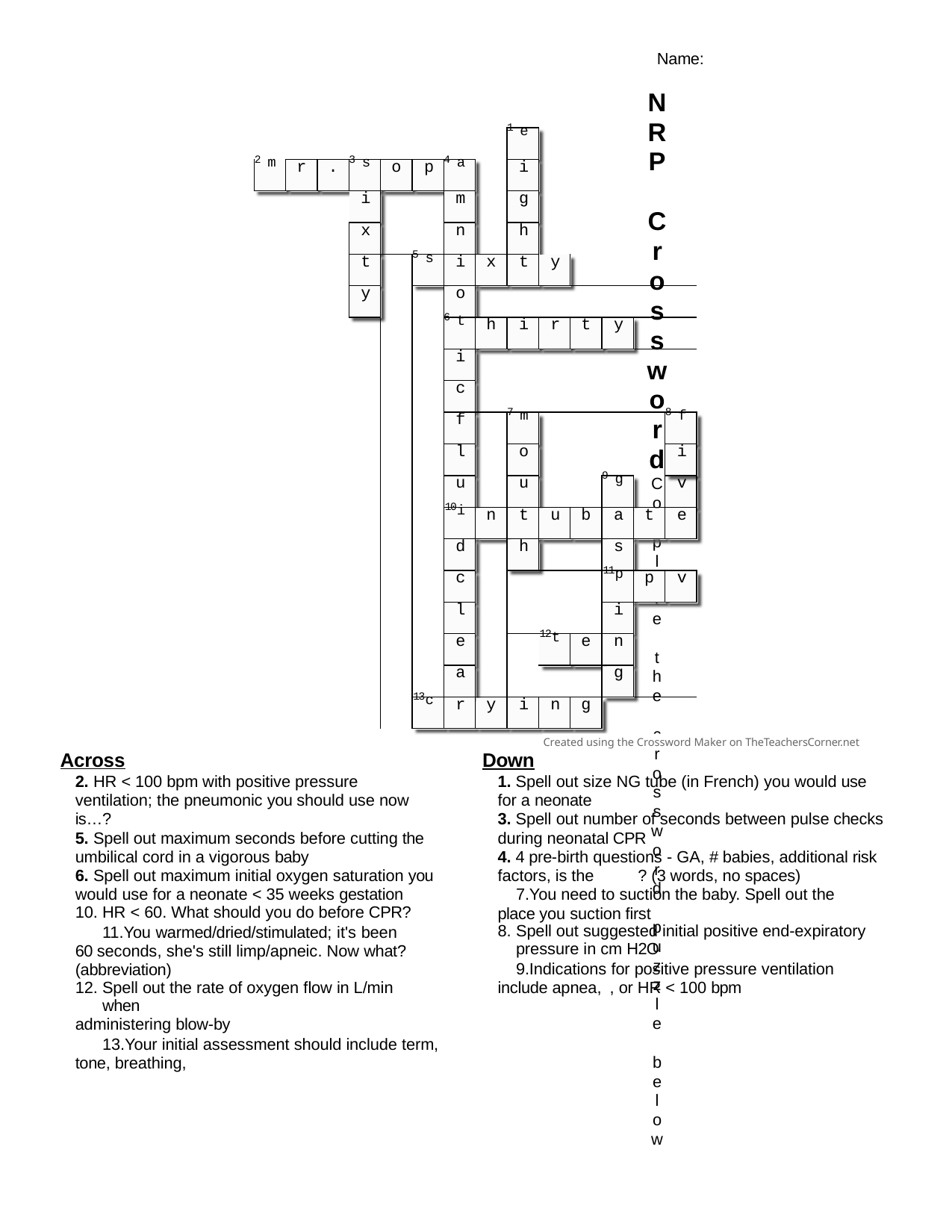

Name:
NRP Crossword
Complete the crossword puzzle below
| | | | | | | | | 1 e | | | | | |
| --- | --- | --- | --- | --- | --- | --- | --- | --- | --- | --- | --- | --- | --- |
| 2 m | r | . | 3 s | o | p | 4 a | | i | | | | | |
| | | | i | | | m | | g | | | | | |
| | | | x | | | n | | h | | | | | |
| | | | t | | 5 s | i | x | t | y | | | | |
| | | | y | | | o | | | | | | | |
| | | | | | | 6 t | h | i | r | t | y | | |
| | | | | | | i | | | | | | | |
| | | | | | | c | | | | | | | |
| | | | | | | f | | 7 m | | | | | 8 f |
| | | | | | | l | | o | | | | | i |
| | | | | | | u | | u | | | 9 g | | v |
| | | | | | | 10i | n | t | u | b | a | t | e |
| | | | | | | d | | h | | | s | | |
| | | | | | | c | | | | | 11p | p | v |
| | | | | | | l | | | | | i | | |
| | | | | | | e | | | 12t | e | n | | |
| | | | | | | a | | | | | g | | |
| | | | | | 13c | r | y | i | n | g | | | |
Created using the Crossword Maker on TheTeachersCorner.net
Across
2. HR < 100 bpm with positive pressure ventilation; the pneumonic you should use now is…?
5. Spell out maximum seconds before cutting the umbilical cord in a vigorous baby
6. Spell out maximum initial oxygen saturation you would use for a neonate < 35 weeks gestation
HR < 60. What should you do before CPR?
You warmed/dried/stimulated; it's been 60 seconds, she's still limp/apneic. Now what? (abbreviation)
Spell out the rate of oxygen flow in L/min when
administering blow-by
Your initial assessment should include term, tone, breathing,
Down
1. Spell out size NG tube (in French) you would use for a neonate
3. Spell out number of seconds between pulse checks during neonatal CPR
4. 4 pre-birth questions - GA, # babies, additional risk factors, is the 	? (3 words, no spaces)
You need to suction the baby. Spell out the place you suction first
Spell out suggested initial positive end-expiratory pressure in cm H2O
Indications for positive pressure ventilation include apnea, 	, or HR < 100 bpm
