# Supplementary material for: Little Patients, Big Tasks - A Pediatric Emergency Medicine Escape Room
Source: J Educ Teach Emerg Med. 2023 Oct 31;8(4):SG1–SG19. doi: 10.21980/J89W70 (PMC10631808; doi:10.21980/J89W70)
Supplement: Supplementary file 3 [file jetem-8-4-sg1-supp3.pptx]

## Slide 1
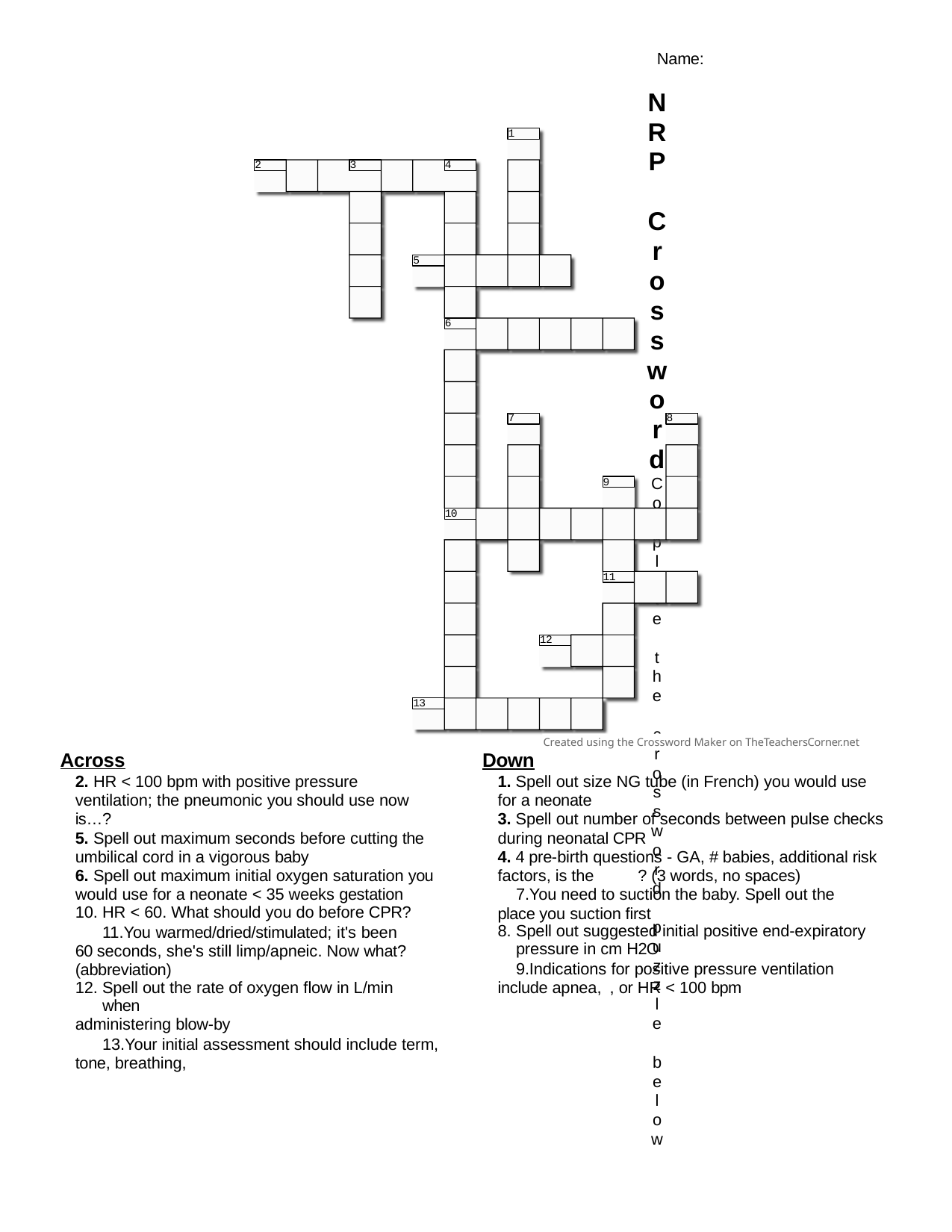

Name:
NRP Crossword
Complete the crossword puzzle below
1
2
3
4
5
6
7
8
9
10
11
12
13
Created using the Crossword Maker on TheTeachersCorner.net
Across
2. HR < 100 bpm with positive pressure ventilation; the pneumonic you should use now is…?
5. Spell out maximum seconds before cutting the umbilical cord in a vigorous baby
6. Spell out maximum initial oxygen saturation you would use for a neonate < 35 weeks gestation
HR < 60. What should you do before CPR?
You warmed/dried/stimulated; it's been 60 seconds, she's still limp/apneic. Now what? (abbreviation)
Spell out the rate of oxygen flow in L/min when
administering blow-by
Your initial assessment should include term, tone, breathing,
Down
1. Spell out size NG tube (in French) you would use for a neonate
3. Spell out number of seconds between pulse checks during neonatal CPR
4. 4 pre-birth questions - GA, # babies, additional risk factors, is the 	? (3 words, no spaces)
You need to suction the baby. Spell out the place you suction first
Spell out suggested initial positive end-expiratory pressure in cm H2O
Indications for positive pressure ventilation include apnea, 	, or HR < 100 bpm
